# Using community photography to investigate phenology: A case study of coat molt in the mountain goat (*Oreamnos americanus*) with missing data

**DOI:** 10.1002/ece3.6954

**Published:** 2020-11-09

**Authors:** Katarzyna Nowak, Joel Berger, Amy Panikowski, Donald G. Reid, Aerin L. Jacob, Greg Newman, Nicholas E. Young, Jon P. Beckmann, Shane A. Richards

**Affiliations:** ^1^ The Safina Center Setauket‐East Setauket NY USA; ^2^ Canadian Parks and Wilderness Society Yukon Whitehorse YT Canada; ^3^ Wildlife Conservation Society Bronx NY USA; ^4^ Department of Fish, Wildlife and Conservation Biology Colorado State University Fort Collins CO USA; ^5^ Eshowe South Africa; ^6^ Wildlife Conservation Society Canada Whitehorse YT Canada; ^7^ Yellowstone to Yukon Conservation Initiative Canmore AB Canada; ^8^ Natural Resource Ecology Laboratory Colorado State University Fort Collins CO USA; ^9^ School of Natural Sciences University of Tasmania Hobart TAS Australia

**Keywords:** citizen science, climate change, community science, elevation, latitude, molting, sex differences, ungulates

## Abstract

Participatory approaches, such as community photography, can engage the public in questions of societal and scientific interest while helping advance understanding of ecological patterns and processes. We combined data extracted from community‐sourced, spatially explicit photographs with research findings from 2018 fieldwork in the Yukon, Canada, to evaluate winter coat molt patterns and phenology in mountain goats (*Oreamnos americanus*), a cold‐adapted, alpine mammal. Leveraging the community science portals iNaturalist and CitSci, in less than a year we amassed a database of almost seven hundred unique photographs spanning some 4,500 km between latitudes 37.6°N and 61.1°N from 0 to 4,333 m elevation. Using statistical methods accounting for incomplete data, a common issue in community science datasets, we identified the effects of intrinsic (sex and presence of offspring) and broad environmental (latitude and elevation) factors on molt onset and rate and compared our findings with published data. Shedding occurred over a 3‐month period between 29 May and 6 September. Effects of sex and offspring on the timing of molt were consistent between the community‐sourced and our Yukon data and with findings on wild mountain goats at a long‐term research site in west‐central Alberta, Canada. Males molted first, followed by females without offspring (4.4 days later in the coarse‐grained, geographically wide community science sample; 29.2 days later in our fine‐grained Yukon sample) and lastly females with new kids (6.2; 21.2 days later, respectively). Shedding was later at higher elevations and faster at northern latitudes. Our findings establish a basis for employing community photography to examine broad‐scale questions about the timing of ecological events, as well as sex differences in response to possible climate drivers. In addition, community photography can help inspire public participation in environmental and outdoor activities specifically with reference to iconic wildlife.

## Graphical abstract

We combined data extracted from community‐sourced, spatially explicit photographs with research findings from fieldwork in the Yukon, Canada, to evaluate winter coat molt patterns and phenology in mountain goats (*Oreamnos americanus*). Using statistical methods accounting for incomplete data—a common issue in community science datasets—we evaluated the effects of intrinsic and environmental factors on molt. Our findings that males molt first and before females and that females with offspring molt last were consistent between our focal research data in the Yukon and community‐sourced data, which, being more geographically broad, enabled us to also find that molt occurs later at higher elevations and is faster at northern latitudes.

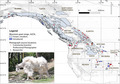

## 1 INTRODUCTION

Phenology, the seasonal timing of life history events, is increasingly relevant in the framework of global change studies (Cohen et al., 2018; Horton et al., 2020; Staudinger et al., 2019). In general, species are predicted to exhibit phenological shifts across wide geographical scales in response to climate change. Phenological responses are predicted to be particularly important for high elevation communities (Hodkinson, 2005; Stewart et al., 2019), wildlife at northern latitudes (Berger et al., 2018) and cold‐adapted species prone to seasonal mismatches, for example, in coat color (e.g., white snowshoe hares on a brown background; Mills et al., 2013; Pedersen et al., 2017; Zimova et al., 2014, 2018), or arrival at calving grounds (e.g., caribou arriving after spring vegetation flush; Post & Forchhammer, 2008).

Many mammals of temperate zones experience high seasonal variance in exposure to ambient temperature. Growth and subsequent molting of pelage, either on an annual or biannual cycle, is a common strategy used by mammals to help them regulate heat exchange in response to variation in temperature, as well as providing seasonal camouflage and opportunities for mate choice (Beltran et al., 2018). Despite the high visibility of massive chunks of hair hanging from species like bison (*Bison bison*) and muskoxen (*Ovibos moschatus*) (Berger & Cunningham, 1994; Wilkinson, 1974), for most species little is known about the phenology of shedding, or the extent to which it varies across broad latitudinal or altitudinal gradients (Beltran et al., 2018). Nonetheless, it is well established that photoperiod and, to a lesser extent, temperature control molt phenology (Lincoln & Ebling, 1985; Mo et al., 2006; Murray, 1965; Zimova et al., 2018). The timing and rate of molt may also be influenced by body condition, which depends on resource availability and reproductive output. For example, there is evidence that animals in relatively poor body condition may molt at a slower rate (Beltran et al., 2018). Déry et al. (2019) observed delayed molting of up to two weeks for both sexes of the mountain goat (*Oreamnos americanus*) during years associated with poor quality vegetation. Déry et al. (2019) also showed that molt was delayed for lactating mountain goat females, likely because, as documented in red deer (*Cervus elaphus*), the costs of milk production affect female body condition, even though food may be most abundant in summer (Clutton‐Brock et al., 1982). A better understanding of the feedbacks between environmental conditions, animal behavior and condition, and molt phenology is needed to improve our ability to predict potential impacts of environmental change on cold‐adapted species (Beltran et al., 2018).

Community generated datasets offer a promising way to test hypotheses about phenology across broad geographical ranges and temporal scales, in part because some historical data are available, and because volunteer monitoring is growing in popularity (Cooper et al., 2014; MacPhail & Colla, 2020; Taylor et al., 2019). This community‐based approach could be made better use of to complement long‐term research. For instance, community science data have been combined with satellite data to examine how bird migration (arrival time at breeding grounds) responds to advancing vegetation green‐up dates (Mayor et al., 2017). Community science data have also been explored across multiple projects to assess climate change effects on American pika (*Ochotona princeps*), for example, their site occupancy, with reasonably reliable results (Moyer‐Horner et al., 2012). In addition to mammals and birds, community‐contributed photographs have been used to document glacial retreat, and show promise to shift climate change conversations and enhance public education and engagement (Mullen et al., 2013).

There are issues to consider when relying on community sourcing of data that may be influenced by variation in identification skills, sampling effort and efficiency (Dickinson et al., 2010). Large data sets and applying appropriate statistical models may help account for potential bias (e.g., observer error in assigning sex to an animal and variation in sampling effort). Data are also often missing from community science datasets (e.g., some response variables may not have values available for all the predictor variables). The best ways to deal with missing data often associated with community science projects have received relatively little attention, and the most common approach is to simply filter out such observations (Dickinson et al., 2010), despite it being well known that nonrandom filtering of data can lead to bias and poor inference (Nakagawa, 2015). We explore the benefits of using known predictor values to help infer the likely values of predictors with missing information so that more observations can be included in the fitting process.

Mountain goats offer an unusual opportunity to examine the value of community science as an approach for investigating molt phenology because they occur along latitudinal and elevational gradients that vary in ambient conditions such as temperature and daylength. Mountain goats occupy mountainous terrain in northwestern North America (Chadwick, 2002; White et al., 2018) and make use of snow patches for cooling (Sarmento et al., 2019). They molt once per year and have thick, two layered winter coats, which can grow over ten centimeters long (Foresman, 2012). Their feeding and reproductive activities can result in variation in body condition, both among and between the sexes (Déry et al., 2019). Because of their stature as an iconic mammal of the mountains, and their dramatic seasonal change in pelage, they have for many years attracted high interest from professional and amateur photographers.

We made use of community photography, an often underutilized data source, to characterize long‐term phenological patterns of molt in mountain goats across geographical gradients, and between the sexes. Community‐sourced data spanned decades of photographs of molting goats collected by community scientists along gradients of latitude and elevation across mountain goat range. We also incorporated a comparative study design involving fieldwork of our own that concentrated on captive known individuals and adjacent wild populations at the far northern extent of mountain goat range (Yukon, Canada). Photographs provided within and between‐season estimates of the proportion of pelage shed, which were then fit to a model of molt using Bayesian methods. This model estimated the peak rate of shedding and the corresponding day of shedding, and quantified their relation to latitude, elevation, sex, and reproductive state (i.e., if a female is associated with a new kid); the latter two predictors correlating with animal condition. Missing predictor data is an issue for our study because animal sex is not always clearly distinguishable in community‐sourced photographs nor is whether or not a female is associated with a kid. Here, we develop a statistical model of coat shedding that infers the most likely state of an animal when sex and parenting status are unknown, without which a large portion of the photographs could not have been used. We assessed the utility of our statistical approach by comparing our predictions with and without complete information. We also compared our overall findings with a recently published longitudinal study of mountain goats (Déry et al., 2019). Our objective was to test whether community‐sourced data can complement scientific studies of phenology. Our results demonstrate that indeed, community science can help to identify important environmental predictors of molt (e.g., elevation and latitude), the influence of the state of the animal (e.g., sex, whether caring for young), and quantify the extent of geographical variation in molting.

## 2 METHODS

### 2.1 Community science

Photographs were sourced from members of the public including staff, researchers, and visitors to parks and protected areas, professional photographers, hunters and guide‐outfitters, and other outdoor enthusiasts. Criteria for photograph submissions included known date and location, animal clearly visible and ideally from the side, high enough image resolution (desired value of 300 dpi) to use pixel counts to estimate shed extents.

To encourage photograph submissions, we used the online platforms CitSci and iNaturalist, hung posters in public places, used social media and word of mouth. To further crowd‐source mountain goat images, we also used forums and listservs of wildlife agencies and professional societies such as the Yukon Fish and Game Association and British Columbia Wildlife Federation, as well as radio (Mountain FM and Canadian Broadcasting Corporation), and local newspapers (e.g., *Hungry Horse News*). Since all photographs submitted via CitSci become party to a Creative Commons license, we also gave the option to email us photographs, and this was often preferred by professional photographers. If sourcing from iNaturalist, we contacted the photographer and asked for permission before including their photograph in our analysis. We also received (by mail) slides and photographs developed from film from both photographers and visitors to protected areas, as well as (digital) photographs from remote cameras, particularly from agency staff and researchers working in parks. Photographs submitted from hunters were limited since the hunting season spans the period when mountain goats are in full winter coats (between October and April). Some professional photographers also expressed preference for photographing goats in winter months when the animals are “more photogenic” (i.e., not patchy with molting fur).

### 2.2 Fieldwork in southern Yukon

We deployed remote cameras between mid‐May and early September 2018 to develop sex and latitudinal chronologies of mountain goat molt in the far north for contrasts with southern goat populations. Our study areas included three locations at which mountain goats are wild (Mount White (60.2°N, −133.9°W), Montana Mountain (60.0°N, −134.6°W), and Kluane National Park (60.7°N, −137.7°W)), and a fenced facility, the Yukon Wildlife Preserve (YWP) (60.8°N, −135.3°W), where 20 goats (in two herds, one breeding, one nonbreeding) roam large enclosures and are viewed by the public. We deployed 16 cameras (the limited number of cameras that we had access to) along active mountain goat trails in these four locations (four cameras per location). We also took photographs at the YWP weekly of all visible captive animals to complement the camera trap data. Our data from the wild (opportunistic camera trap photographs) were added to the community science dataset, whereas our repeated samples data on 14 (of the 20) known captive adult mountain goats in YWP were analyzed separately.

### 2.3 Sample of photographs

**Figure 1:**
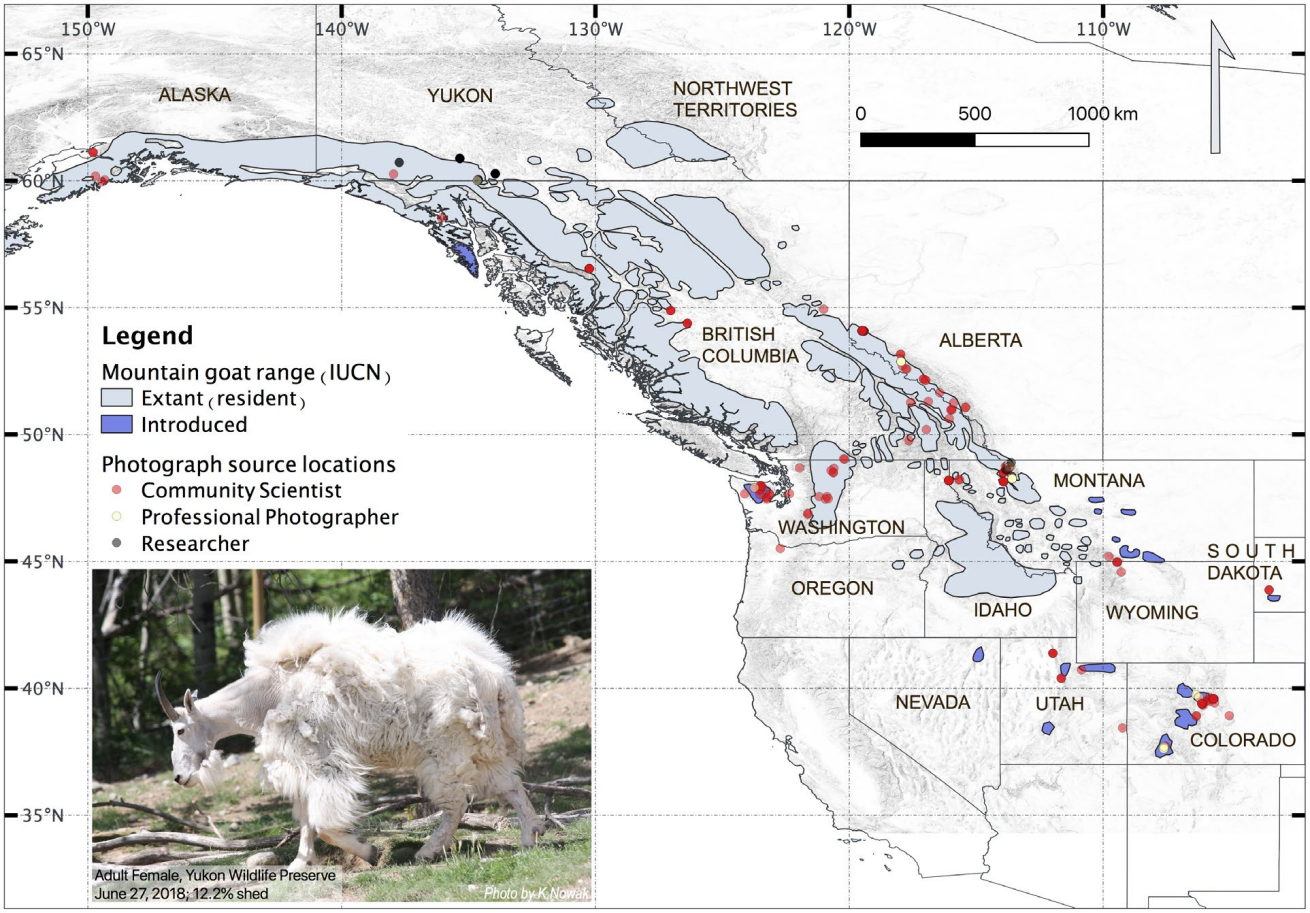
Map showing mountain goat range (IUCN Red List shapefile 2008) and locations of photographs from community scientists (red), professional photographers (also considered community scientists but shown separately here, in yellow), and researchers (gray). A couple of the points outside the range as shown are from zoos (Woodland Park Zoo and Oregon Zoo); there are also photographs from several areas where goats were introduced (e.g., Mount Peale, Utah) that are not part of the available shapefile. The photograph shows an adult female in the Yukon Wildlife Preserve on 27 June 2018 with 12.17% of her winter coat shed (with molt still at an early stage, demonstrably starting at the head and proceeding down the neck)

**Figure 2:**
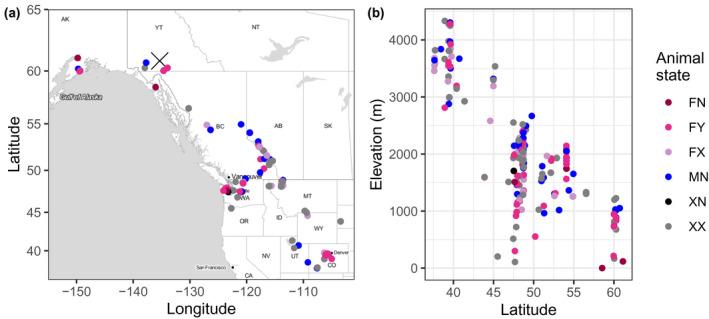
(a) Locations of community‐sourced photographs. Cross indicates the location of the captive study population at the Yukon Wildlife Preserve (YWP). (b) Relation between latitude and elevation for all photographs. Colors show what is known about the sex and presence of a kid for each animal photographed. There are six possible animal states described by a pair of letters: [first pair] F = female, M = male, X = sex unknown; [second pair] Y = with kid, N = without kid, X = kid status unknown: FN = female without kid, FY = female with kid, FX = female and presence of kid unknown, MN = male, XN = unknown sex without kid, and XX = unknown sex and presence of kid unknown

We amassed more than 800 photographs of which more than 100 were omitted before any processing for the following reasons: They were determined to be potential duplicates (photographs of the same individual on the same day), insufficient side‐facing orientation of the goat, poor photograph quality or resolution, or goat obscured by foliage, rocks or offspring. We then analyzed 693 photographs of which nearly 80% were community‐sourced and spanned years 1948 to 2018 from the entire distributional range of mountain goats (Figures 1 and 2) with the exception of Nevada and the Northwest Territories (NWT). Broken down by sources of data, professional photographers were our main source of photographs (*N* = 203) followed by the CitSci platform (185), iNaturalist (126), researchers (Caw Ridge, Alberta, and Glacier National Park (GNP), Montana: 58), members of the public by e‐mail (15), members of professional societies such as the B.C. Mountain Goat Society, Wilderness Society, and Summit Post (13), and other sources (4). Our 2018 southern Yukon sample included 58 photographs from captivity and 31 from the three wild sites.

Mountain goats at GNP (where mountain goats are habituated in some locations) were most photographed by the public and by one professional photographer in particular (Sumio Harada; see Harada, 2018), followed by Mt. Evans, Colorado (where mountain goats are introduced and also habituated to people), while Caw Ridge and Yukon were third and fourth most common data sources with photographs provided by researchers (including the authors of this paper and inclusive of camera trap photographs).

### 2.4 Photograph analysis

To estimate the extent of winter coat shed, we compared pixel counts of shed versus unshed areas of mountain goats’ coats in each photograph using Adobe Photoshop. First, we outlined the entire animal, typically using a combination of the quick selection and lasso tools (omitting hooves, eyes, nose, mouth, and horns), copied the animal, separated from its background, into a new layer (total layer). Second, we duplicated this layer, and outlined the shed and unshed areas and cut these as new layers (shed and unshed layers). We selected all pixels in the layers and used the histogram tool to obtain counts of both shed and unshed areas. Lastly, to create quick visuals, we filled the unshed area with red and the shed area with black; a video tutorial of our approach can be found here: https://www.youtube.com/watch?v=h9cWl9Z1Odw. Four of us meticulously scored the molt including the PI, who conducted routine checks to ensure each observer maintained >85% interobserver reliability with the PI; where challenging photographs led to bigger discrepancies, they were omitted from analysis (*N* = 8).

Before the PI sexed mountain goats in photographs, she practiced with both the online mountain goat identification quiz (Alaska Department of Fish and Game) and on the captive mountain goats at YWP. The presence of a kid in close physical contact with an adult was also used as a proxy for adult sex and, if the adult was determined to be female (primarily on the basis of horn thickness and basal horn diameter), she was assumed to be the kid's mother (we are not aware of allomothering in wild mountain goats).

Our noninvasive, photograph‐based molt analysis is not without precedent: Vieira et al. (2017) used photographs to evaluate feather molt in black skimmers (*Rynchops niger*), and Beltran et al. (2019) used photographs to study molt in Weddell seals (*Leptonychotes weddellii*).

### 2.5 Statistical analysis

A statistical model was developed to describe seasonal and long‐term molt dynamics. Specifically, we quantified intrinsic (sex and presence of offspring) and broad environmental factors (latitude and elevation) on molt timing and rate. We used latitude and elevation as predictors as they were available for all photographs and considered to be good proxies for the suit of factors likely to directly and indirectly affect molt (e.g., temperature, resource availability, and photoperiod). Even if photographs were submitted with elevation data, we used provided georeferenced locations of all photographs to source elevation in meters for each photograph from the Global Multi‐resolution Terrain Elevation Data 2010 (GMTED2010).

We assumed that the progression of the amount of coat shed within a season could be described by the logistic equation. Let *f*(*t*) denote the mean fraction of coat shed on day of year (DOY), *t*. In its simplest form, our model is given by:

$$f\left(t\right)=\frac{e^{\alpha (t‐\tau )}}{1+e^{\alpha \left(t‐\tau \right)}}$$

where *τ* is the DOY when 50% of an animal's coat has been shed, and *α* describes the rate of shedding. Rate of shedding peaks when *t* = *τ* and has value *α*/4 (fraction of coat shed per day). We refer to *τ* and *α* as the shedding date and shedding rate, respectively. These two parameters may be affected by animal state or environmental variables. Here, the state of an animal is defined by its sex and, if female, the presence of a kid. Although animal state is known for the captive study, it is often not clear from community photographs. Animal state is described by a letter pairing; the first letter describes the sex of the animal (F = female, M = male, X = unknown), and the second letter describes the presence of kid (Y = yes, N = no, X = unknown). Assuming only females may be associated with kids, there are six animal states, three of which are unambiguous: FN (female without a kid), FY (female with a kid), and MN (male without a kid). The three ambiguous states are FX (female but unclear if kid present), XN (sex unknown and no kid present), and XX (sex and presence of kid unknown). We assume that animal state may affect the timing and rate of shedding. Let *τ*_0_ be the shedding date of a female without kid, and suppose the shedding date of males differs to females by *τ*_M_. Shedding date of females when with kid differs by *τ*_K_. Similarly, let *α*_0_, *α*_M,_ and *α*_K_ denote the rate of shedding for a female without kid, and the change in shedding rate when male, or when a female with kid.

Shedding rate and shedding date may also correlate with elevation (E) and latitude (L), due to their relation with temperature and photoperiod. Specifically, for an animal photographed in year *y* at elevation *x*_E_ and latitude *x*_L_, we assume

$$\alpha =\alpha_{0}+\alpha_{M}x_{M}+\alpha_{K}x_{K}+\alpha_{E}x_{E}+\alpha_{L}x_{L}+\alpha_{Y}y+\alpha_{\left[y\right]},$$

and

$$\tau =\tau_{0}+\tau_{M}x_{M}+\tau_{K}x_{K}+\tau_{E}x_{E}+\tau_{L}x_{L}+\tau_{Y}y+\tau_{\left[y\right]},$$

where *x*_M_ and *x*_K_ are binary predictors indicating whether the animal is male, or with kid (e.g., if animal state is FY then *x*_M_ = 0 and *x*_K_ = 1). Year factors into the model as both a continuous predictor and a random factor, as indicated by the last two linear terms used to calculate *α* and *τ*. Parameters *α*_Y_ and *τ*_Y_ describe long‐term, smooth trends in shedding rate and date, which we might expect to differ from zero under climate change. Alternatively, the *α*[*y*] and *τ*[*y*] are random effects associated with year *y*, drawn from *t*‐distributions with mean zero, standard deviation *σ_α_* and *σ_τ_*, degrees of freedom *η_α_* and *η_τ_*, respectively, and represent any year‐specific stochastic effects on the rate and timing of shedding that are common to all locations that year (e.g., large‐scale weather fluctuations).

We acknowledge that data sourced from community photographs are likely to contain sampling error that is greater than the error expected from a scientific field study. Appropriate consideration of sampling error is important because parameter estimates can be sensitive to the assumption of the error distribution (Richards, 2008). In our case, variation in animal orientation is likely to add error in the true fraction of coat shed, especially when animals are just starting to shed or shedding is near completion. We accounted for this added uncertainty by grouping the observed estimates of coat shed into 25 equal‐sized bins, each representing 4% of the coat, and fitted our model to this more regularly distributed data. We checked that our choice of bin size did not affect our final conclusions by also fitting the model using 1% bins (see Results). Values for the fitted response variable were set to *n* = round(*fN*), where *f* is the digitized estimates of the fraction shed and *N* is the number of shedding bins, implying 0 ≤ *n* ≤ *N*. The probability of observing *n* bins shed at time *t* when the expected fraction shed is *f*(*t*), is given by the beta‐binomial distribution, denoted *P*_BB_(*n*). The beta‐component accounts for overdispersion in the observations relative to the binomial distribution, which might be due to variation in animal orientation, or shedding effects due to unknown covariates (such as age, which we did not consider). We formulated the beta‐binomial using the parameter *ϕ* (common to all observations) so that its variance inflation factor, relative to the binomial distribution, is $v=1+\left(N‐1\right)\phi /\left(1+\phi \right)$ (Richards, 2008).

The model described above can be fit to observations of shedding where animal state is unambiguous. However, we can also fit this model to observations when animal sex or presence of kid is unknown. Suppose at any time proportion *p* of animals are female and proportion *q* of the females are associated with a kid. These assumptions imply that the average proportion of animals in states: FN, FY, and MN, are *p*(1‐*q*), *pq*, and (1‐*p*), respectively. We checked that the frequencies of animal states were consistent with our assumptions of constant *p* and *q* across years (Supplementary materials). When animal state is ambiguous (i.e., FX, XN, or XX) the probability of observing *n* shed bins is a weighted sum of the three beta‐binomial distributions associated with the unambiguous states, where the weights are calculated using *p* and *q*. Specifically,

$$\mathrm{Pr}\left(n|\text{FX}\right)=\left(1‐q\right)P_{\text{BB}}\left(n|\text{FN}\right)+qP_{\text{BB}}\left(n|\text{FY}\right),$$

$$\mathrm{Pr}\left(n|\text{XN}\right)=\frac{p\left(1‐q\right)P_{\text{BB}}\left(n|\text{FN}\right)+\left(1‐p\right)P_{\text{BB}}\left(n|\text{MN}\right)}{p\left(1‐q\right)+\left(1‐p\right)},$$

and

$$\mathrm{Pr}\left(n|\text{XX}\right)=p\left(1‐q\right)P_{\text{BB}}\left(n|\text{FN}\right)+pqP_{\text{BB}}\left(n|\text{FY}\right)+\left(1‐p\right)P_{\text{BB}}\left(n|\text{MN}\right),$$

where *P*_BB_(*n*|*j*) is the probability of observing *n* bins shed when the animal is in an unambiguous state *j*, which is calculated according to the beta‐binomial distribution, as described above.

This model has 19 parameters (see Table 1), and we estimate them using Bayesian methods based on Monte Carlo sampling. We used R (R Core Team, 2020) with rstan (Stan Development Team, 2020) to perform the statistical analyses (Supplementary materials). Elevations and latitudes were z‐transformed before fitting to reduce parameter correlations and help with posterior parameter convergence. We specified relatively uninformative priors for all parameters so that the posterior distributions were strongly dependent on the data (Table 1). We used three sampling chains to visually check that our model formulation converged and posterior parameter distributions were based on 1,000 samples after a 1,000 sample burn‐in. Parameter uncertainty was assessed using 89% credible intervals (McElreath, 2016). We checked the utility of our novel approach for incorporating photographs missing animal state information by comparing its predictions with those made from the model when fit only to unambiguous photographs.

**Table 1:** Posterior parameter estimates for models fit to the community science project (CSP) and the captive Yukon Wildlife Preserve (YWP) study

Shedding bins, *N*		Community Science Project			Y WP (captive) Study
		25	100	25	25
Data source		All photographs, including ambiguous states	All photographs, including ambiguous states	Only unambiguous states	All photographs, all states are unambiguous
Observations (photographs)		562	562	329	58 (14 individuals)
Parameter	Prior	Posterior estimate: median [89% credible interval]			
*p*	B(2,2)	0.666 [0.633,0.696]	0.667 [0.632,0.697]	NA	NA
*q*	B(2,2)	0.500 [0.463,0.538]	0.501 [0.461,0.538]	NA	NA
*τ*_0_	B(2,2)	0.533 [0.525,0.543]	0.530 [0.521,0.539]	0.533 [0.520,0.545]	0.542 [0.529,0.555]
$\tau_{M}^{*}$	N(0,0.1)	**−4.38** [−6.93,−1.82]	**−4.38** [−6.93,−2.19]	**−6.57** [−10.59,−2.19]	**−29.20** [−38.69,−18.61]
$\tau_{K}^{*}$	N(0,0.1)	**6.20** [4.02,8.76]	**6.20** [3.65,8.39]	**12.78** [8.76,16.79]	**21.17** [11.31,31.39]
*τ*_Y_	N(0,0.1)	−0.001 [−0.009,0.007]	−0.001 [−0.010,0.007]	0.002 [−0.006,0.011]	NA
*τ*_E_	N(0,0.1)	**0.008** [0.003,0.013]	**0.010** [0.005,0.015]	0.000 [−0.008,0.008]	NA
*τ*_L_	N(0,0.1)	−0.001 [−0.006,0.004]	0.001 [−0.004,0.006]	−0.007 [−0.014,0.001]	NA
*α*_0_	N(25,5)	22.3 [20.4,24.4]	22.8 [20.8,24.9]	26.2 [23.0,28.8]	32.5 [27.6,36.9]
*α*_M_	N(0,20)	1.3 [−1.3,4.0]	1.3 [−1.2,3.9]	−0.2 [−3.8,4.0]	**35.9** [21.1,53.4]
*α*_K_	N(0,20)	−0.8 [−3.0,1.6]	−1.3 [−3.5,1.0]	**−3.6** [−4.9,−0.5]	−3.7 [−12.2,5.1]
*α*_Y_	N(0,1)	0.062 [−1.056,1.151]	0.43 [−0.664,1.441]	−0.133[−1.380,1.147]	NA
*α*_E_	N(0,1)	0.158 [−0.858,1.218]	−0.275 [−1.328,0.749]	0.475 [−0.834,1.636]	NA
*α*_L_	N(0,1)	**1.549** [0.451,2.722]	**1.631** [0.492,2.656]	0.515 [−0.678,1.744]	NA
*ϕ*	E(1)	0.264 [0.239,0.291]	0.333 [0.307,0.365]	0.193 [0.163,0.237]	0.039 [0.009,0.101]
*σ_t_*	E(10)	0.014 [0.009,0.024]	0.015 [0.009,0.026]	0.011 [0.004,0.024]	NA
*σ_a_*	E(1)	1.399 [0.353,2.783]	1.227 [0.188,2.703]	1.715 [0.371,4.420]	NA
*σ*_ID_	E(10)	NA	NA	NA	0.019 [0.009,0.032]
*n_t_*	G(2,0.1)	18.7 [5.3,48.7]	19.5 [5.7,47.2]	17.0 [4.0,48.1]	NA
*n_a_*	G(2,0.1)	16.8 [3.9,46.7]	16.9 [4.2,45.8]	15.3 [3.1,45.1]	NA
*n*_ID_	G(2,0.1)	NA	NA	NA	18.4 [5.0,48.8]

Note

Three sets of parameter estimates are presented for the CSP demonstrating the insensitivity of choice of the number of shedding bins, *N*, and the effect of only considering photographs where the sex and presence of a kid is known. The timescale is per year, and elevation and latitude have been z‐transformed. Estimated state‐dependent shifts in molt date, denoted *, have been converted to days. Bold values depict parameters where zero indicates no effect and the posterior 89% credible interval does not include zero. Prior credibility distributions are also presented. Normal distribution with mean = *μ* and variance = *σ* ^2^: N(*μ*,*σ*); beta distribution with mean = *a*/*a* + *b* and variance = *ab*/(*a* + *b*)^2^(*a* + *b*+1): B(*a*,*b*); exponential distribution with mean = 1/*a* and variance = 1/*a* ^2^: E(*a*); and gamma distribution with mean = *a*/*b* and variance = *a*/*b* ^2^: G(*a*,*b*). Note that not all parameters are estimated for each example.

For the captive (YWP) portion of our study, we fit a simpler form of the model. As this study was conducted at a single site, and within a single season, we did not estimate effects of elevation, latitude or year (i.e., *α*_E_ = *α*_L_ = *α*_Y_ = *τ*_E_ = *τ*_L_ = *τ*_Y_ = 0). An important difference with sampling design between both studies is that animals were identified and repeatedly surveyed in the captive (YWP) study. A random effect term associated with each animal was used to account for repeated measures; however, given the limited number of animals in the study (14), we only included a single random effect and associated it with shedding date. These random effect terms were drawn from a t‐distribution with mean zero, standard deviation, *σ*_ID_, and degrees of freedom, *η*_ID_. The model of shedding that we fit to the captive study had seven parameters (Table 1). R and stan code used for fitting both models are provided in Supplementary materials.

For statistical analysis purposes, we reduced our sample of 693 processed photographs to photographs of adult animals estimated to be at least two years old (on the basis of body size and horn length) and we focused on photographs taken between the months of May to September. Please refer to the results sections for details on further sample size reductions.

## 3 RESULTS

**Figure 3:**
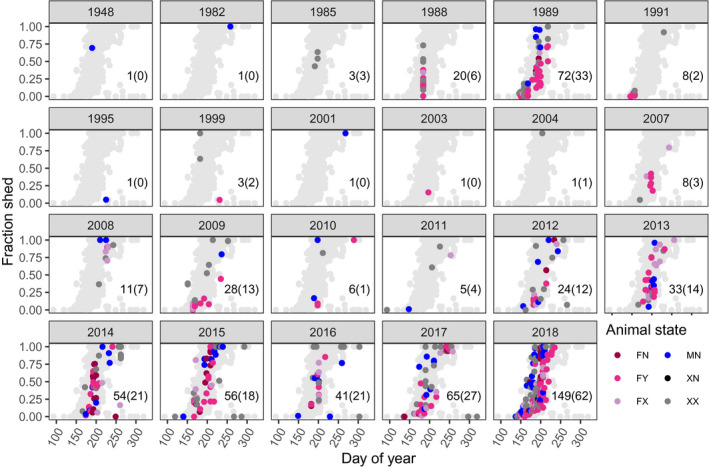
Fraction of coat shed estimates from all photographs collected during the community science project. The number of photographs taken each year and the number where animal state is uncertain (brackets) are provided in the panels. See Figure 2 for explanation of animal state. Light gray circles depict all shedding estimates. Note that the unusual late‐year, low‐shed values were removed from the analysis. The model was only fit to years where there was at least one photograph where animal state was not ambiguous (i.e., bracketed value is greater than zero)

We had 651 photographs where shedding fraction could be well estimated and photographs met our other criteria. Photographs provided good spatial coverage as they spanned latitudes 37.6°N to 61.1°N and elevations between 0 m at Glacier Bay, Alaska and 4,333 m in the southern Rocky Mountains of Colorado, USA. Latitude and elevation were negatively correlated (*r* = −.783, 95%CI [−0.812,‐0.749], Figure 2). Community‐submitted photographs were taken between the years 1948 and 2018; but, as expected, dates were heavily biased toward the latter few years (Figure 3). Sex and status with/without kid could only be attributed to 55% of the photographs, and most photographs were of females with kids (sample sizes: FN = 94, FY = 202, FX = 61, MN = 104, XN = 2, XX = 188).

Animals predominantly shed over a 3‐month period between day of year (DOY) 150 (29 May) and DOY 250 (6 September) (Figure 3). The photographs indicate that, on average, males shed earlier than females and females with kids tend to shed later than females without kids (Figure 3). There was no clear pattern of long‐term trends in shedding (Figure 3).

We noted that shedding estimates for 16 animals were unusually low and late in the season (i.e., after DOY 220, Supplementary materials). These photographs were removed from the statistical analysis as they do not reflect typical shedding patterns and will likely result in biased parameter estimates. Our model incorporates interannual variation in shedding rate and date that involves estimating annual deviations drawn from t‐distributions. These estimates are highly uncertain for years when no animal state is known with certainty, so we did not include these years in the analysis. Our final sample size was 562 photographs from 14 years spanning 1988–2018, and for 329 of these photographs animal state was known.

**Figure 4:**
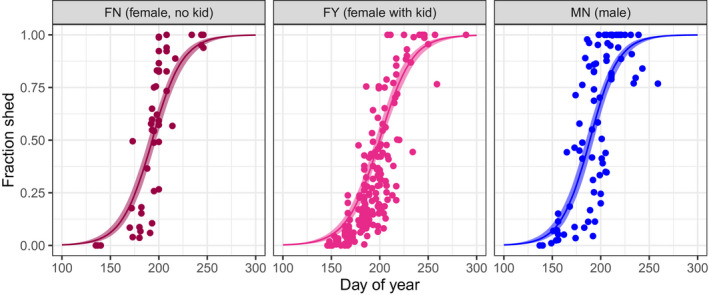
Observed and predicted shedding patterns. Panels correspond to the three animal states where sex and kid status are known. Shedding fractions for all photographs where animal state was known are presented (points). The predictions are for 2018 at location defined by the z‐transformed predictors being zero, which corresponds to latitude 49.14 and elevation 2,025 m (c.f. Figure 2b). The predictions also correspond to all random effect terms being set to zero. Solid lines depict the median shedding fraction, and shaded regions are the associated 89% credible intervals

**Figure 5:**
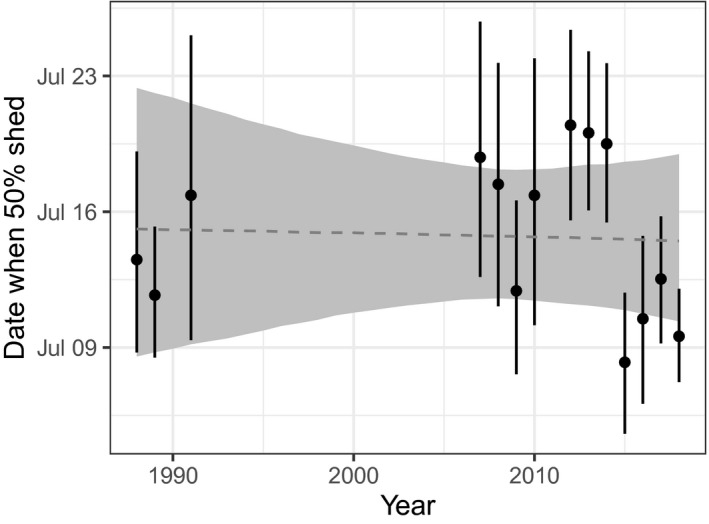
Predicted dates for females without kid (state FN) having shed 50% of their coat when at a site defined by *z*‐transformed predictors being zero (i.e., latitude 49.14 and elevation 2,025 m). Dashed line is the long‐term trend, and shaded region is the 89% CI. Estimated yearly fluctuations about the trend are also presented along with their 89% CI

Our statistical model when fit to all 562 photographs for *N* = 25 was able to reproduce the observed patterns of shedding (Figure 4). The model estimated that about two‐thirds of animals photographed were female and half of those had a kid (Table 1; *p* = .666, *q* = 0.500). There was strong evidence that males shed before females by about *τ*_M_ = 6.4 days, and females shed later when with kid by about *τ*_K_ = 5.5 days. The model did not find evidence of a long‐term trend in either the date or rate of shedding (Figure 5); 89% credible intervals (CIs) for *τ*_Y_ and *α*_Y_ contained zero (Table 1). There was weak evidence that the mean date of shedding varied stochastically between years (Figure 5). Shedding date was positively associated with elevation (i.e., delayed), and shedding rate was faster at higher latitudes (Table 1).

Parameter estimates and uncertainty associated with the estimates were largely unchanged when we assumed *N* = 100 shedding bins (Table 1). When we fit our model only to data taken from photographs where animal state was known (i.e., 329 photographs), we were unable to detect elevation or latitude effects on shedding date or rate, although we were still able to detect early shedding for males and delayed shedding for females with kid (Table 1).

**Figure 6:**
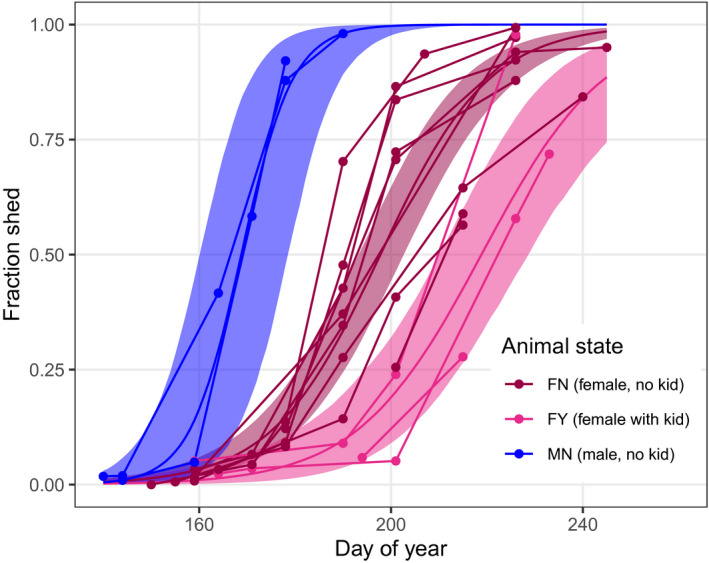
Observed shedding patterns for 14 captive animals repeatedly observed at the Yukon Wildlife Preserve in 2018. See Figure 2 for explanation of animal state. Shaded region depicts 89% CI for the mean fraction shed

Although the number of animals observed during the study at the captive site was very low (animal numbers: FN = 9, FY = 3, MN = 2), the 58 photographs suggest that males shed before females and females with kids had delayed shedding (Figure 6). Model fitting again supported animal state as being an important determinant of the timing of shedding (Table 1). In this case, on average, males were estimated to shed 23.7 days earlier than females and the presence of a kid delayed female shedding date by 17.9 days. These offsets are greater than those predicted by the community science analysis and may be the result of biases due to low sample sizes for the captive study, or uncertainty in animal state inherent with the community data. In addition, the model found evidence that males shed faster than females, however females with and without kids shed at similar rates (Table 1).

## 4 DISCUSSION

We found evidence that male mountain goats, on average, molt earlier than females, and females with kids tend to delay their molt. These findings were consistent when we fit our model to the community‐sourced data and the captive YWP data (Table 1). We also found evidence that mountain goats delay molt at higher elevations and molt faster at higher latitudes. Males at the YWP also molted at a faster rate than the females. The community photography analysis suggests that these molt patterns are consistent across mountain goats’ natural and introduced range (Figures 1 and 4), and have been relatively consistent for at least 30 years (Figures 3 and 5).

Our findings on sex differences and effects of new (current year's) kids on the timing of molt for mountain goats are consistent with published results from Caw Ridge, west‐central Alberta (Déry et al., 2019), as well as with the Traditional Knowledge of a Tlingit weaver (Rofkar, 2014) who observed that females finish molting only after weaning their offspring. Our results further support the reliability of using stage of molt as an indicator or proxy for animal sex and reproductive status when observing mountain goats in the field (as done by Chadwick, 2002).

Déry et al. (2019) analyzed a longitudinal dataset of mountain goat molt spanning 27 years and found evidence that molt was driven by animal condition, which in turn was influenced by the availability of high‐quality resources. Healthy adult females tended to complete molt 20 days later than males, and females with offspring molted 10 days later than females without offspring. However, females in poor condition had similar molt dates whether or not they produced a kid. These estimates for good condition animals are consistent with our estimates based on captive animals at YWP (Table 1). Corresponding effect sizes estimated from our community‐sourced data were weaker (Table 1), which might be due to greater natural variation in wild animal condition resulting from environmental variation in resource availability. Unfortunately, we did not have good estimates of animal age to look for nonlinear age‐dependent timing of molt that Déry et al. (2019) observed. Faster shedding rates that we estimated for captive males at the YWP is consistent with studies suggesting that healthy animals with low reproductive costs molt faster (Beltran et al., 2018). On this basis, we speculate that if heavy winter coats become a thermoregulatory liability as summers continue to heat up, then females with offspring may be most affected.

While our sample had broad spatial coverage (Figure 1), thanks to community‐sourced data, directly inferring the effects of latitude and elevation on molt is not straightforward due to their negative correlation (Figure 2) and their relation to factors known to directly affect molt, such as temperature and photoperiod. We did not include temperature as a predictor variable because the most appropriate temporal weighting to apply to temperature is unknown (e.g., when during the season and day temperature most impacts molt). Our model predicts delayed molt at higher elevations. Where mountain goats have access to a range of elevations (e.g., in the southern Rocky Mountains), animal movement and foraging behavior could lead to greater access to seasonal resources and mitigating of temperature extremes. Delayed molt may be the result of these behaviors leading to improved body condition and effective thermoregulation. However, inferring environmental conditions from photographs may be associated with high uncertainty as they are mere snapshots of locations visited by goats and not necessarily indicative of long‐term environmental conditions (Beever et al., 2017). Our model also predicted faster molt at higher latitudes, where seasonal variation in photoperiod is greater. Zimova et al. (2018) showed that melatonin increases as day length shortens, which inhibits prolactin production, stimulating hair follicle development. Thus, detecting a latitudinal effect may not be surprising. Although inference of the mechanisms driving wide‐scale patterns of molt is beyond the scope of our study, our results provide some support of hypotheses presented by earlier studies.

High variation in molt dynamics observed in our study may be influenced by a number of other variables that were not measured. For example, we noted visible hair loss in mountain goats’ shoulder areas associated with active rubbing, which may be a reaction to ticks (*Dermacentor andersoni*), especially at Glacier National Park (the locale of most of our crowd‐sourced photographs). As natural molt starts at the face, loss of hair and irritated skin at the shoulder area when the face and neck are not yet shed can most likely be attributed to ticks and not to natural molt onset. We did not attempt to distinguish tick‐related loss of hair from regular molt. High between‐animal variation in molt may also be partly due to mountain goat introductions (including of northern animals to southern locales) and animals being associated with distinct genetic histories. Investigating differences between native and introduced populations was beyond the scope of our study because the histories of introduced individuals cannot be easily surmised from community‐sourced photographs.

We developed a statistical approach for incorporating photographs associated with incomplete information on animal state into the model fit. Individually, photographs associated with ambiguous animal state contributed relatively less to the parameter estimates; however, collectively incorporating these photographs resulted in strengthening of evidence for state dependence and revealed elevation and latitude effects (Table 1). Not surprisingly, the high variation and uncertainty associated with the wide‐scale community‐sourced data resulted in lower estimated rates of molt relative to the captive site estimates. Our statistical approach that incorporated random effects and binning to regularize the highly stochastic variation in observed shedding fractions was robust to the choice of bin size (Table 1). Our findings further support the development of statistical methods for incorporating missing ecological data.

Generally speaking, wildlife photography contains a wealth of information beyond the time, location, and presence of an organism, and is a yet relatively untapped source of ecological data. A bonus outcome of research such as ours is boosting community engagement with wildlife and climate science through active involvement in the data collection and compilation process (Cooper et al., 2014; Newman et al., 2016). Tapping into individual photograph collections that have not yet been digitized would have required more time but could have likely both enhanced our sample of older photographs and engaged persons who do not readily use online platforms.

## 5 CONCLUSIONS

We show that, in a broad sense, there is power in combining community‐sourced data and appropriate analytic techniques to understand ecological trends across broad areas and environments. Both the community science component and our focal study of captive mountain goats provided consistent predictions regarding the effect of animal state that coincide with earlier research findings, demonstrating that community science data can identify the same ecological patterns available from a planned research study. Other photograph‐based community science engagements using resources from museum and newspaper archives, personal collections, and automated photograph processing methods have also been useful, for example, for understanding the demise and re‐expansion of black bears (*Ursus americanus*) into desert environments (Lackey et al., 2013). Our project contributes to this growing knowledge base and substantiates a potential way for researchers and the public to showcase collaborative approaches to address specific scientific questions across large geographical areas.

## CONFLICT OF INTEREST

The authors have no conflicts of interest to declare.

## AUTHOR CONTRIBUTION

**Katarzyna Nowak:** Conceptualization (lead); Data curation (lead); Formal analysis (supporting); Funding acquisition (supporting); Investigation (lead); Methodology (lead); Project administration (lead); Resources (supporting); Writing‐original draft (lead). **Joel Berger:** Conceptualization (supporting); Funding acquisition (lead); Resources (supporting); Writing‐original draft (supporting); Writing‐review & editing (supporting). **Amy Panikowski:** Data curation (supporting); Investigation (supporting); Methodology (supporting); Writing‐review & editing (supporting). **Donald G. Reid:** Data curation (supporting); Investigation (supporting); Resources (supporting); Writing‐review & editing (supporting). **Aerin L. Jacob:** Funding acquisition (supporting); Resources (supporting); Writing‐review & editing (supporting). **Gregory Newman:** Data curation (supporting); Writing‐review & editing (supporting). **Nicholas E. Young:** Data curation (supporting); Writing‐review & editing (supporting). **Jon Beckmann:** Funding acquisition (supporting); Project administration (supporting); Resources (supporting); Supervision (supporting); Writing‐review & editing (supporting). **Shane Richards:** Formal analysis (lead); Methodology (supporting); Software (equal); Validation (lead); Visualization (lead); Writing‐original draft (equal); Writing‐review & editing (lead).

### OPEN RESEARCH BADGES

This article has earned an Open Data Badge for making publicly available the digitally‐shareable data necessary to reproduce the reported results. The data is available at https://doi.org/10.5061/dryad.8w9ghx3k3

## Supporting information

Supinfo1

Supinfo2

Supinfo3

Supinfo4

## Data Availability

Our data, methods, and corresponding html files are archived at: https://doi.org/10.5061/dryad.8w9ghx3k3 after removing longitude for data privacy reasons and so as not to reveal locations of sensitive areas such as mineral licks.
