# Supplementary material for: Using community photography to investigate phenology: A case study of coat molt in the mountain goat (Oreamnos americanus) with missing data
Source: Ecol Evol. 2020 Nov 9;10(23):13488–99. doi: 10.1002/ece3.6954 (PMC7713987; doi:10.1002/ece3.6954)
Supplement: Supplementary file 1 — Supinfo1 [file ECE3-10-13488-s001.pdf]

Supplementary Materials 1: Data Wrangling

Shane A. Richards

2020-09-13

Contents

Summary 1

Sampling locations 2

Shedding fractions 4

Environmental predictors 6

Inconsistent data 7

Predictor variables 8

Fitted data set 13

Summary

This document supports the manuscript:

> Nowak et al. "Using community photography to investigate phenology: a case study of coat molt in the mountain goat (Oreamnos americanus) with missing data" submitted to Ecology and Evolution.

Here we provide the wrangling of the mountain goat molting data that was collected as part of a citizen science project. Photographs were sourced from members of the public including staff, researchers, and visitors to parks and protected areas, professional photographers, hunters and guide-outfitters, and other outdoor enthusiasts. The data are stored in the file `CitizenScienceObs.csv`.

The main objective of this analysis is to quantify change in the proportion of coat shed over summer and determine whether the onset of molting has shifted over time. Prior studies, and analyses of captive animals observed during the summer of 2018 in Yukon Wildlife Preserve, suggest that the timing of molting differed between males and females (males shed earlier) and females associated with a kid shed later. Molt is known to be driven by temperature, resource availability and photoperiod, which are expected to correlate with latitude and elevation.

Here, we display the data analysed in the manuscript. We look for evidence of outliers and check model assumptions (e.g. constant proportional representation of animal states).

Sampling locations

First, the raw data is presented.

```
# == Preliminary data wrangling

rm(list = ls()) # clear memory

# load all packages needed for the analysis
library(tidyverse)
library(readxl)
library(lubridate)
library(scales)
library(ggmap)
library(cowplot)
library(GGally)

# read in the data
df_all <- read_csv("CitizenScienceObs.csv")

df_all <- df_all %>%
    mutate(InYWP = Lat > 60.87 & Lat < 60.89 & Long > -135.35 & Long < -135.33) %>%
    filter(InYWP == FALSE) %>%
    select(-InYWP)

# add variables for fitting
df_all <- df_all %>%
  mutate(
    frac_shed = ShedPixel / (ShedPixel + UnshedPixe), # calc fraction
    doy  = lubridate::yday(DateObs), # extract day of year
    year = lubridate::year(DateObs)  # extract year
  )

# replace NAs with X
df_all$Sex[which(is.na(df_all$Sex))] <- "X"
df_all$Kids[which(is.na(df_all$Kids))] <- "X"

# ensure no males will have a kid
indxMX <- which(df_all$Sex == "M", df_all$Kids == "X")
df_all$Kids[indxMX] <- "N"

# create appropriate factors
df_all$Sex  <- factor(df_all$Sex)
df_all$Sex  <- fct_relevel(df_all$Sex,  "F", "M", "X")
df_all$Kids <- factor(df_all$Kids)
df_all$Kids <- fct_relevel(df_all$Kids, "N", "Y", "X")

# add animal state variable
df_all <- df_all %>%
  mutate(SK = factor(paste(Sex, Kids, sep = "")))
df_all$SK <- fct_relevel(df_all$SK, "FN", "FY", "FX", "MN", "XN", "XX")
df_all$State <- as.integer(df_all$SK) # integer version of animal state

df_all <- arrange(df_all, DateObs, SK, Lat)
```

```r
# generate a map of the observations
us_can <- c(left = -155, bottom = 36, right = -101, top = 65)
# the code below requires an internet connection
p_1A <- get_stamenmap(us_can, zoom = 4, maptype = "toner-lite") %>%
  ggmap() +
  annotate("point", x = -135.338283, y = 60.871533, size = 5, shape = 4) +
  geom_point(data = df_all, aes(x = Long, y = Lat, color = SK),
    inherit.aes = FALSE) +
  scale_colour_manual(values=c("#980043", "#e7298a", "#c994c7",
    "blue", "black", "grey50")) +
  labs(color = "Animal\nstate", x = "Longitude", y = "Latitude") +
  guides(color=FALSE) +
  theme_bw()

p_1B <- ggplot(df_all, aes(x = Lat, y = ElevGIS, color = SK)) +
  geom_point() +
  scale_colour_manual(values=c("#980043", "#e7298a", "#c994c7",
    "blue", "black", "grey50")) +
  labs(x = "Latitude", y = "Elevation (m)", color = "Animal\nstate") +
  theme_bw()

plot_grid(p_1A, p_1B, ncol = 2, rel_widths = c(1,1), labels = c("A", "B"))
```

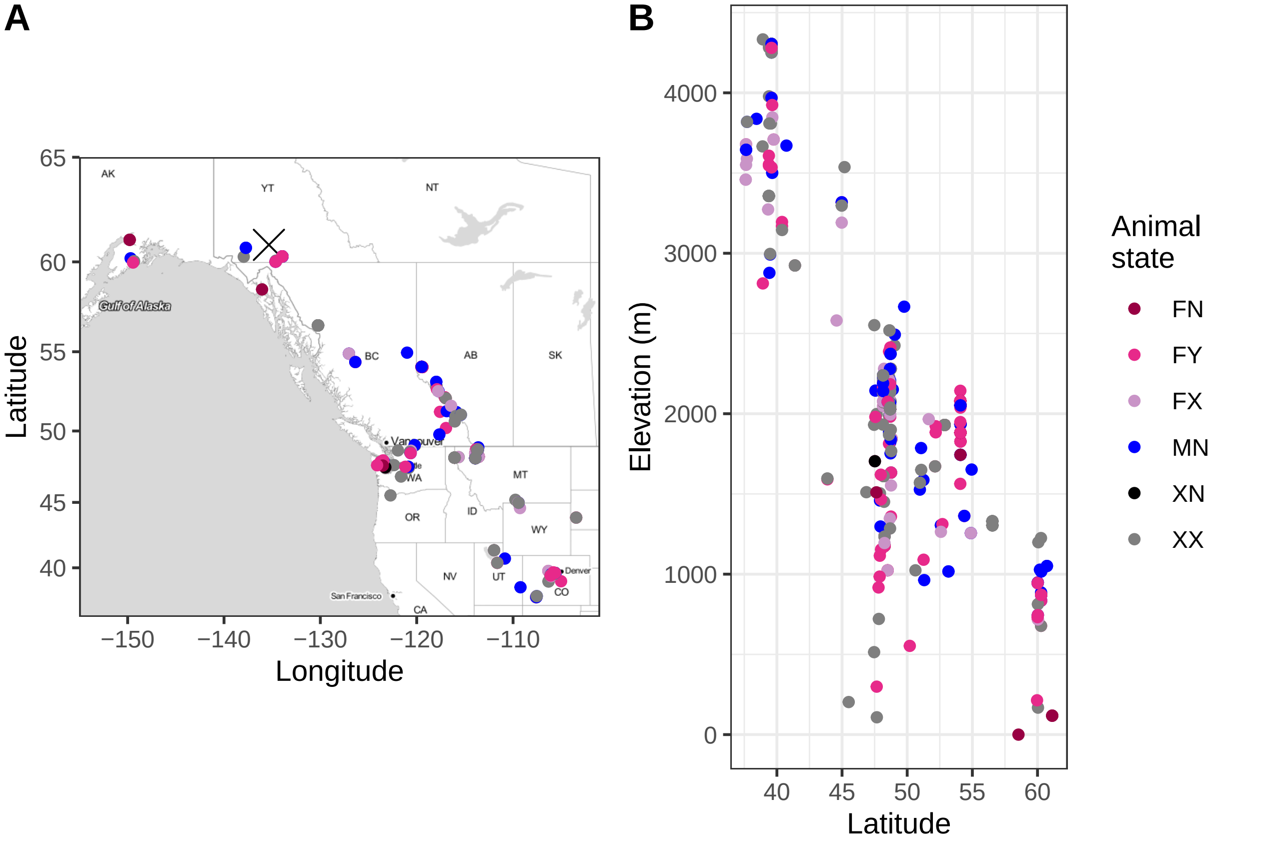

Figure 1: (A) Locations of photographs analyzed. Cross indicates the location of the Yukon Wildlife Preserve. (B) Relation between latitude and elevation for all photographs. Colours show what is known about the sex and presence of a kid for each animal photographed. There are six possible animal states described by a pair of letters: [first pair] F = female, M = male, X = sex unknown; [second pair] Y = with

kid, N = without kid, X = kid status unknown.

The six animal states are:

- FN: Female without kid
- FY: Female with kid
- FX: Female but presence of kid is unknown
- MN: Male without kid
- XN: Sex unknown and without kid
- XX: Sex unknown and presence of kid is unknown

Shedding fractions

Let's plot the data by year but in each panel include all data as grey dots to look for evidence of annual differences.

```
# yearly sample sizes
df_years <- df_all %>%
  group_by(year) %>%
  summarise(
    n_all = n(), # annual sample sizes
    .groups = 'drop'
  )

# yearly sample sizes per animal state
df_states <- df_all %>%
    filter(SK %in% c("FX", "XN", "XX")) %>%
  group_by(year) %>%
  summarise(
    n_unknown = n(), # sample sizes
    .groups = 'drop'
  )

# calculate proportion per state
df_years <- left_join(df_years, df_states, by = "year")

df_years$n_unknown[which(is.na(df_years$n_unknown))] <- 0

df_years <- df_years %>%
    mutate(txt = paste(n_all, "(", n_unknown, ")", sep = ""))

# create data frames for making an informative plot
#   of the shedding observations
df_plot <- df_all
# need a new data frame for the background dots
df_grey <- df_all %>%
  select(doy, frac_shed, SK)
```

```
ggplot() +
  geom_point(data = df_grey, mapping = aes(x = doy, y = frac_shed),
    color = "grey90", inherit.aes = FALSE) +
  geom_point(data = df_plot,
```

```
    mapping = aes(x = doy, y = frac_shed, color = SK)) +
    geom_text(data = df_years, size = 3,
        mapping = aes(x = 330, y = 0.3, label = txt), hjust = 1) +
  scale_colour_manual(values=c("#980043", "#e7298a", "#c994c7",
    "blue", "black", "grey50")) +
  labs(
    x = "Day of year",
    y = "Fraction shed",
    color = "Animal state") +
    guides(color = guide_legend(nrow = 3)) +
  facet_wrap( ~ year, ncol = 6) +
  theme_bw() +
  theme(
    axis.text.x = element_text(angle=60, hjust=1),
    legend.position=c(1,0),
    legend.justification=c(1,0),
    legend.text = element_text(size=7),
    panel.grid = element_blank()
  )
```

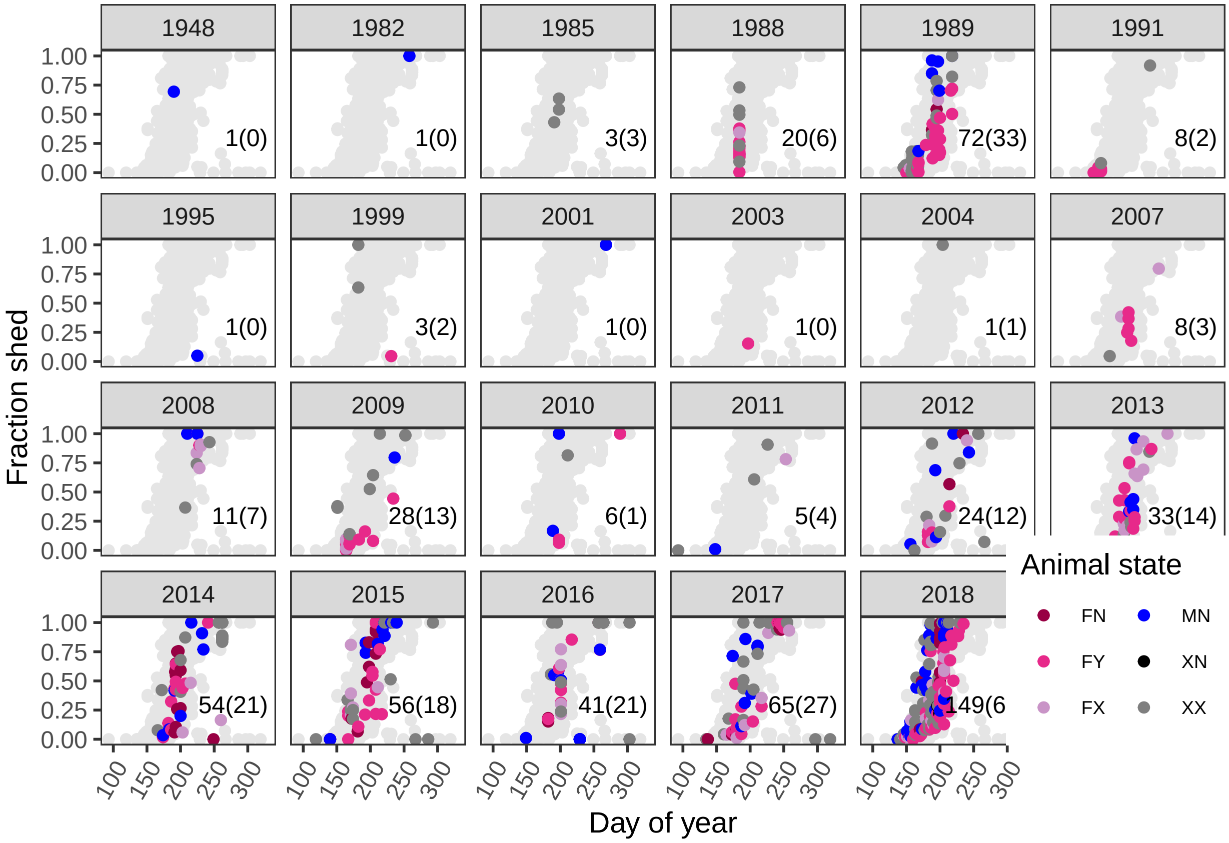

Figure 2: Observed shedding fractions throughout the year. Numbers depict sample sizes (i.e. photographs) and and bracketed values depict the number of photographs where the state of the animal is ambiguous. Grey points show observations for all years without the information on animal state, whereas coloured circles depict observations recorded that year and animal state.

Data are very sparse in the earlier years and many photographs are ambiguous regarding animal state (Figure 2). Males are molting earlier and females with kid are molting later (Figure 2), which is consistent with

findings from data collected in the Yukon Wildlife Preserve (YWP) (see separate data set). There is no obvious long-term trend in the onset of molting.

Environmental predictors

Now we will investigate the the spatial distribution of the photographs across years.

```
df_grey <- df_all %>%
  select(Lat, Long)

ggplot() +
  geom_point(data = df_grey, mapping = aes(x = Long, y = Lat),
    color = "grey90", inherit.aes = FALSE) +
  geom_point(data = df_plot,
    mapping = aes(x = Long, y = Lat, color = SK)) +
    geom_text(data = df_years, size = 3,
        mapping = aes(x = -150, y = 45, label = txt), hjust = 0) +
  scale_colour_manual(values=c("#980043", "#e7298a", "#c994c7",
    "blue", "black", "grey50")) +
  labs(x = "Longitude", y = "Latitude", color = "Animal\nstate") +
  facet_wrap( ~ year, ncol = 5) +
  theme_bw() +
  theme(panel.grid = element_blank())
```

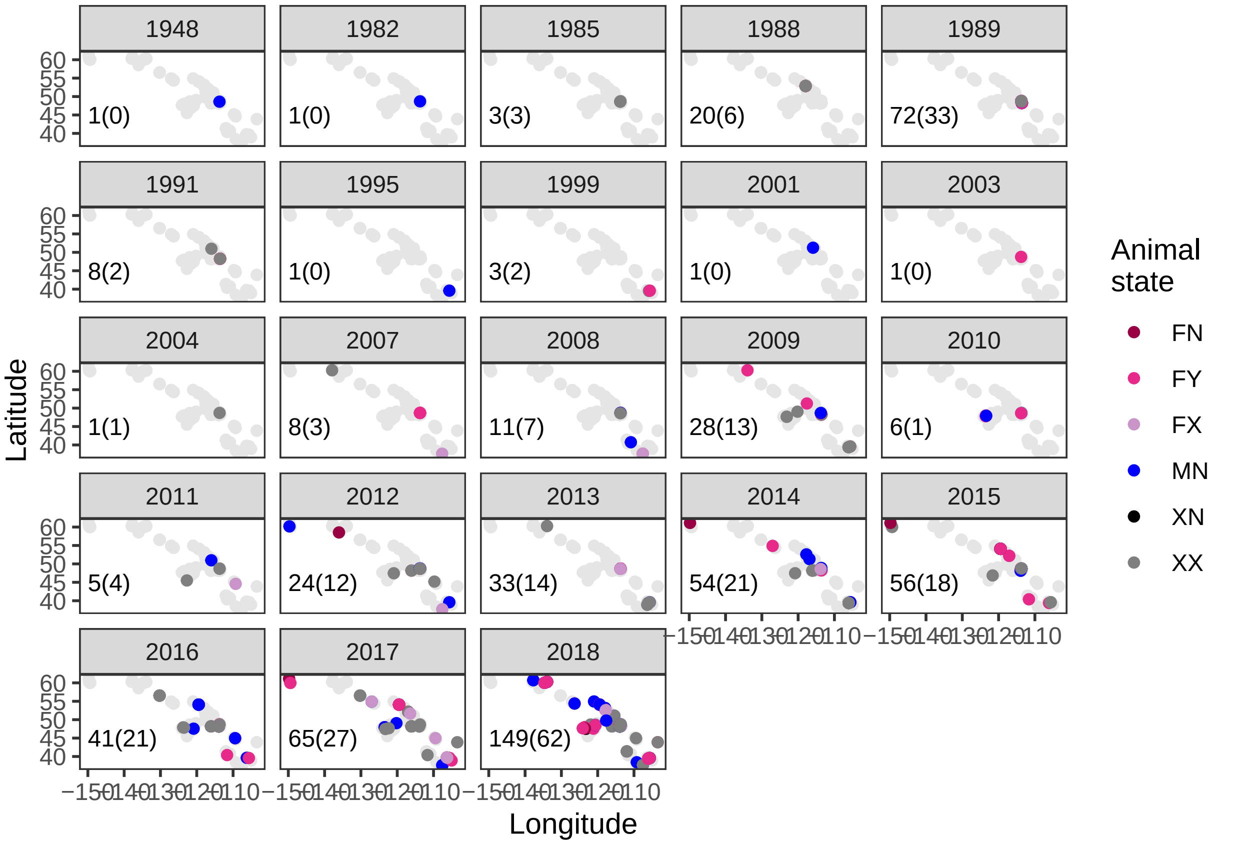

Figure 3: Locations of photographs throughout the citizen science study. Grey points show all observations and coloured circles depict observations recorded that year and animal state (see Figure 2 legend for more

details). Numbers depict the abundance of photographs and the brackets indicate how many are associated with an ambiguous state.

The data set covers a wide latitudinal range, and elevation and latitude are negatively correlated (Figure 1).

```
cor.test(df_all$Lat, df_all$ElevGIS) # calculate the correlation
```

```
##
##  Pearson's product-moment correlation
##
## data:  df_all$Lat and df_all$ElevGIS
## t = -30.553, df = 590, p-value < 2.2e-16
## alternative hypothesis: true correlation is not equal to 0
## 95 percent confidence interval:
##  -0.8121309 -0.7494674
## sample estimates:
##        cor
## -0.7827755
```

Years associated with relatively high numbers of photographs tend to have high spatial coverage, except for 1988 and 1989 (Figure 3).

Inconsistent data

There appears to be some outliers; for example, a number of shedding fractions recorded late in the year are unusually low (Figure 2). Here, we define an outlier as an animal that has shed < 20% of its coat after day 225.

```
N_init <- nrow(df_all) # initial number of obs

# filter out observations that look like data entry errors
df_all <- df_all %>%
  mutate(
    wrong_shed = (doy >= 225) & frac_shed < 0.2
  ) # < 20% shed after day 225

df_grey <- df_all %>%
  select(doy, frac_shed, SK)

df_fit <- df_all %>%
  filter(wrong_shed == FALSE) # remove apparent outliers

ggplot(df_all, aes(x = doy, y = frac_shed, color = wrong_shed)) +
  geom_point() +
  scale_colour_manual(values=c("grey", "black")) +
  labs(
    x = "Day of year",
    y = "Fraction shed",
    color = "Unusual\nmolting\nfraction"
  ) +
  theme_bw()
```

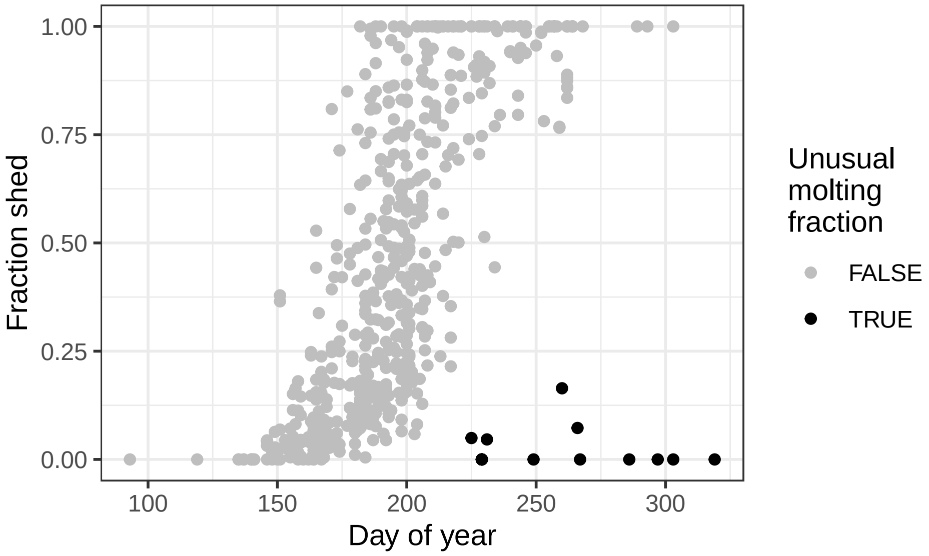

Figure 4: Observations considered to have a reliable date and shed fraction (grey) and those considered to be inconsistent with the overall distribution (black). Only the grey data will be included in the model fit to prevent bias parameter estimates.

Only 15 observations (2.53%) were classified as outliers.

Predictor variables

What about correlation among the predictor variables? And, are the inconsistent data associated with any of the predictors?

```
# check for correlations among predictors
df_all %>%
  select(
    Latitude = Lat,
    Elevation = ElevGIS,
    Year = year,
    State = State,
    "Unreliable" = wrong_shed
  ) %>%
  ggpairs(. ,
    mapping = ggplot2::aes(color = Unreliable),
    lower = list(combo = wrap("facethist", bins = 20)))
```

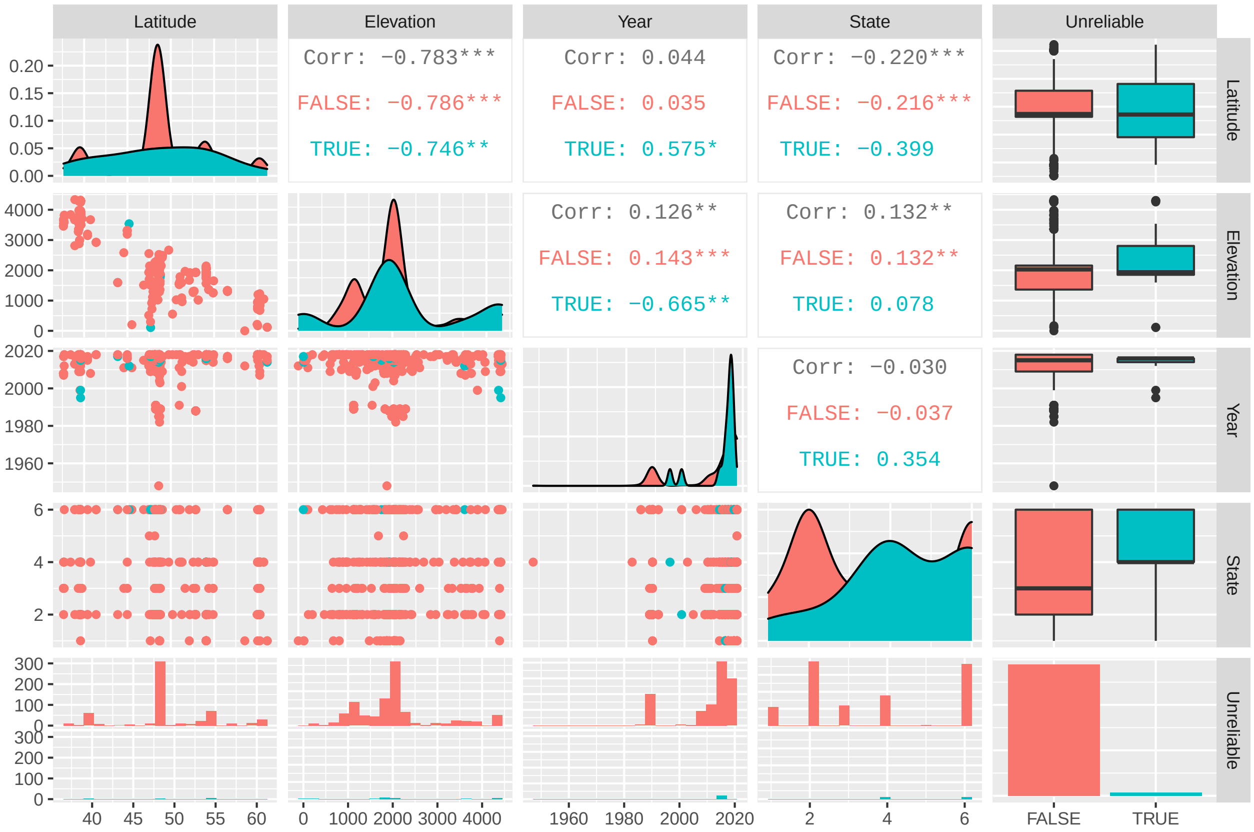

Figure 5: Relations among predictor variables. Inconsistent data are those where molting fraction late in the year is low (Figure 4). Animal states 1-6 represent: FN, FY, FX, MN, XN, XX.

There are no clear conditions associated with the unreliable shedding fractions. # Uncertainty in animal state

Here we look for evidence of consistency across years of the fraction of states that are classified as unambiguous and ambiguous. This is an important assumption of the statistical model that incorporates incomplete information. First, look at the data where sex and presence of kid is known.

```
# yearly sample sizes
df_years <- df_fit %>%
    filter(SK %in% c("FN", "FY", "MN")) %>%
  group_by(year) %>%
  summarise(
    n_year = n(), # annual sample sizes
    .groups = 'drop'
  )

# yearly sample sizes per animal state
df_states <- df_fit %>%
    filter(SK %in% c("FN", "FY", "MN")) %>%
  group_by(year,SK) %>%
  summarise(
    n_state = n(), # sample sizes
    .groups = 'drop'
  )

# calculate proportion per state
```

```r
df_states <- left_join(df_states, df_years, by = "year") %>%
    mutate(f =  n_state / n_year)

# filter out poorly sampled years
df_years  <- filter(df_years, n_year >= 10)
df_states <- filter(df_states, n_year >= 10)
```

```r
ggplot() +
      geom_hline(yintercept = 0.33, color = "blue") +
        geom_bar(data = df_states, position="stack", stat="identity",
            mapping = aes(x = factor(year), y = f, fill = SK)) +
      geom_text(data = df_years, color = "white",
        mapping = aes(x = factor(year), y = 0.05, label = n_year)) +
    scale_fill_manual(values=c("#980043", "#e7298a", "blue")) +
      geom_hline(yintercept = 0.33, color = "blue") +
      geom_hline(yintercept = 0.33 + 0.5*0.67, color = "#980043") +
      labs(
        x = "Year",
        y = "Fraction of photographs",
        fill = "State") +
        theme_bw()
```

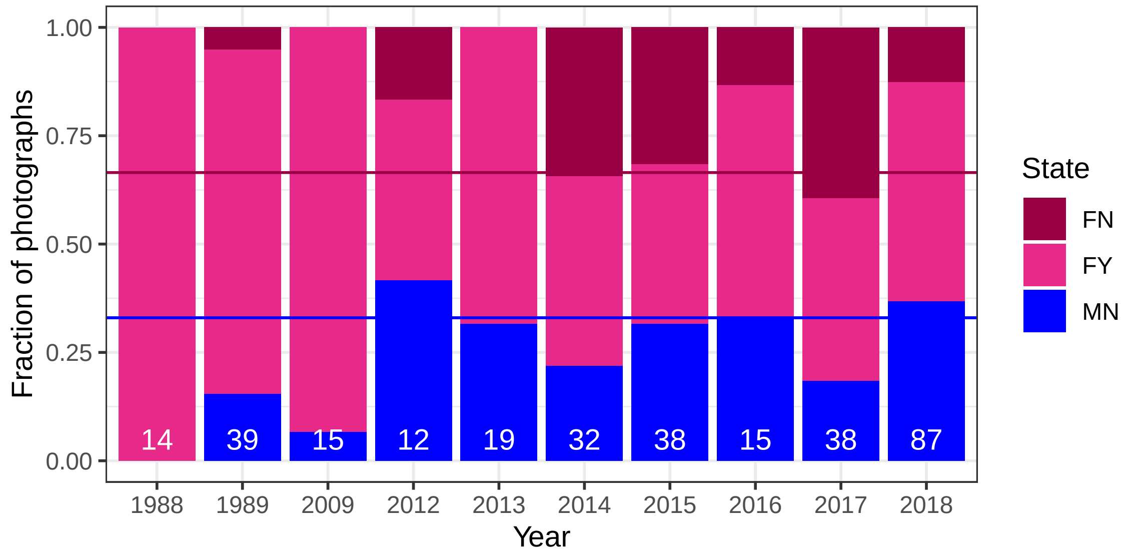

Figure 6: Fraction of photographs with known animal state across years. Horizontal lines depict the predicted proportions of known states according to parameters estimated by the model (see later). White numbers depict the number of photographs; only years where ten or more photos were taken are depicted.

There is reasonable consistency among years in these three proportions, and consistency will be an important model assumption.

What about the proportions of uncertain states across years?

```r
# yearly sample sizes
df_years <- df_fit %>%
    filter(SK %in% c("FX", "XN", "XX")) %>%
  group_by(year) %>%
  summarise(
```

```r
    n_year = n(), # annual sample sizes
    .groups = 'drop'
  )

# yearly sample sizes per animal state
df_states <- df_fit %>%
    filter(SK %in% c("FX", "XN", "XX")) %>%
  group_by(year,SK) %>%
  summarise(
    n_state = n(), # sample sizes
    .groups = 'drop'
  )

# calculate proportion per state
df_states <- left_join(df_states, df_years, by = "year") %>%
    mutate(f =  n_state / n_year) %>%
    filter(n_year >= 10)

df_years <- filter(df_years, n_year >= 10)
```

```r
ggplot() +
        geom_bar(data = df_states, position="stack", stat="identity",
            mapping = aes(x = factor(year), y = f, fill = SK)) +
      geom_text(data = df_years,
        mapping = aes(x = factor(year), y = 0.05, label = n_year)) +
    scale_fill_manual(values=c("#c994c7", "black", "grey")) +
      labs(
        x = "Year",
        y = "Proportion",
        fill = "State") +
        theme_bw()
```

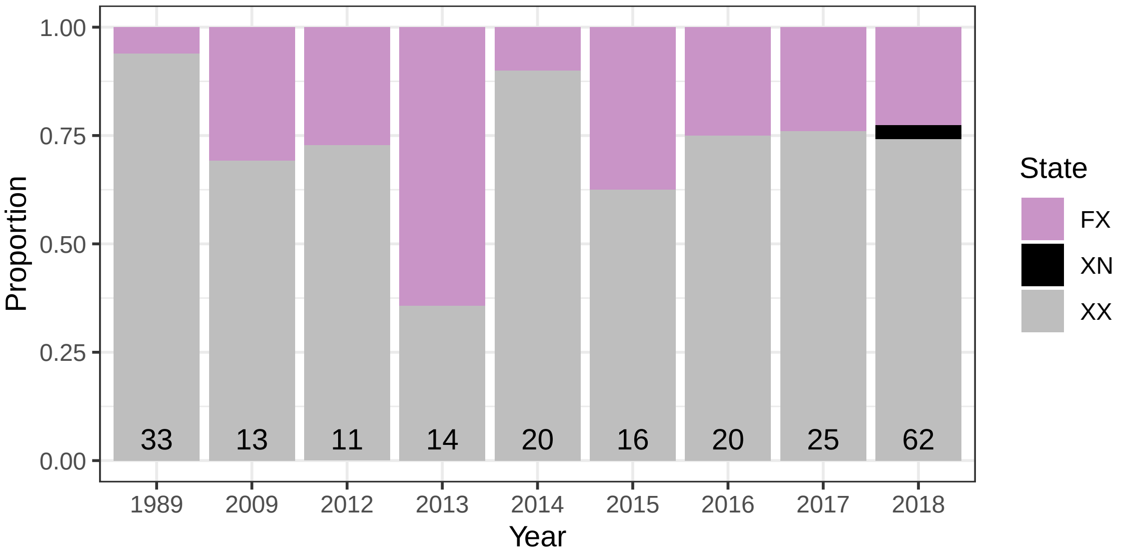

Figure 7: Fraction of photographs associated with some ambiguity regarding animal state.

Again, there is consistency in these proportions across years. Now, let's look at all states.

```r
# yearly sample sizes
df_years <- df_fit %>%
  group_by(year) %>%
  dplyr::summarise(
    n_year = n(), # annual sample sizes
    .groups = 'drop'
  )

# yearly sample sizes per animal state
df_states <- df_fit %>%
  group_by(year,SK) %>%
  dplyr::summarise(
    n_state = n(), # sample sizes
    .groups = 'drop'
  )

# calculate proportion per state
df_states <- left_join(df_states, df_years, by = "year") %>%
    mutate(f =  n_state / n_year) %>%
    filter(n_year >= 20)

df_years <- filter(df_years, n_year >= 20)
```

```r
ggplot() +
        geom_bar(data = df_states, position="stack", stat="identity",
            mapping = aes(x = factor(year), y = f, fill = SK)) +
      geom_text(data = df_years,
        mapping = aes(x = factor(year), y = 0.05, label = n_year)) +
    scale_fill_manual(values=c("#980043", "#e7298a", "#c994c7",
      "blue", "black", "grey50")) +
      labs(
        x = "Year",
        y = "Proportion",
        fill = "State") +
        theme_bw()
```

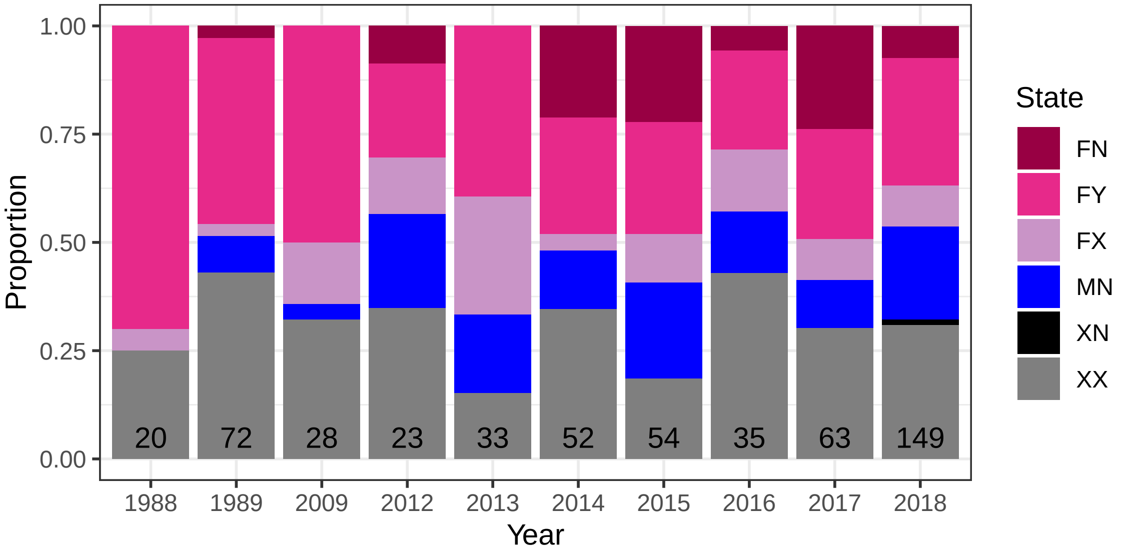

Figure 8: Fraction of photographs associated with each of the six possible animal states.

With six states and some years with low sample sizes we would expect some variation in the proportions for each state; nonetheless, the proportions are relatively consistent across years.

In conclusion, our assumption of relatively consistent fractions of animal states across years seems reasonable.

Fitted data set

A common property of ecological data is inter-annual variation in the timing of life-history events. The statistical model will incorporate inter-annual variation by employing a random factor, using year as the levels of the factor. Specifically, each year will be associated with a random offset, drawn from a t-distribution with mean zero, that describes the shift in 50% shedding date from the long-term mean. In order to estimate the offset for any given year requires sufficient data (i.e. information). In this study we have photographs associated with ambiguity, which provide little information individually. Here, we will only consider years where we have at least one photograph without ambiguity on animal state.

```
# yearly sample sizes
df_years <- df_fit %>%
  group_by(year) %>%
  summarise(
    n_all = n() # annual sample sizes
  )

# yearly sample sizes per animal state
df_states <- df_fit %>%
    filter(SK %in% c("FX", "XN", "XX")) %>%
  group_by(year) %>%
  summarise(
    n_unknown = n() # sample sizes
  )

# calculate proportion per state
df_years <- left_join(df_years, df_states, by = "year")

df_years$n_unknown[which(is.na(df_years$n_unknown))] <- 0

df_years <- df_years %>%
    mutate(
        n_known = n_all - n_unknown,
        txt = paste(n_all, "(", n_unknown, ")", sep = "")
    )

# determine the years to include in the fit
# must have at least one known state
ok_years <- df_years$year[which(df_years$n_known > 1)]

df_fit <- filter(df_fit, year %in% ok_years)
df_years <- filter(df_years, year %in% ok_years)

# save as PDF portrait: 8 by 5
df_grey <- df_fit %>%
  select(doy, frac_shed, SK)
```

```
ggplot() +
  geom_point(data = df_grey, aes(x = doy, y = frac_shed),
    color = "grey90", inherit.aes = FALSE) +
  geom_point(data = df_fit,
    mapping = aes(x = doy, y = frac_shed, color = SK)) +
    geom_text(data = df_years, size = 3,
        mapping = aes(x = 330, y = 0.3, label = txt), hjust = 1) +
    scale_colour_manual(values=c("#980043", "#e7298a", "#c994c7",
    "blue", "black", "grey50")) +
  labs(
    x = "Day of year",
    y = "Fraction shed",
    color = "Animal state"
  ) +
    guides(color = guide_legend(nrow = 3)) +
  facet_wrap( ~ year, ncol = 5) +
  theme_bw() +
  theme(
    axis.text.x = element_text(angle=60, hjust=1),
    legend.position=c(1,0),
    legend.justification=c(1,0),
    legend.text = element_text(),
    panel.grid = element_blank()
  )
```

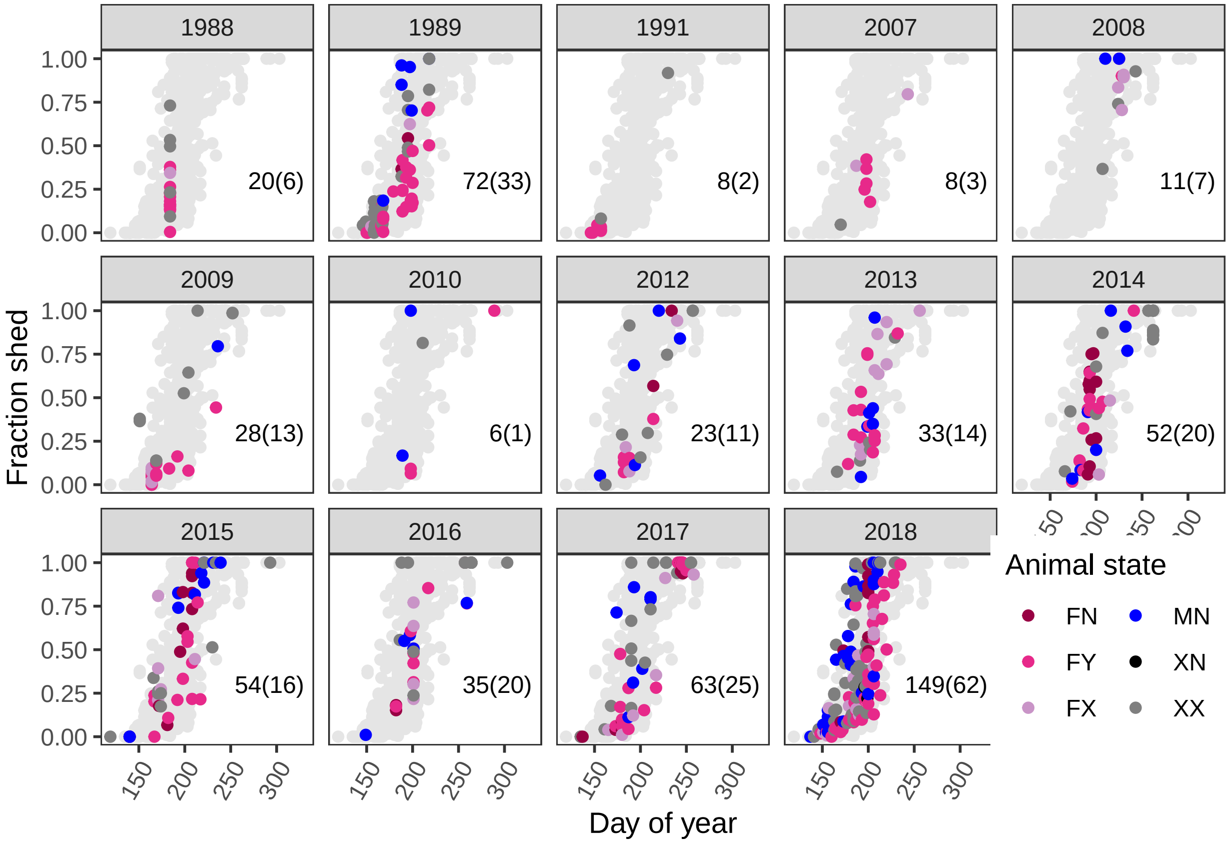

Figure 9: Observed shedding fractions used when fitting the statistical models. Numbers depict sample sizes (i.e. photographs) and and bracketed values depict the number of photographs where the state of the

animal is ambiguous. Grey points show observations for all years without the information on animal state, whereas coloured circles depict observations recorded that year and animal state.

```
# save the final data set for analysis
write_csv(select(df_fit, -wrong_shed), path = "CitizenScienceWrangled.csv")
```
