# Supplementary material for: Using community photography to investigate phenology: A case study of coat molt in the mountain goat (Oreamnos americanus) with missing data
Source: Ecol Evol. 2020 Nov 9;10(23):13488–99. doi: 10.1002/ece3.6954 (PMC7713987; doi:10.1002/ece3.6954)
Supplement: Supplementary file 2 — Supinfo2 [file ECE3-10-13488-s002.pdf]

Supplementary Materials 2: Community-Sourced Data Analysis

Shane A. Richards

2020-09-13

Contents

Summary 1

Model of molting 2

Data 3

Model Fit 4

Chain convergence 14

Parameter correlations 15

Parameter estimates 17

Parameter Credibility 18

Random effects 19

Predictions 21

Long-term trend 21

Within-season 23

Stan code 26

Summary

This document supports the manuscript:

> Nowak et al. "Using community photography to investigate phenology: a case study of coat molt in the mountain goat (Oreamnos americanus) with missing data" submitted to Ecology and Evolution.

Here we present the statistical analysis of the community-sourced data set.

Model of molting

A summary of the model is presented here. Full details of the model can be found in the manuscript.

Goats may be in one of three states: FN (female without a kid), FY (female with a kid), or MN (male without a kid). At any time during periods of observation it is assumed that proportion p of photographed animals are female and proportion q of the females are associated with a kid. Thus, the average proportion of animals in states: FN, FY, and MN, are $p(1-q)$, pq, and $(1-p)$, respectively.

Let $f(t)$ denote the mean fraction of coat shed at time t:

$$f(t) = \frac{e^{\alpha(t-\tau)}}{1 + e^{\alpha(t-\tau)}}$$

Here, we assume $0 < t < 1$ so that the bounds correspond to January 1 and December 31. τ $(0 < \tau < 1)$ is the day of year when females have shed half of their coat.

Model fitting is helped by categorising the molting fraction into N equal-sized bins, so that the response variable, n is the number of shed bins $(0 \leq n \leq N)$. Our statistical findings are robust to the choice of N. The probability of observing n bins shed when the expected fraction is f, is given by the beta-binomial distribution. The beta-component accounts for over-dispersion in the observations relative to the binomial distribution.

Animals may be classified into three additional states if gender or presence of kid is unknown. Let X denote the source of uncertainty, giving three uncertain states: FX, XN, XX. We assume that all animals associated with a kid are female. For these three states the probability of an observation is the weighted sum of the three beta-binomial distributions, where the weights are calculated using p and q. The probability of observing datum y for each of these states are:

$$\Pr(n|\text{FX}) = (1-q)P_{\text{BB}}(n|\text{FN}) + qP_{\text{BB}}(n|\text{FY}),$$

$$\Pr(n|\text{XN}) = \frac{p(1-q)P_{\text{BB}}(n|\text{FN}) + (1-p)P_{\text{BB}}(n|\text{MN})}{p(1-q) + (1-p)},$$

and

$$\Pr(n|\text{XX}) = p(1-q)P_{\text{BB}}(n|\text{FN}) + pqP_{\text{BB}}(n|\text{FY}) + (1-p)P_{\text{BB}}(n|\text{MN}),$$

where $P_{\text{BB}}(n|i)$ is the probability of observing n successes given a beta-binomial distribution with N trials. The mean probability of trial success is f, which will depend ion the state of the animal, i, and the environmental variables. The variance parameter, ϕ, sets the variance of the observations to be $\text{Var} = vNf(1-f)$, where $v = 1 + (N-1)\phi/(1+\phi)$ is the variance inflation factor.

Covariates that may affect the timing of molting (e.g. elevation, latitude, and year) are incorporated into the model by letting them affect τ and α. Timing may also be affected by whether the animal is male, $(x_{\text{M}} = 0, 1)$, or has a kid $(x_{\text{K}} = 0, 1)$. In addition, we allow random year-year variation in the timing of molting. If x_{E}, x_{L}, and x_{Y}, denote the elevation, latitude and year of the observation, then

$$\tau = \tau_0 + \sum_{i \in \{\text{M,K,E,L,Y}\}} \tau_i x_i + \tau_{[y]},$$

where $\tau_{[y]} \sim \text{N}(0, \sigma_\tau)$ is the random offset associated for year, y. Thus, τ_{Y} and the $\delta_{\tau,y}$ describe long-term and short-term (inter-annual) variation in the timing of molting.

Similarly, the environmental covariates (but not animal state) may affect the rate of molting such that:

$$\alpha = \alpha_0 + \sum_{i \in \{\text{M,K,E,L,Y}\}} \alpha_i x_i + \alpha_{[y]},$$

where $\alpha_{[y]} \sim \text{N}(0, \sigma_\alpha)$ is the random rate offset associated for year, y.

To help with model fitting we z-transform the environmental predictor variables, $X_i, i \in \{\text{E, L, Y}\}$.

Data

First, we read in the wrangled data (see Supplementary Materials 1) and then set up the data for feeding into the model fitting using stan.

```
rm(list = ls()) # clear memory

# load all packages needed for the analysis (quite a few!)
library(tidyverse)
library(readxl)
library(lubridate)
library(rstan)
library(cowplot)
library(scales)
library(ggmap)
library(bayesplot)

# read in the data
df_fit <- read_csv("../Data Wrangling/CitizenScienceWrangled.csv")

SHED <- 25 # bins that define molting state (25 or 100)

# add binned shedding data
df_fit <- df_fit %>%
  mutate(shed = as.integer(round(SHED*frac_shed, 0)))

# create appropriate factors describing animal state
df_fit$Sex  <- factor(df_fit$Sex)
df_fit$Sex  <- fct_relevel(df_fit$Sex,  "F", "M", "X")
df_fit$Kids <- factor(df_fit$Kids)
df_fit$Kids <- fct_relevel(df_fit$Kids, "N", "Y", "X")
df_fit$SK   <- factor(df_fit$SK)
df_fit$SK   <- fct_relevel(df_fit$SK, "FN", "FY", "FX", "MN", "XN", "XX")

df_fit <- arrange(df_fit, DateObs, SK, Lat)

# prepare data for fitting with stan (i.e. z-transformations of predictors)
Lat_mu <- mean(df_fit$Lat)
Lat_sd <- sd(df_fit$Lat)

Elv_mu <- mean(df_fit$ElevGIS)
Elv_sd <- sd(df_fit$ElevGIS)

doy_mu <- mean(df_fit$doy)
doy_sd <- sd(df_fit$doy)

yr_mu <- mean(df_fit$year)
yr_sd <- sd(df_fit$year)

df_fit$yr_F  <- factor(df_fit$year) # create a factor version of year
df_fit$fYear <- as.integer(factor(df_fit$year))

# z-transform predictors and set 0 < DOY < 1
df_fit$z_yr  <- (df_fit$year - yr_mu) / yr_sd
```

```r
df_fit$z_doy  <- df_fit$doy / 365
df_fit$z_lat <- (df_fit$Lat - Lat_mu) / Lat_sd
df_fit$z_ele <- (df_fit$ElevGIS - Elv_mu) / Elv_sd

# data in a form for model fitting
stan_dat <- list(
  I     = nrow(df_fit), # number of observations
  N     = SHED,         # molting categories
  Y     = max(df_fit$fYear), # number of years
  State = df_fit$State, # animal state (1-6)
  zt    = df_fit$z_doy, # transformed day of year
  ze    = df_fit$z_ele, # z-transformed elevation
  zl    = df_fit$z_lat, # z-transformed latitude
  zy    = df_fit$z_yr,  # z-transformed year
  iy    = df_fit$fYear, # year index
  n     = df_fit$shed   # observed shed (0-N)
)
```

Model Fit

```r
# fit the model!
fit <- stan(file = 'CSfit.stan', data = stan_dat,
  iter = 2000, warmup = 1000, chains = 3, seed = 1971) # , refresh = 0)
```

```
## Warning in readLines(file, warn = TRUE): incomplete final line found on '/Users/
## shaner2/Documents/UTAS/Research/Projects/Mountain goat/Analysis/EvoEcolRevision/
## CitSci Complete/CSfit.stan'
```

```
##
## SAMPLING FOR MODEL 'CSfit' NOW (CHAIN 1).
## Chain 1: Rejecting initial value:
## Chain 1:   Log probability evaluates to log(0), i.e. negative infinity.
## Chain 1:   Stan can't start sampling from this initial value.
## Chain 1: Rejecting initial value:
## Chain 1:   Log probability evaluates to log(0), i.e. negative infinity.
## Chain 1:   Stan can't start sampling from this initial value.
## Chain 1: Rejecting initial value:
## Chain 1:   Log probability evaluates to log(0), i.e. negative infinity.
## Chain 1:   Stan can't start sampling from this initial value.
## Chain 1: Rejecting initial value:
## Chain 1:   Log probability evaluates to log(0), i.e. negative infinity.
## Chain 1:   Stan can't start sampling from this initial value.
## Chain 1: Rejecting initial value:
## Chain 1:   Log probability evaluates to log(0), i.e. negative infinity.
## Chain 1:   Stan can't start sampling from this initial value.
## Chain 1: Rejecting initial value:
## Chain 1:   Log probability evaluates to log(0), i.e. negative infinity.
## Chain 1:   Stan can't start sampling from this initial value.
## Chain 1: Rejecting initial value:
## Chain 1:   Log probability evaluates to log(0), i.e. negative infinity.
```

```
## Chain 1:   Stan can't start sampling from this initial value.
## Chain 1: Rejecting initial value:
## Chain 1:   Log probability evaluates to log(0), i.e. negative infinity.
## Chain 1:   Stan can't start sampling from this initial value.
## Chain 1: Rejecting initial value:
## Chain 1:   Log probability evaluates to log(0), i.e. negative infinity.
## Chain 1:   Stan can't start sampling from this initial value.
## Chain 1: Rejecting initial value:
## Chain 1:   Log probability evaluates to log(0), i.e. negative infinity.
## Chain 1:   Stan can't start sampling from this initial value.
## Chain 1: Rejecting initial value:
## Chain 1:   Log probability evaluates to log(0), i.e. negative infinity.
## Chain 1:   Stan can't start sampling from this initial value.
## Chain 1: Rejecting initial value:
## Chain 1:   Log probability evaluates to log(0), i.e. negative infinity.
## Chain 1:   Stan can't start sampling from this initial value.
## Chain 1: Rejecting initial value:
## Chain 1:   Log probability evaluates to log(0), i.e. negative infinity.
## Chain 1:   Stan can't start sampling from this initial value.
## Chain 1: Rejecting initial value:
## Chain 1:   Log probability evaluates to log(0), i.e. negative infinity.
## Chain 1:   Stan can't start sampling from this initial value.
## Chain 1: Rejecting initial value:
## Chain 1:   Log probability evaluates to log(0), i.e. negative infinity.
## Chain 1:   Stan can't start sampling from this initial value.
## Chain 1: Rejecting initial value:
## Chain 1:   Log probability evaluates to log(0), i.e. negative infinity.
## Chain 1:   Stan can't start sampling from this initial value.
## Chain 1: Rejecting initial value:
## Chain 1:   Log probability evaluates to log(0), i.e. negative infinity.
## Chain 1:   Stan can't start sampling from this initial value.
## Chain 1: Rejecting initial value:
## Chain 1:   Log probability evaluates to log(0), i.e. negative infinity.
## Chain 1:   Stan can't start sampling from this initial value.
## Chain 1: Rejecting initial value:
## Chain 1:   Log probability evaluates to log(0), i.e. negative infinity.
## Chain 1:   Stan can't start sampling from this initial value.
## Chain 1: Rejecting initial value:
## Chain 1:   Log probability evaluates to log(0), i.e. negative infinity.
## Chain 1:   Stan can't start sampling from this initial value.
## Chain 1: Rejecting initial value:
## Chain 1:   Log probability evaluates to log(0), i.e. negative infinity.
## Chain 1:   Stan can't start sampling from this initial value.
## Chain 1: Rejecting initial value:
## Chain 1:   Log probability evaluates to log(0), i.e. negative infinity.
## Chain 1:   Stan can't start sampling from this initial value.
## Chain 1: Rejecting initial value:
## Chain 1:   Log probability evaluates to log(0), i.e. negative infinity.
## Chain 1:   Stan can't start sampling from this initial value.
## Chain 1: Rejecting initial value:
## Chain 1:   Log probability evaluates to log(0), i.e. negative infinity.
## Chain 1:   Stan can't start sampling from this initial value.
## Chain 1: Rejecting initial value:
## Chain 1:   Log probability evaluates to log(0), i.e. negative infinity.
```

```
## Chain 1:   Stan can't start sampling from this initial value.
## Chain 1: Rejecting initial value:
## Chain 1:   Log probability evaluates to log(0), i.e. negative infinity.
## Chain 1:   Stan can't start sampling from this initial value.
## Chain 1: Rejecting initial value:
## Chain 1:   Log probability evaluates to log(0), i.e. negative infinity.
## Chain 1:   Stan can't start sampling from this initial value.
## Chain 1: Rejecting initial value:
## Chain 1:   Log probability evaluates to log(0), i.e. negative infinity.
## Chain 1:   Stan can't start sampling from this initial value.
## Chain 1: Rejecting initial value:
## Chain 1:   Log probability evaluates to log(0), i.e. negative infinity.
## Chain 1:   Stan can't start sampling from this initial value.
## Chain 1: Rejecting initial value:
## Chain 1:   Log probability evaluates to log(0), i.e. negative infinity.
## Chain 1:   Stan can't start sampling from this initial value.
## Chain 1: Rejecting initial value:
## Chain 1:   Log probability evaluates to log(0), i.e. negative infinity.
## Chain 1:   Stan can't start sampling from this initial value.
## Chain 1: Rejecting initial value:
## Chain 1:   Log probability evaluates to log(0), i.e. negative infinity.
## Chain 1:   Stan can't start sampling from this initial value.
## Chain 1: Rejecting initial value:
## Chain 1:   Log probability evaluates to log(0), i.e. negative infinity.
## Chain 1:   Stan can't start sampling from this initial value.
## Chain 1: Rejecting initial value:
## Chain 1:   Log probability evaluates to log(0), i.e. negative infinity.
## Chain 1:   Stan can't start sampling from this initial value.
## Chain 1: Rejecting initial value:
## Chain 1:   Log probability evaluates to log(0), i.e. negative infinity.
## Chain 1:   Stan can't start sampling from this initial value.
## Chain 1: Rejecting initial value:
## Chain 1:   Log probability evaluates to log(0), i.e. negative infinity.
## Chain 1:   Stan can't start sampling from this initial value.
## Chain 1: Rejecting initial value:
## Chain 1:   Log probability evaluates to log(0), i.e. negative infinity.
## Chain 1:   Stan can't start sampling from this initial value.
## Chain 1: Rejecting initial value:
## Chain 1:   Log probability evaluates to log(0), i.e. negative infinity.
## Chain 1:   Stan can't start sampling from this initial value.
## Chain 1: Rejecting initial value:
## Chain 1:   Log probability evaluates to log(0), i.e. negative infinity.
## Chain 1:   Stan can't start sampling from this initial value.
## Chain 1: Rejecting initial value:
## Chain 1:   Log probability evaluates to log(0), i.e. negative infinity.
## Chain 1:   Stan can't start sampling from this initial value.
## Chain 1: Rejecting initial value:
## Chain 1:   Log probability evaluates to log(0), i.e. negative infinity.
## Chain 1:   Stan can't start sampling from this initial value.
## Chain 1: Rejecting initial value:
## Chain 1:   Log probability evaluates to log(0), i.e. negative infinity.
```

```
## Chain 1:   Stan can't start sampling from this initial value.
## Chain 1: Rejecting initial value:
## Chain 1:   Log probability evaluates to log(0), i.e. negative infinity.
## Chain 1:   Stan can't start sampling from this initial value.
## Chain 1: Rejecting initial value:
## Chain 1:   Log probability evaluates to log(0), i.e. negative infinity.
## Chain 1:   Stan can't start sampling from this initial value.
## Chain 1: Rejecting initial value:
## Chain 1:   Log probability evaluates to log(0), i.e. negative infinity.
## Chain 1:   Stan can't start sampling from this initial value.
## Chain 1:
## Chain 1: Gradient evaluation took 0.001897 seconds
## Chain 1: 1000 transitions using 10 leapfrog steps per transition would take 18.97 seconds.
## Chain 1: Adjust your expectations accordingly!
## Chain 1:
## Chain 1:
## Chain 1: Iteration:    1 / 2000 [  0%]  (Warmup)
## Chain 1: Iteration:  200 / 2000 [ 10%]  (Warmup)
## Chain 1: Iteration:  400 / 2000 [ 20%]  (Warmup)
## Chain 1: Iteration:  600 / 2000 [ 30%]  (Warmup)
## Chain 1: Iteration:  800 / 2000 [ 40%]  (Warmup)
## Chain 1: Iteration: 1000 / 2000 [ 50%]  (Warmup)
## Chain 1: Iteration: 1001 / 2000 [ 50%]  (Sampling)
## Chain 1: Iteration: 1200 / 2000 [ 60%]  (Sampling)
## Chain 1: Iteration: 1400 / 2000 [ 70%]  (Sampling)
## Chain 1: Iteration: 1600 / 2000 [ 80%]  (Sampling)
## Chain 1: Iteration: 1800 / 2000 [ 90%]  (Sampling)
## Chain 1: Iteration: 2000 / 2000 [100%]  (Sampling)
## Chain 1:
## Chain 1:  Elapsed Time: 78.2325 seconds (Warm-up)
## Chain 1:                20.0374 seconds (Sampling)
## Chain 1:                98.2699 seconds (Total)
## Chain 1:
##
## SAMPLING FOR MODEL 'CSfit' NOW (CHAIN 2).
## Chain 2: Rejecting initial value:
## Chain 2:   Log probability evaluates to log(0), i.e. negative infinity.
## Chain 2:   Stan can't start sampling from this initial value.
## Chain 2: Rejecting initial value:
## Chain 2:   Log probability evaluates to log(0), i.e. negative infinity.
## Chain 2:   Stan can't start sampling from this initial value.
## Chain 2: Rejecting initial value:
## Chain 2:   Log probability evaluates to log(0), i.e. negative infinity.
## Chain 2:   Stan can't start sampling from this initial value.
## Chain 2: Rejecting initial value:
## Chain 2:   Log probability evaluates to log(0), i.e. negative infinity.
## Chain 2:   Stan can't start sampling from this initial value.
## Chain 2: Rejecting initial value:
## Chain 2:   Log probability evaluates to log(0), i.e. negative infinity.
## Chain 2:   Stan can't start sampling from this initial value.
## Chain 2: Rejecting initial value:
## Chain 2:   Log probability evaluates to log(0), i.e. negative infinity.
## Chain 2:   Stan can't start sampling from this initial value.
## Chain 2: Rejecting initial value:
```

```
## Chain 2:   Log probability evaluates to log(0), i.e. negative infinity.
## Chain 2:   Stan can't start sampling from this initial value.
## Chain 2: Rejecting initial value:
## Chain 2:   Log probability evaluates to log(0), i.e. negative infinity.
## Chain 2:   Stan can't start sampling from this initial value.
## Chain 2: Rejecting initial value:
## Chain 2:   Log probability evaluates to log(0), i.e. negative infinity.
## Chain 2:   Stan can't start sampling from this initial value.
## Chain 2: Rejecting initial value:
## Chain 2:   Log probability evaluates to log(0), i.e. negative infinity.
## Chain 2:   Stan can't start sampling from this initial value.
## Chain 2: Rejecting initial value:
## Chain 2:   Log probability evaluates to log(0), i.e. negative infinity.
## Chain 2:   Stan can't start sampling from this initial value.
## Chain 2: Rejecting initial value:
## Chain 2:   Log probability evaluates to log(0), i.e. negative infinity.
## Chain 2:   Stan can't start sampling from this initial value.
## Chain 2: Rejecting initial value:
## Chain 2:   Log probability evaluates to log(0), i.e. negative infinity.
## Chain 2:   Stan can't start sampling from this initial value.
## Chain 2: Rejecting initial value:
## Chain 2:   Log probability evaluates to log(0), i.e. negative infinity.
## Chain 2:   Stan can't start sampling from this initial value.
## Chain 2: Rejecting initial value:
## Chain 2:   Log probability evaluates to log(0), i.e. negative infinity.
## Chain 2:   Stan can't start sampling from this initial value.
## Chain 2: Rejecting initial value:
## Chain 2:   Log probability evaluates to log(0), i.e. negative infinity.
## Chain 2:   Stan can't start sampling from this initial value.
## Chain 2: Rejecting initial value:
## Chain 2:   Log probability evaluates to log(0), i.e. negative infinity.
## Chain 2:   Stan can't start sampling from this initial value.
## Chain 2: Rejecting initial value:
## Chain 2:   Log probability evaluates to log(0), i.e. negative infinity.
## Chain 2:   Stan can't start sampling from this initial value.
## Chain 2: Rejecting initial value:
## Chain 2:   Log probability evaluates to log(0), i.e. negative infinity.
## Chain 2:   Stan can't start sampling from this initial value.
## Chain 2: Rejecting initial value:
## Chain 2:   Log probability evaluates to log(0), i.e. negative infinity.
## Chain 2:   Stan can't start sampling from this initial value.
## Chain 2: Rejecting initial value:
## Chain 2:   Log probability evaluates to log(0), i.e. negative infinity.
## Chain 2:   Stan can't start sampling from this initial value.
## Chain 2: Rejecting initial value:
## Chain 2:   Log probability evaluates to log(0), i.e. negative infinity.
## Chain 2:   Stan can't start sampling from this initial value.
## Chain 2: Rejecting initial value:
## Chain 2:   Log probability evaluates to log(0), i.e. negative infinity.
## Chain 2:   Stan can't start sampling from this initial value.
## Chain 2: Rejecting initial value:
## Chain 2:   Log probability evaluates to log(0), i.e. negative infinity.
## Chain 2:   Stan can't start sampling from this initial value.
## Chain 2: Rejecting initial value:
```

```
## Chain 2:   Log probability evaluates to log(0), i.e. negative infinity.
## Chain 2:   Stan can't start sampling from this initial value.
## Chain 2: Rejecting initial value:
## Chain 2:   Log probability evaluates to log(0), i.e. negative infinity.
## Chain 2:   Stan can't start sampling from this initial value.
## Chain 2: Rejecting initial value:
## Chain 2:   Log probability evaluates to log(0), i.e. negative infinity.
## Chain 2:   Stan can't start sampling from this initial value.
## Chain 2: Rejecting initial value:
## Chain 2:   Log probability evaluates to log(0), i.e. negative infinity.
## Chain 2:   Stan can't start sampling from this initial value.
## Chain 2: Rejecting initial value:
## Chain 2:   Log probability evaluates to log(0), i.e. negative infinity.
## Chain 2:   Stan can't start sampling from this initial value.
## Chain 2: Rejecting initial value:
## Chain 2:   Log probability evaluates to log(0), i.e. negative infinity.
## Chain 2:   Stan can't start sampling from this initial value.
## Chain 2: Rejecting initial value:
## Chain 2:   Log probability evaluates to log(0), i.e. negative infinity.
## Chain 2:   Stan can't start sampling from this initial value.
## Chain 2: Rejecting initial value:
## Chain 2:   Log probability evaluates to log(0), i.e. negative infinity.
## Chain 2:   Stan can't start sampling from this initial value.
## Chain 2: Rejecting initial value:
## Chain 2:   Log probability evaluates to log(0), i.e. negative infinity.
## Chain 2:   Stan can't start sampling from this initial value.
## Chain 2: Rejecting initial value:
## Chain 2:   Log probability evaluates to log(0), i.e. negative infinity.
## Chain 2:   Stan can't start sampling from this initial value.
## Chain 2: Rejecting initial value:
## Chain 2:   Log probability evaluates to log(0), i.e. negative infinity.
## Chain 2:   Stan can't start sampling from this initial value.
## Chain 2: Rejecting initial value:
## Chain 2:   Log probability evaluates to log(0), i.e. negative infinity.
## Chain 2:   Stan can't start sampling from this initial value.
## Chain 2: Rejecting initial value:
## Chain 2:   Log probability evaluates to log(0), i.e. negative infinity.
## Chain 2:   Stan can't start sampling from this initial value.
## Chain 2: Rejecting initial value:
## Chain 2:   Log probability evaluates to log(0), i.e. negative infinity.
## Chain 2:   Stan can't start sampling from this initial value.
## Chain 2: Rejecting initial value:
## Chain 2:   Log probability evaluates to log(0), i.e. negative infinity.
## Chain 2:   Stan can't start sampling from this initial value.
## Chain 2: Rejecting initial value:
## Chain 2:   Log probability evaluates to log(0), i.e. negative infinity.
## Chain 2:   Stan can't start sampling from this initial value.
## Chain 2: Rejecting initial value:
## Chain 2:   Log probability evaluates to log(0), i.e. negative infinity.
## Chain 2:   Stan can't start sampling from this initial value.
## Chain 2: Rejecting initial value:
## Chain 2:   Log probability evaluates to log(0), i.e. negative infinity.
## Chain 2:   Stan can't start sampling from this initial value.
## Chain 2: Rejecting initial value:
```

```
## Chain 2:  Log probability evaluates to log(0), i.e. negative infinity.
## Chain 2:  Stan can't start sampling from this initial value.
## Chain 2: Rejecting initial value:
## Chain 2:  Log probability evaluates to log(0), i.e. negative infinity.
## Chain 2:  Stan can't start sampling from this initial value.
## Chain 2: Rejecting initial value:
## Chain 2:  Log probability evaluates to log(0), i.e. negative infinity.
## Chain 2:  Stan can't start sampling from this initial value.
## Chain 2: Rejecting initial value:
## Chain 2:  Log probability evaluates to log(0), i.e. negative infinity.
## Chain 2:  Stan can't start sampling from this initial value.
## Chain 2: Rejecting initial value:
## Chain 2:  Log probability evaluates to log(0), i.e. negative infinity.
## Chain 2:  Stan can't start sampling from this initial value.
## Chain 2: Rejecting initial value:
## Chain 2:  Log probability evaluates to log(0), i.e. negative infinity.
## Chain 2:  Stan can't start sampling from this initial value.
## Chain 2: Rejecting initial value:
## Chain 2:  Log probability evaluates to log(0), i.e. negative infinity.
## Chain 2:  Stan can't start sampling from this initial value.
## Chain 2: Rejecting initial value:
## Chain 2:  Log probability evaluates to log(0), i.e. negative infinity.
## Chain 2:  Stan can't start sampling from this initial value.
## Chain 2: Rejecting initial value:
## Chain 2:  Log probability evaluates to log(0), i.e. negative infinity.
## Chain 2:  Stan can't start sampling from this initial value.
## Chain 2: Rejecting initial value:
## Chain 2:  Log probability evaluates to log(0), i.e. negative infinity.
## Chain 2:  Stan can't start sampling from this initial value.
## Chain 2: Rejecting initial value:
## Chain 2:  Log probability evaluates to log(0), i.e. negative infinity.
## Chain 2:  Stan can't start sampling from this initial value.
## Chain 2: Rejecting initial value:
## Chain 2:  Log probability evaluates to log(0), i.e. negative infinity.
## Chain 2:  Stan can't start sampling from this initial value.
## Chain 2: Rejecting initial value:
## Chain 2:  Log probability evaluates to log(0), i.e. negative infinity.
## Chain 2:  Stan can't start sampling from this initial value.
## Chain 2: Rejecting initial value:
## Chain 2:  Log probability evaluates to log(0), i.e. negative infinity.
## Chain 2:  Stan can't start sampling from this initial value.
## Chain 2: Rejecting initial value:
## Chain 2:  Log probability evaluates to log(0), i.e. negative infinity.
## Chain 2:  Stan can't start sampling from this initial value.
## Chain 2: Rejecting initial value:
## Chain 2:  Log probability evaluates to log(0), i.e. negative infinity.
## Chain 2:  Stan can't start sampling from this initial value.
## Chain 2: Rejecting initial value:
## Chain 2:  Log probability evaluates to log(0), i.e. negative infinity.
## Chain 2:  Stan can't start sampling from this initial value.
## Chain 2: Rejecting initial value:
## Chain 2:  Log probability evaluates to log(0), i.e. negative infinity.
## Chain 2:  Stan can't start sampling from this initial value.
## Chain 2: Rejecting initial value:
```

```
## Chain 2:   Log probability evaluates to log(0), i.e. negative infinity.
## Chain 2:   Stan can't start sampling from this initial value.
## Chain 2: Rejecting initial value:
## Chain 2:   Log probability evaluates to log(0), i.e. negative infinity.
## Chain 2:   Stan can't start sampling from this initial value.
## Chain 2: Rejecting initial value:
## Chain 2:   Log probability evaluates to log(0), i.e. negative infinity.
## Chain 2:   Stan can't start sampling from this initial value.
## Chain 2: Rejecting initial value:
## Chain 2:   Log probability evaluates to log(0), i.e. negative infinity.
## Chain 2:   Stan can't start sampling from this initial value.
## Chain 2: Rejecting initial value:
## Chain 2:   Log probability evaluates to log(0), i.e. negative infinity.
## Chain 2:   Stan can't start sampling from this initial value.
## Chain 2: Rejecting initial value:
## Chain 2:   Log probability evaluates to log(0), i.e. negative infinity.
## Chain 2:   Stan can't start sampling from this initial value.
## Chain 2: Rejecting initial value:
## Chain 2:   Log probability evaluates to log(0), i.e. negative infinity.
## Chain 2:   Stan can't start sampling from this initial value.
## Chain 2: Rejecting initial value:
## Chain 2:   Log probability evaluates to log(0), i.e. negative infinity.
## Chain 2:   Stan can't start sampling from this initial value.
## Chain 2: Rejecting initial value:
## Chain 2:   Log probability evaluates to log(0), i.e. negative infinity.
## Chain 2:   Stan can't start sampling from this initial value.
## Chain 2: Rejecting initial value:
## Chain 2:   Log probability evaluates to log(0), i.e. negative infinity.
## Chain 2:   Stan can't start sampling from this initial value.
## Chain 2: Rejecting initial value:
## Chain 2:   Log probability evaluates to log(0), i.e. negative infinity.
## Chain 2:   Stan can't start sampling from this initial value.
## Chain 2: Rejecting initial value:
## Chain 2:   Log probability evaluates to log(0), i.e. negative infinity.
## Chain 2:   Stan can't start sampling from this initial value.
## Chain 2: Rejecting initial value:
## Chain 2:   Log probability evaluates to log(0), i.e. negative infinity.
## Chain 2:   Stan can't start sampling from this initial value.
## Chain 2: Rejecting initial value:
## Chain 2:   Log probability evaluates to log(0), i.e. negative infinity.
## Chain 2:   Stan can't start sampling from this initial value.
## Chain 2: Rejecting initial value:
## Chain 2:   Log probability evaluates to log(0), i.e. negative infinity.
## Chain 2:   Stan can't start sampling from this initial value.
## Chain 2: Rejecting initial value:
## Chain 2:   Log probability evaluates to log(0), i.e. negative infinity.
## Chain 2:   Stan can't start sampling from this initial value.
## Chain 2: Rejecting initial value:
## Chain 2:   Log probability evaluates to log(0), i.e. negative infinity.
## Chain 2:   Stan can't start sampling from this initial value.
## Chain 2: Rejecting initial value:
## Chain 2:   Log probability evaluates to log(0), i.e. negative infinity.
## Chain 2:   Stan can't start sampling from this initial value.
## Chain 2: Rejecting initial value:
```

```
## Chain 2:   Log probability evaluates to log(0), i.e. negative infinity.
## Chain 2:   Stan can't start sampling from this initial value.
## Chain 2: Rejecting initial value:
## Chain 2:   Log probability evaluates to log(0), i.e. negative infinity.
## Chain 2:   Stan can't start sampling from this initial value.
## Chain 2: Rejecting initial value:
## Chain 2:   Log probability evaluates to log(0), i.e. negative infinity.
## Chain 2:   Stan can't start sampling from this initial value.
## Chain 2: Rejecting initial value:
## Chain 2:   Log probability evaluates to log(0), i.e. negative infinity.
## Chain 2:   Stan can't start sampling from this initial value.
## Chain 2: Rejecting initial value:
## Chain 2:   Log probability evaluates to log(0), i.e. negative infinity.
## Chain 2:   Stan can't start sampling from this initial value.
## Chain 2: Rejecting initial value:
## Chain 2:   Log probability evaluates to log(0), i.e. negative infinity.
## Chain 2:   Stan can't start sampling from this initial value.
## Chain 2: Rejecting initial value:
## Chain 2:   Log probability evaluates to log(0), i.e. negative infinity.
## Chain 2:   Stan can't start sampling from this initial value.
## Chain 2: Rejecting initial value:
## Chain 2:   Log probability evaluates to log(0), i.e. negative infinity.
## Chain 2:   Stan can't start sampling from this initial value.
## Chain 2: Rejecting initial value:
## Chain 2:   Log probability evaluates to log(0), i.e. negative infinity.
## Chain 2:   Stan can't start sampling from this initial value.
## Chain 2: Rejecting initial value:
## Chain 2:   Log probability evaluates to log(0), i.e. negative infinity.
## Chain 2:   Stan can't start sampling from this initial value.
## Chain 2: Rejecting initial value:
## Chain 2:   Log probability evaluates to log(0), i.e. negative infinity.
## Chain 2:   Stan can't start sampling from this initial value.
## Chain 2: Rejecting initial value:
## Chain 2:   Log probability evaluates to log(0), i.e. negative infinity.
## Chain 2:   Stan can't start sampling from this initial value.
## Chain 2: Rejecting initial value:
## Chain 2:   Log probability evaluates to log(0), i.e. negative infinity.
## Chain 2:   Stan can't start sampling from this initial value.
## Chain 2:
## Chain 2: Gradient evaluation took 0.000896 seconds
## Chain 2: 1000 transitions using 10 leapfrog steps per transition would take 8.96 seconds.
## Chain 2: Adjust your expectations accordingly!
## Chain 2:
## Chain 2:
## Chain 2: Iteration:    1 / 2000 [  0%]  (Warmup)
## Chain 2: Iteration:  200 / 2000 [ 10%]  (Warmup)
## Chain 2: Iteration:  400 / 2000 [ 20%]  (Warmup)
## Chain 2: Iteration:  600 / 2000 [ 30%]  (Warmup)
## Chain 2: Iteration:  800 / 2000 [ 40%]  (Warmup)
## Chain 2: Iteration: 1000 / 2000 [ 50%]  (Warmup)
## Chain 2: Iteration: 1001 / 2000 [ 50%]  (Sampling)
## Chain 2: Iteration: 1200 / 2000 [ 60%]  (Sampling)
## Chain 2: Iteration: 1400 / 2000 [ 70%]  (Sampling)
## Chain 2: Iteration: 1600 / 2000 [ 80%]  (Sampling)
```

```
## Chain 2: Iteration: 1800 / 2000 [ 90%]  (Sampling)
## Chain 2: Iteration: 2000 / 2000 [100%]  (Sampling)
## Chain 2:
## Chain 2:  Elapsed Time: 90.6293 seconds (Warm-up)
## Chain 2:                50.9732 seconds (Sampling)
## Chain 2:                141.602 seconds (Total)
## Chain 2:
##
## SAMPLING FOR MODEL 'CSfit' NOW (CHAIN 3).
## Chain 3: Rejecting initial value:
## Chain 3:   Log probability evaluates to log(0), i.e. negative infinity.
## Chain 3:   Stan can't start sampling from this initial value.
## Chain 3: Rejecting initial value:
## Chain 3:   Log probability evaluates to log(0), i.e. negative infinity.
## Chain 3:   Stan can't start sampling from this initial value.
## Chain 3: Rejecting initial value:
## Chain 3:   Log probability evaluates to log(0), i.e. negative infinity.
## Chain 3:   Stan can't start sampling from this initial value.
## Chain 3: Rejecting initial value:
## Chain 3:   Log probability evaluates to log(0), i.e. negative infinity.
## Chain 3:   Stan can't start sampling from this initial value.
## Chain 3: Rejecting initial value:
## Chain 3:   Log probability evaluates to log(0), i.e. negative infinity.
## Chain 3:   Stan can't start sampling from this initial value.
## Chain 3: Rejecting initial value:
## Chain 3:   Log probability evaluates to log(0), i.e. negative infinity.
## Chain 3:   Stan can't start sampling from this initial value.
## Chain 3: Rejecting initial value:
## Chain 3:   Log probability evaluates to log(0), i.e. negative infinity.
## Chain 3:   Stan can't start sampling from this initial value.
## Chain 3: Rejecting initial value:
## Chain 3:   Log probability evaluates to log(0), i.e. negative infinity.
## Chain 3:   Stan can't start sampling from this initial value.
## Chain 3: Rejecting initial value:
## Chain 3:   Log probability evaluates to log(0), i.e. negative infinity.
## Chain 3:   Stan can't start sampling from this initial value.
## Chain 3: Rejecting initial value:
## Chain 3:   Log probability evaluates to log(0), i.e. negative infinity.
## Chain 3:   Stan can't start sampling from this initial value.
## Chain 3: Rejecting initial value:
## Chain 3:   Log probability evaluates to log(0), i.e. negative infinity.
## Chain 3:   Stan can't start sampling from this initial value.
## Chain 3:
## Chain 3: Gradient evaluation took 0.000991 seconds
## Chain 3: 1000 transitions using 10 leapfrog steps per transition would take 9.91 seconds.
## Chain 3: Adjust your expectations accordingly!
## Chain 3:
## Chain 3:
## Chain 3: Iteration:    1 / 2000 [  0%]  (Warmup)
## Chain 3: Iteration:  200 / 2000 [ 10%]  (Warmup)
## Chain 3: Iteration:  400 / 2000 [ 20%]  (Warmup)
## Chain 3: Iteration:  600 / 2000 [ 30%]  (Warmup)
## Chain 3: Iteration:  800 / 2000 [ 40%]  (Warmup)
## Chain 3: Iteration: 1000 / 2000 [ 50%]  (Warmup)
```

```
## Chain 3: Iteration: 1001 / 2000 [ 50%]  (Sampling)
## Chain 3: Iteration: 1200 / 2000 [ 60%]  (Sampling)
## Chain 3: Iteration: 1400 / 2000 [ 70%]  (Sampling)
## Chain 3: Iteration: 1600 / 2000 [ 80%]  (Sampling)
## Chain 3: Iteration: 1800 / 2000 [ 90%]  (Sampling)
## Chain 3: Iteration: 2000 / 2000 [100%]  (Sampling)
## Chain 3:
## Chain 3:  Elapsed Time: 90.676 seconds (Warm-up)
## Chain 3:                25.3047 seconds (Sampling)
## Chain 3:                115.981 seconds (Total)
## Chain 3:
```

```
## Warning: Bulk Effective Samples Size (ESS) is too low, indicating posterior means and medians may be
## Running the chains for more iterations may help. See
## http://mc-stan.org/misc/warnings.html#bulk-ess
```

```
## Warning: Tail Effective Samples Size (ESS) is too low, indicating posterior variances and tail quant
## Running the chains for more iterations may help. See
## http://mc-stan.org/misc/warnings.html#tail-ess
```

Chain convergence

```
model_params <- c("p", "q",
  "tau0",   "tauM",   "tauK",   "tauY",   "tauE",   "tauL",
  "alpha0", "alphaM", "alphaK", "alphaY", "alphaE", "alphaL",
  "phi", "sigmaTau", "sigmaAlpha", "nuTau", "nuAlpha")

# check for chain convergence (can also set chains = 3 above)
traceplot(fit, pars = model_params, inc_warmup = TRUE, ncol = 4)
```

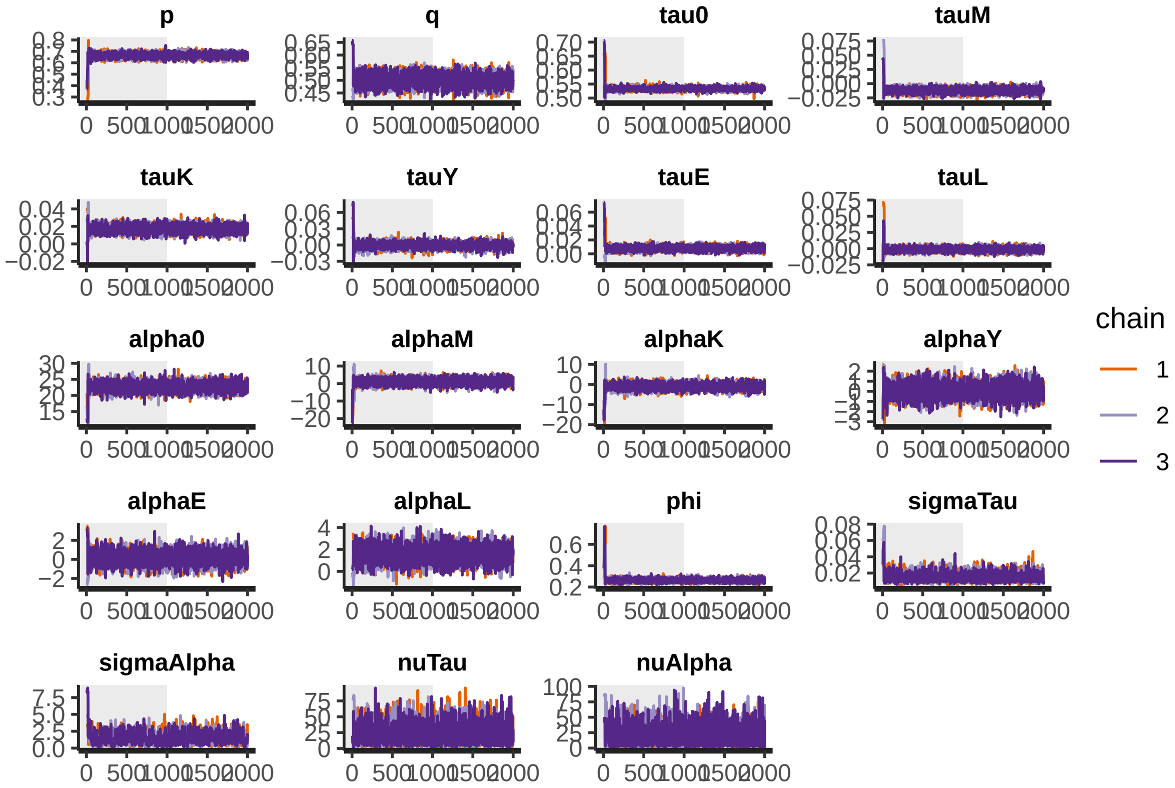

Figure 1: MCMC chain dynamics.

The chains have settled down after the 1000 burn-in.

Parameter correlations

```
mcmc_pairs(fit,
    pars = c("tau0", "tauM", "tauK", "tauY", "tauE", "tauL"),
  off_diag_fun = "hex"
)
```

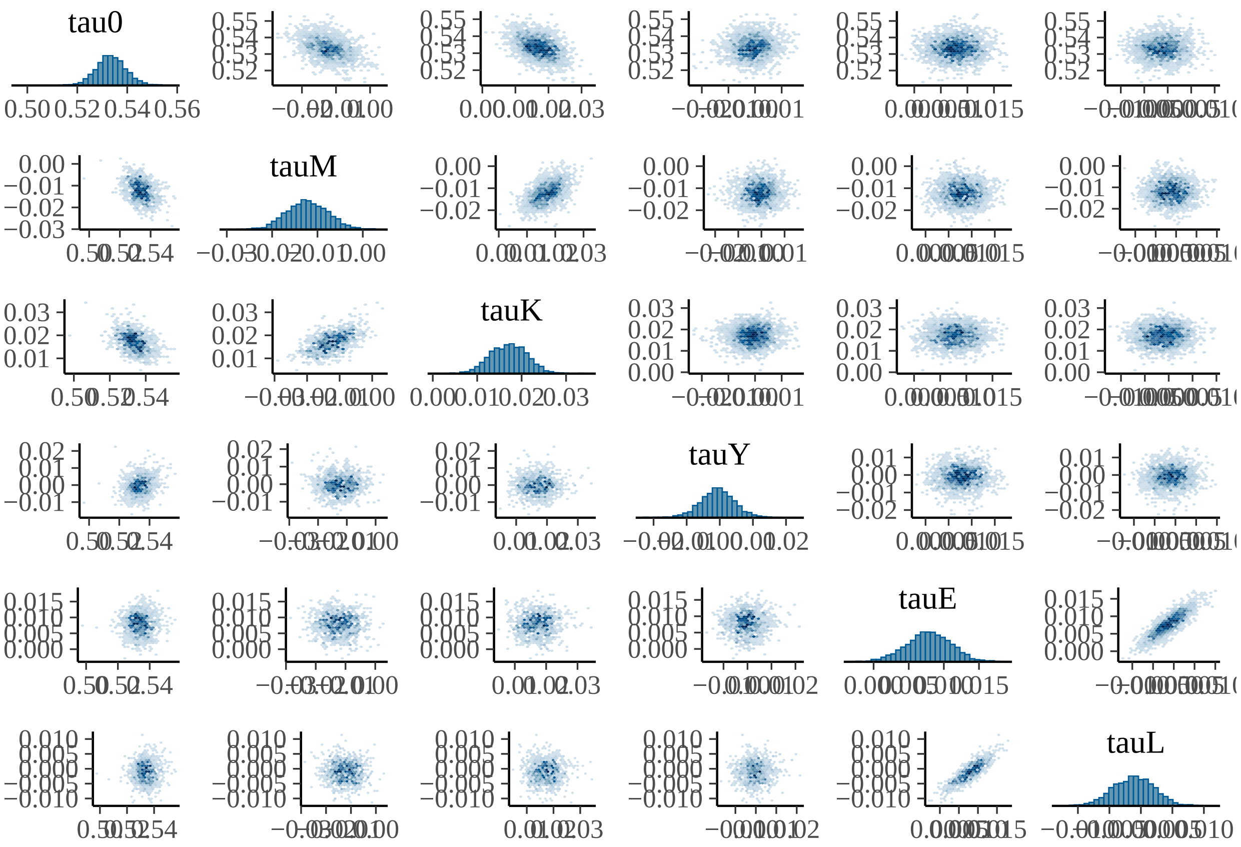


```
mcmc_pairs(fit,
    pars = c("alpha0", "alphaM", "alphaK", "alphaY", "alphaE", "alphaL"),
  off_diag_fun = "hex"
)
```

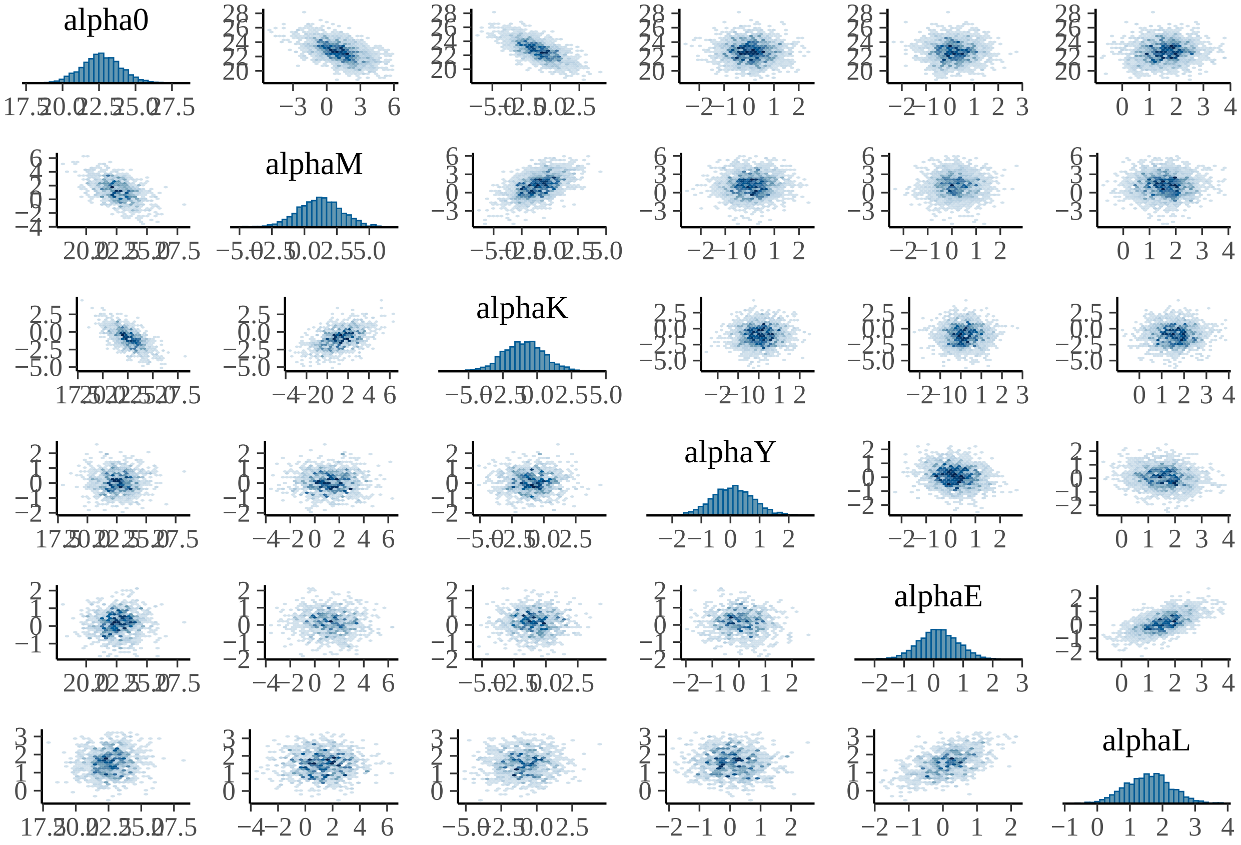

Figure 2: Correlations among parameters associated with change in the day of 50% shed (tau parameters) and the rate of shedding (alpha parameters).

For the most part the parameters are not highly correlated, except for those associated with elevation and latitude, more so for date of shedding. Deviations associated with males and presence of kid show slight correlation.

Parameter estimates

Table 1: Parameter estimates and 89% credible intervals. Note that the scale of many parameters are different to those reported in the manuscript (e.g. tau here is fraction of year but days in the manuscript).

```
model_params <- c("p", "q",
  "tau0",   "tauM",   "tauK",   "tauY",   "tauE",   "tauL",
  "alpha0", "alphaM", "alphaK", "alphaY", "alphaE", "alphaL",
  "phi", "sigmaTau", "sigmaAlpha", "nuTau", "nuAlpha")

# display the posterior distribution statistics
print(fit, pars = model_params, probs = c(0.055,0.5,0.945), digits=3)
```

```
## Inference for Stan model: CSfit.
## 3 chains, each with iter=2000; warmup=1000; thin=1;
## post-warmup draws per chain=1000, total post-warmup draws=3000.
##
##              mean se_mean    sd  5.5%   50% 94.5% n_eff  Rhat
## p           0.666   0.000 0.020 0.633 0.666 0.697  3494 0.999
```

```
## q           0.500   0.000  0.023  0.462  0.499  0.537  4678 0.999
## tau0        0.534   0.000  0.006  0.524  0.533  0.543  1393 1.002
## tauM       -0.012   0.000  0.005 -0.019 -0.012 -0.005  2874 1.000
## tauK        0.017   0.000  0.004  0.011  0.017  0.024  2607 0.999
## tauY       -0.001   0.000  0.005 -0.009 -0.001  0.008  1571 1.004
## tauE        0.008   0.000  0.003  0.003  0.008  0.013  2448 0.999
## tauL       -0.001   0.000  0.003 -0.006 -0.001  0.004  2356 0.999
## alpha0     22.658   0.028  1.334 20.486 22.652 24.805  2313 1.001
## alphaM      1.173   0.030  1.673 -1.522  1.197  3.841  3079 1.000
## alphaK     -0.981   0.028  1.471 -3.285 -0.956  1.356  2800 1.000
## alphaY      0.074   0.013  0.702 -1.025  0.074  1.196  2841 0.999
## alphaE      0.160   0.011  0.663 -0.875  0.152  1.221  3549 1.000
## alphaL      1.567   0.013  0.693  0.476  1.580  2.658  3063 1.000
## phi         0.263   0.000  0.016  0.238  0.263  0.289  3551 0.999
## sigmaTau    0.015   0.000  0.005  0.009  0.014  0.023  1701 1.000
## sigmaAlpha  1.494   0.044  0.786  0.345  1.423  2.894   316 1.003
## nuTau      21.996   0.245 14.155  5.354 18.975 48.808  3346 1.000
## nuAlpha    19.684   0.288 14.435  3.555 16.505 46.979  2519 1.000
##
## Samples were drawn using NUTS(diag_e) at Sun Sep 13 07:19:46 2020.
## For each parameter, n_eff is a crude measure of effective sample size,
## and Rhat is the potential scale reduction factor on split chains (at
## convergence, Rhat=1).
```

Males were estimated to be molting earlier than females and females with a kid were molting later ($\tau_M < 0$, $\tau_K > 0$). The model estimates molting to be occurring later at higher elevations ($\tau_E > 0$). There was also some evidence that molting occurred later at higher latitudes ($\tau_L > 0$). There was not strong evidence that the timing or rate of molting had changed consistently over the long-term ($\tau_Y, \alpha_Y \approx 0$). The rate of molting was not related to elevation ($\alpha_E \approx 0$), however it was positively related to latitude ($\alpha_L > 0$).

Parameter Credibility

```
mcmc_intervals(fit, pars = c("tauM", "tauK", "tauY", "tauE", "tauL"),
    prob_outer = 0.89)
```

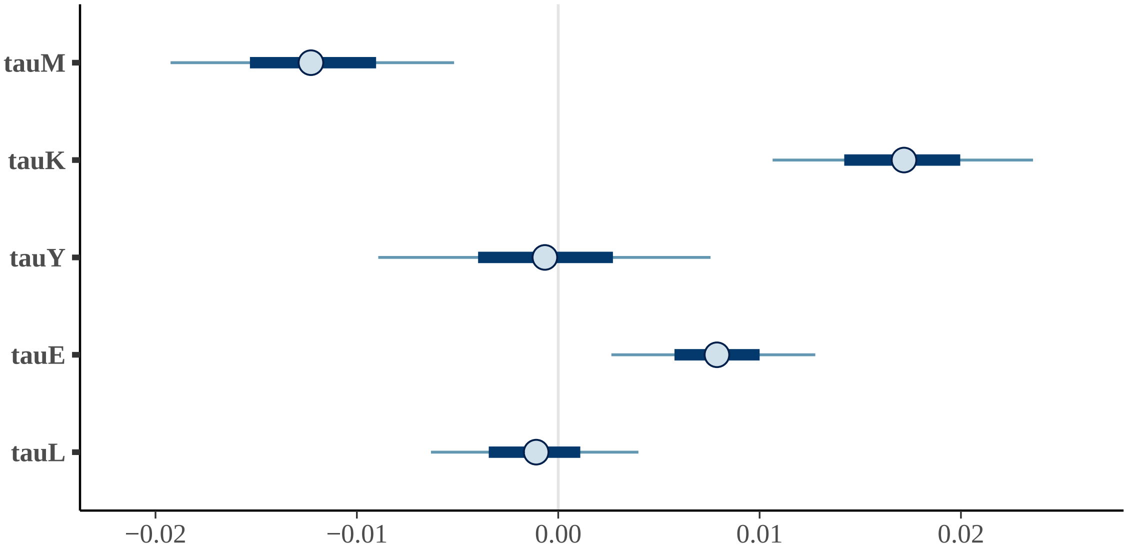

```r
mcmc_intervals(fit, pars = c("alphaM", "alphaK", "alphaY", "alphaE", "alphaL"),
    prob_outer = 0.89)
```

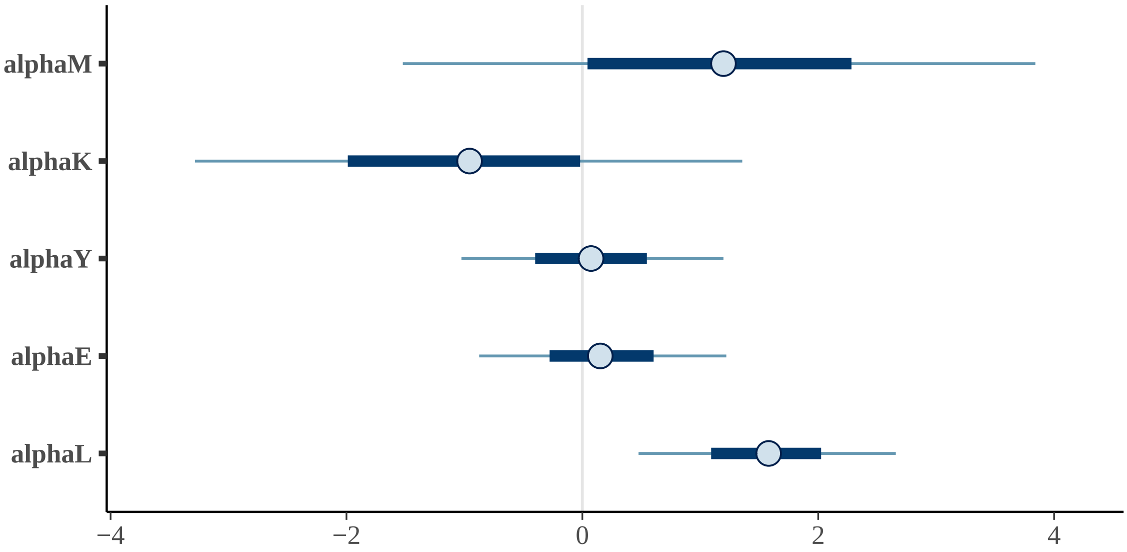

Figure 3: 89% posterior credible intervals for key model parameters describing effects due to animal state and environmental predictors.

Random effects

```r
model_params <- "year_tau_RE"
years <- levels(factor(df_fit$year))

l_params <- rstan::extract(fit, pars = model_params)
m_RE <- l_params$year_tau_RE
colnames(m_RE) <- as.character(years)
df_RE <- data.frame(m_RE)
names(df_RE) <- as.character(years)

df_RE$Rep <- 1:nrow(m_RE)
df_RE <- gather(df_RE, key = Year, value = RE, 1:length(years))
df_summary_A <- df_RE %>%
  group_by(Year) %>%
  summarise(
    Days = 365*mean(RE),
    low95 = 365*quantile(RE, probs = 0.055), # 89% credible bounds
    upp95 = 365*quantile(RE, probs = 0.945)
  )
```

```
## `summarise()` ungrouping output (override with `.groups` argument)
```

```r
p_3A <- ggplot(df_summary_A, aes(x = Year, y = Days))  +
  geom_hline(yintercept = 0, linetype = "dashed") +
  geom_point() +
  geom_errorbar(aes(ymin = low95, ymax = upp95), width = 0.2) +
```

```
  labs(y = "Change in\nmolting date (days)") +
  theme_bw() +
  theme(axis.text.x = element_text(angle = 30, hjust = 1, vjust=1))

model_params <- c("year_alpha_RE")
years <- levels(factor(df_fit$year))

l_params <- rstan::extract(fit, pars = model_params)
m_RE <- l_params$year_alpha_RE
colnames(m_RE) <- as.character(years)
df_RE <- data.frame(m_RE)
names(df_RE) <- as.character(years)
df_RE$Rep <- 1:nrow(m_RE)
df_RE <- gather(df_RE, key = Year, value = RE, 1:length(years))
df_summary_A <- df_RE %>%
  mutate(RE2 = 100*RE/(4*(365))) %>%
  group_by(Year) %>%
  summarise(
    Days = mean(RE2),
    low95 = quantile(RE2, probs = 0.055), # 89% credible bounds
    upp95 = quantile(RE2, probs = 0.945)
  )
```

```
## `summarise()` ungrouping output (override with `.groups` argument)
```

```
p_3B <- ggplot(df_summary_A, aes(x = Year, y = Days))  +
  geom_hline(yintercept = 0, linetype = "dashed") +
  geom_point() +
  geom_errorbar(aes(ymin = low95, ymax = upp95), width = 0.2) +
  labs(y = "Change in\nmolting rate (% per day)") +
  theme_bw() +
  theme(axis.text.x = element_text(angle = 30, hjust = 1, vjust=1))

plot_grid(p_3A, p_3B, ncol = 1, labels = c("A", "B"))
```

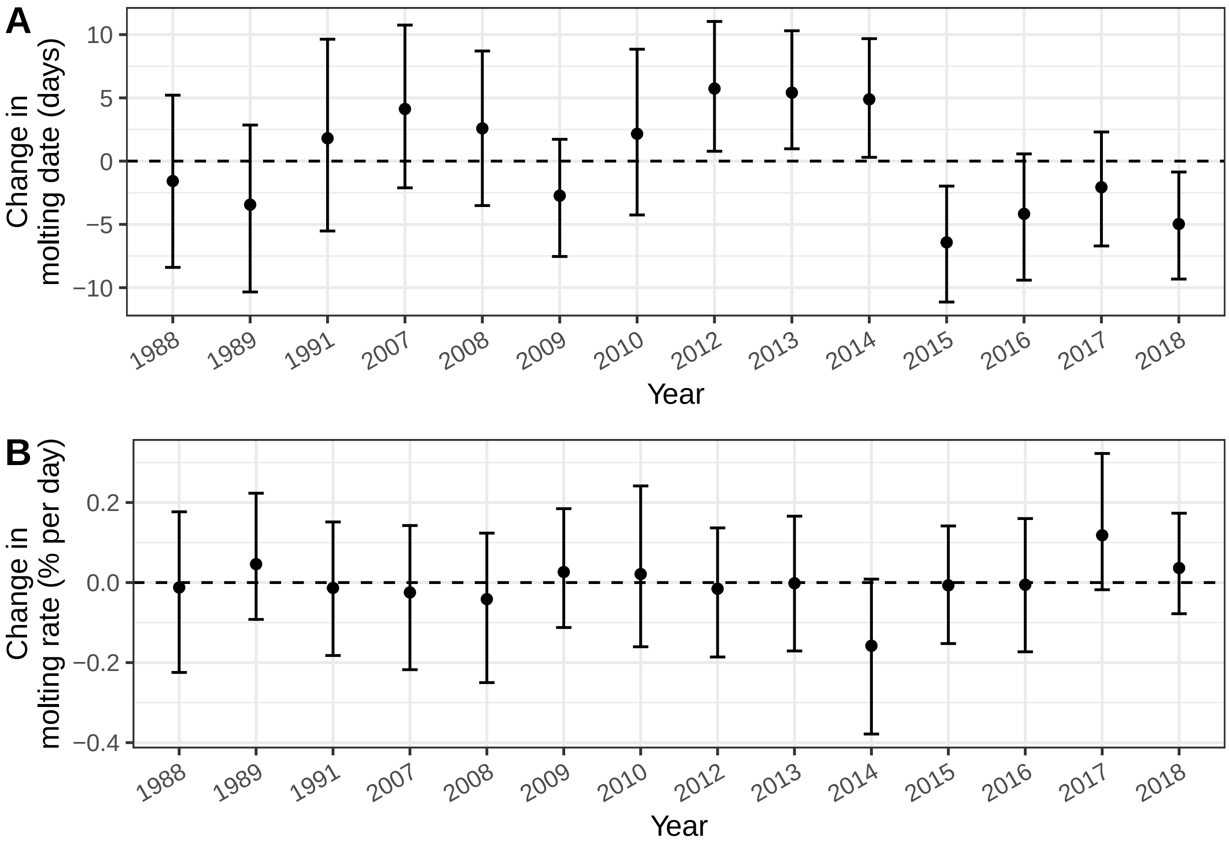

Figure 4: (A) Estimated annual deviates in the day of 50 molting relative to a female with no kid. Note that many of these estimates come from years with very few data, especially prior to 2008. (B) Estimated annual deviates in the rate of molting.

These deviates appear consistent with a t-distribution.

Predictions

Long-term trend

```
lat_predict = 0.0 # (60.871533 - Lat_mu) / Lat_sd # Yukon Wildlife Reserve
elv_predict = 0.0 # (753 - Elv_mu) / Elv_sd

model_params <- c("year_tau_RE", "tau0", "tauY", "tauL", "tauE")
years <- levels(factor(df_fit$year))

l_params <- rstan::extract(fit, pars = model_params)
m_RE <- l_params$year_tau_RE
colnames(m_RE) <- as.character(years)

df_tau_RE <- data.frame(m_RE)
names(df_tau_RE) <- as.character(years)

df_tau <- data.frame(
  Rep  = 1:nrow(m_RE),
```

```r
  tau0 = as.vector(l_params$tau0),
  tauY = as.vector(l_params$tauY),
  tauL = as.vector(l_params$tauL),
  tauE = as.vector(l_params$tauE)
)

df_tau <- cbind(df_tau, df_tau_RE)

df_yr <- data.frame(
    year = as.integer(1988:2018)
  ) %>%
  mutate(
    z_yr   = (year - yr_mu) / yr_sd,
    low89  =  0.0,
    median = 0.0,
    upp89  = 0.0
  )

for (i in 1:nrow(df_yr)) {
  tau <- df_tau$tau0 + df_tau$tauY*df_yr$z_yr[i] +
    df_tau$tauL*lat_predict + df_tau$tauE*elv_predict
  df_yr[i, 3:5] <- quantile(tau, probs = c(0.055, 0.5, 0.945))
}

df_yr$low89date  <- as.Date(365*df_yr$low89,  origin = "2018-01-01")
df_yr$mediandate <- as.Date(365*df_yr$median, origin = "2018-01-01")
df_yr$upp89date  <- as.Date(365*df_yr$upp89,  origin = "2018-01-01")

df_offset <- data.frame(
    year = as.integer(years)
  ) %>%
  mutate(
    z_yr   = (year - yr_mu) / yr_sd,
    low89  =  0.0,
    median = 0.0,
    upp89  = 0.0
  )

for (i in 1:nrow(df_offset)) {
  tau <- df_tau$tau0 + df_tau$tauY*df_offset$z_yr[i] +
    df_tau$tauL*lat_predict + df_tau$tauE*elv_predict + df_tau[,i+5]
  df_offset[i, 3:5] <- quantile(tau, probs = c(0.055, 0.5, 0.945))
}

df_offset$low89date  <- as.Date(365*df_offset$low89,  origin = "2018-01-01")
df_offset$mediandate <- as.Date(365*df_offset$median, origin = "2018-01-01")
df_offset$upp89date  <- as.Date(365*df_offset$upp89,  origin = "2018-01-01")
```

```r
df_offset <- filter(df_offset, year >= 1980)

ggplot() +
  geom_ribbon(data = df_yr,
    aes(x = year, ymin = low89date, ymax = upp89date), fill = "grey75") +
```

```r
  geom_errorbar(data = df_offset,
    aes(x = year, ymin = low89date, ymax = upp89date),
    color = "black", width = 0) +
  geom_line(data = df_yr,
    aes(x = year, y = mediandate), color = "grey50", linetype = "dashed") +
  geom_point(data = df_offset,
    aes(x = year, y = mediandate), color = "black") +
  labs(x = "Year", y = "Date when 50% shed") +
  theme_bw()
```

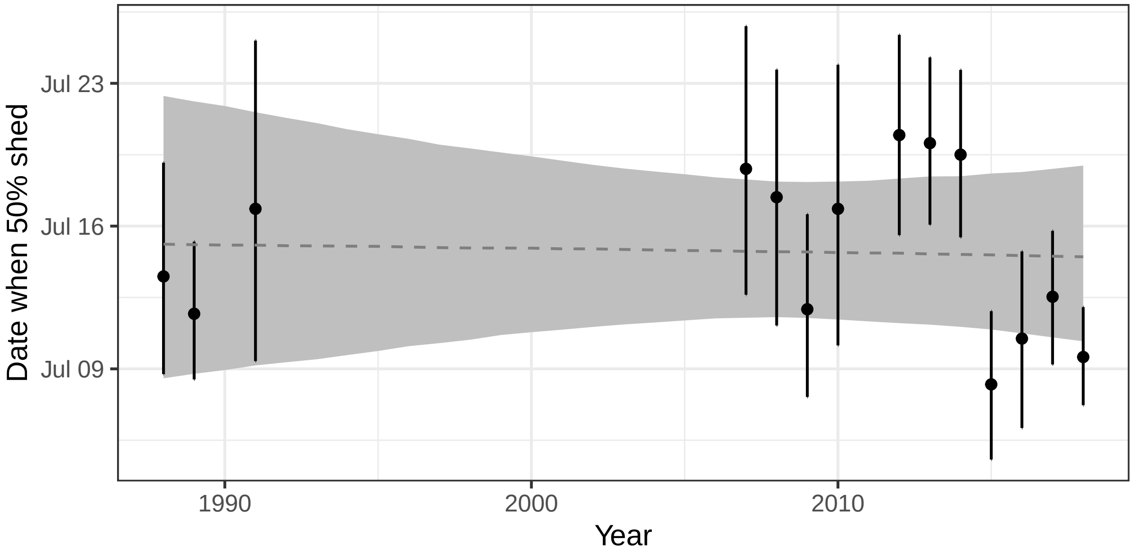

Figure 5: Predicted dates for females without kid (state FN) having shed 50% of their coat when at a site defined by z-transformed predictors being zero (i.e. latitude 49.14 and elevation 2025 m). Dashed line is the long-term trend and shaded region is the 89% CI. Estimated yearly fluctuations about the trend are also presented along with their 89% CI.

Within-season

```r
# specify environmental conditions for the predictions
z_lat   <- 0.0 # (60.87 - Lat_mu) /Lat_sd
z_year  <- (2018 - yr_mu) / yr_sd
z_ele  <- 0.0 # (753 - Elv_mu) / Elv_sd

model_params <- c("p", "q",
  "tau0",   "tauM",   "tauK",   "tauY",   "tauE",   "tauL",
  "alpha0", "alphaM", "alphaK", "alphaY", "alphaE", "alphaL")
l_params <- rstan::extract(fit, pars = model_params)

REPS    <- length(l_params$tau0) # samples to choose from
SAMPLES <- 100                   # random posterior samples
indxs   <- sample(1:REPS, SAMPLES, replace = FALSE)

# define temporal range of predictions
t_min   <- 100
t_max   <- 300
```

```r
v_t     <- seq(from = t_min, to = t_max, by = 1)/365
v_date  <- 365*v_t # as.Date(365*v_t, origin = "2018-01-01")
n_t     <- length(v_t)

df_low <- tibble(doy = v_date, FN = 0.0, FY = 0.0, MN = 0.0)
df_med <- tibble(doy = v_date, FN = 0.0, FY = 0.0, MN = 0.0)
df_upp <- tibble(doy = v_date, FN = 0.0, FY = 0.0, MN = 0.0)

for (i in 1:n_t) {
    t <- v_t[i]
  # extract posterior model parameters
  tau0   <- l_params$tau0[indxs]
  tauY   <- l_params$tauY[indxs]
  tauE   <- l_params$tauE[indxs]
  tauL   <- l_params$tauL[indxs]
  tauK   <- l_params$tauK[indxs]
  tauM   <- l_params$tauM[indxs]
  alpha0 <- l_params$alpha0[indxs]
  alphaY <- l_params$alphaY[indxs]
  alphaE <- l_params$alphaE[indxs]
  alphaL <- l_params$alphaL[indxs]
  alphaK <- l_params$alphaK[indxs]
  alphaM <- l_params$alphaM[indxs]

  # baseline date of shedding
  tau_i <- tau0 + tauE*z_ele + tauL*z_lat + tauY*z_year
  # baseline rate of shedding
  alpha_i <- alpha0 + alphaE*z_ele + alphaL*z_lat + alphaY*z_year

  logit_FN <- alpha_i*(t - tau_i)
  logit_FY <- (alpha_i + alphaK)*(t - tau_i - tauK)
  logit_MN <- (alpha_i + alphaM)*(t - tau_i - tauM)

  logit_FN <- exp(logit_FN) / (1.0 + exp(logit_FN))
  logit_FY <- exp(logit_FY) / (1.0 + exp(logit_FY))
  logit_MN <- exp(logit_MN) / (1.0 + exp(logit_MN))

  v_CI <- quantile(logit_FN, probs = c(0.055, 0.5, 0.945))
  df_low$FN[i] <- v_CI[1]
  df_med$FN[i] <- v_CI[2]
  df_upp$FN[i] <- v_CI[3]

  v_CI <- quantile(logit_FY, probs = c(0.055, 0.5, 0.945))
  df_low$FY[i] <- v_CI[1]
  df_med$FY[i] <- v_CI[2]
  df_upp$FY[i] <- v_CI[3]

  v_CI <- quantile(logit_MN, probs = c(0.055, 0.5, 0.945))
  df_low$MN[i] <- v_CI[1]
  df_med$MN[i] <- v_CI[2]
  df_upp$MN[i] <- v_CI[3]
}
```

```r
df_low <- gather(df_low, key = SK, value = low89, 2:4)
df_med <- gather(df_med, key = SK, value = med89, 2:4)
df_upp <- gather(df_upp, key = SK, value = upp89, 2:4)

df_CI <- df_low
df_CI$upp89 <- df_upp$upp89
```

```r
df_CI$SK <- factor(df_CI$SK)
levels(df_CI$SK)[levels(df_CI$SK)=="FN"] <- "FN (female, no kid)"
df_fit$SK <- factor(df_fit$SK)
levels(df_fit$SK)[levels(df_fit$SK)=="FN"] <- "FN (female, no kid)"
df_med$SK <- factor(df_med$SK)
levels(df_med$SK)[levels(df_med$SK)=="FN"] <- "FN (female, no kid)"

df_CI$SK <- factor(df_CI$SK)
levels(df_CI$SK)[levels(df_CI$SK)=="FY"] <- "FY (female with kid)"
df_fit$SK <- factor(df_fit$SK)
levels(df_fit$SK)[levels(df_fit$SK)=="FY"] <- "FY (female with kid)"
df_med$SK <- factor(df_med$SK)
levels(df_med$SK)[levels(df_med$SK)=="FY"] <- "FY (female with kid)"

df_CI$SK <- factor(df_CI$SK)
levels(df_CI$SK)[levels(df_CI$SK)=="MN"] <- "MN (male)"
df_fit$SK <- factor(df_fit$SK)
levels(df_fit$SK)[levels(df_fit$SK)=="MN"] <- "MN (male)"
df_med$SK <- factor(df_med$SK)
levels(df_med$SK)[levels(df_med$SK)=="MN"] <- "MN (male)"

ggplot() +
    geom_ribbon(data = df_CI,
        aes(x = doy, ymin = low89, ymax = upp89, fill = SK), alpha = 0.5) +
    geom_line(data = df_med, aes(x = doy, y = med89, color = SK)) +
    geom_point(data = filter(df_fit, SK %in% c("FN (female, no kid)",
        "FY (female with kid)", "MN (male)")),
        aes(x = doy, y = frac_shed, color = SK)) +
    scale_colour_manual(values=c("#980043", "#e7298a", "blue")) +
    scale_fill_manual(values=c("#980043", "#e7298a", "blue")) +
  labs(
    x = "Day of year",
    y = "Fraction shed",
    color = "Animal\nstate") +
    guides(fill = FALSE, color = FALSE) +
    facet_wrap( ~ SK) +
  theme_bw()
```

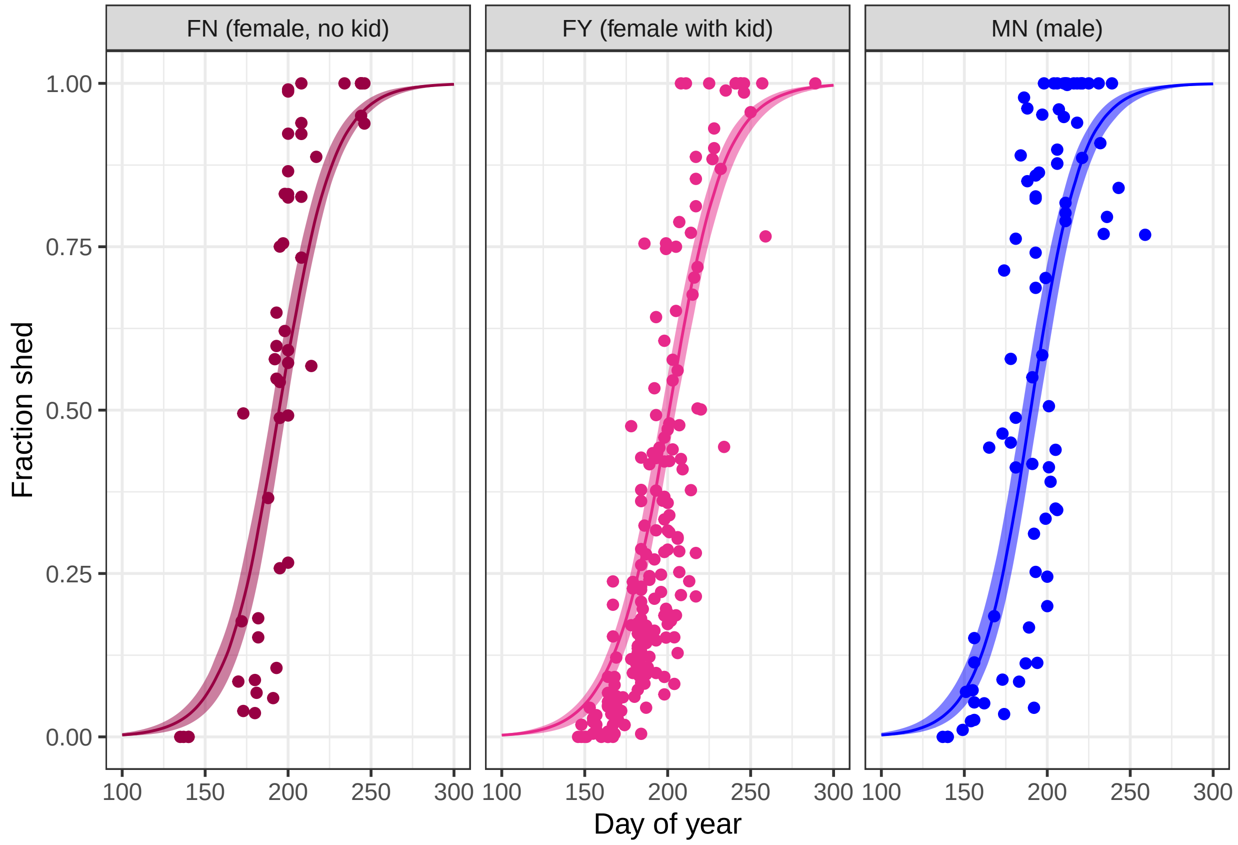

Figure 6: Observed and predicted shedding patterns. Panels correspond to the three animal states where sex and kid status are known. Shedding fractions for all photos where animal state was known are presented (points). The predictions are for 2018 at location defined by the z-transformed predictors being zero, which corresponds to latitude 49.14 and elevation 2025 m. The predictions also correspond to all random effect terms being set to zero. Solid lines depict the median shedding fraction and shaded regions are the associated 89% credible intervals.

Stan code

Stan code used to describe the shedding model.

```
writeLines(readLines("CSfit.stan"))
```

```
## Warning in readLines("CSfit.stan"): incomplete final line found on 'CSfit.stan'
```

```
## // Citisen science mountain goat shedding model
## // Shane.Richards@utas.edu.au
## // CSfit.stan
## // 06/08/2020
##
## data {
##   int<lower=1>                I;     // Number of animals (photos)
##   int<lower=1>                N;     // Number of shedding blocks
##   int<lower=1>                Y;     // Number of distinct years of data
##   int<lower=1,upper=6>    State[I];  // Animal state: FN, FY, FX, MN, XN, XX
```

```
##   real<lower=-5.0,upper=5.0> zt[I];  // z-day of year [0,1]
##   real<lower=-5.0,upper=5.0> ze[I];  // z-elevation
##   real<lower=-5.0,upper=5.0> zl[I];  // z-latitude
##   real<lower=-6.0,upper=5.0> zy[I];  // z-year [0.1,1.8]
##   int<lower=0,upper=Y>       iy[I];  // year index [1,Y]
##   int<lower=0,upper=N>        n[I];  // number of shed blocks [0,N]
## }
##
## parameters {
##   real <lower=0.2,upper=0.8>    tau0;       // base-line shed date
##   real <lower=10,upper=50>      alpha0;     // base-line shed rate
##   real <lower=-0.1,upper=0.1>   tauE;       // elevation effect on shed date
##   real <lower=-0.1,upper=0.1>   tauL;       // latitude effect on shed date
##   real <lower=-0.1,upper=0.1>   tauY;       // year effect on shed date
##   real <lower=-0.1,upper=0.1>   tauM;       // male effect on shed date
##   real <lower=-0.1,upper=0.1>   tauK;       // kid effect on shed date
##   real <lower=-5.0,upper=5.0>   alphaE;     // elevation effect on shed rate
##   real <lower=-5.0,upper=5.0>   alphaL;     // latitude effect on shed rate
##   real <lower=-5.0,upper=5.0>   alphaY;     // year effect on shed rate
##   real <lower=-50.0,upper=50.0> alphaM;     // male effect on shed rate
##   real <lower=-50.0,upper=50.0> alphaK;     // kid effect on shed rate
##   real <lower=0.001,upper=0.1>  sigmaTau;   // year-year random (shed date)
##   real <lower=0.001,upper=10.0> sigmaAlpha; // year-year random (shed rate)
##   real <lower=0.1,upper=100.0>  nuTau;      // df for t-dist (shed date)
##   real <lower=0.1,upper=100.0>  nuAlpha;    // df for t-dist (shed rate)
##   real <lower=0.2,upper=0.8>    p;          // fraction female
##   real <lower=0.2,upper=0.8>    q;          // fraction females with kid
##   real <lower=0.001,upper=1.0>  phi;        // beta-binomial variation term
##
##   vector[Y] year_tau_RE;   // inter-annual random effect for shed date
##   vector[Y] year_alpha_RE; // inter-annual random effect for shed rate
## }
##
## model {
##   real logit_FN; // logit: females without kid
##   real logit_FY; // logit: females with kid
##   real logit_MN; // logit: males
##   real mu;       // mean shed fraction for observation
##   real a;        // beta-binomial parameter
##   real b;        // beta-binomial parameter
##   int  y;        // observed shed number
##   real tau_i;    // inter-annual random effect term: shed date
##   real alpha_i;  // inter-annual random effect term: shed rate
##
##   // priors (in additon to uniform restrictions set above)
##   p       ~ beta(2,2); // proportion of animals that are female
##   q       ~ beta(2,2); // proportion of females with kid
##   //   shedding date parameters
##   tau0    ~ beta(2,2);
##   tauE    ~ normal(0,0.1);
##   tauL    ~ normal(0,0.1);
##   tauY    ~ normal(0,0.1);
##   tauM    ~ normal(0,0.1);
##   tauK    ~ normal(0,0.1);
```

```
##   //   shedding rate parameters
##   alpha0  ~ normal(25,5);
##   alphaE  ~ normal(0,1.0);
##   alphaL  ~ normal(0,1.0);
##   alphaY  ~ normal(0,1.0);
##   alphaM  ~ normal(0,20.0);
##   alphaK  ~ normal(0,20.0);
##   //   uncertainty parameters
##   sigmaTau   ~ exponential(10.0);
##   sigmaAlpha ~ exponential(1);
##   nuTau      ~ gamma(2, 0.1);
##   nuAlpha    ~ gamma(2, 0.1);
##   phi        ~ exponential(1.0);
##   //   inter-annual random effects
##   year_tau_RE   ~ student_t(nuTau, 0.0, sigmaTau);      // year-year random effect date
##   year_alpha_RE ~ student_t(nuAlpha, 0.0, sigmaAlpha);  // year-year random effect rate
##
##   for (i in 1:I) { // for each photo
##      // set base-line shedding date (tau) and shedding rate (alpha)
##     tau_i = tau0 + tauE*ze[i] + tauL*zl[i] + tauY*zy[i] +
##       year_tau_RE[iy[i]]; // day of year when 50% shed (female, no kid)
##     alpha_i = alpha0 + alphaE*ze[i] + alphaL*zl[i] + alphaY*zy[i] +
##       year_alpha_RE[iy[i]];  // max rate of shedding (female, no kid)
##     // modify tau and alpha based on animal state
##     if (State[i] == 1) { // FN
##       logit_FN = alpha_i*(zt[i] - tau_i);
##       mu = exp(logit_FN) / (1.0 + exp(logit_FN));
##       a = mu / phi;
##       b = (1.0 - mu) / phi;
##       y = n[i];
##       target += lgamma(N+1) + lgamma(a+b) + lgamma(y+a) +
##         lgamma(N-y+b) - lgamma(y+1) - lgamma(N-y+1) -
##         lgamma(a) - lgamma(b) - lgamma(N+a+b);
##     } else if (State[i] == 2) { // FY
##       logit_FY = (alpha_i + alphaK)*(zt[i] - tau_i - tauK);
##       mu = exp(logit_FY) / (1.0 + exp(logit_FY));
##       a = mu / phi;
##       b = (1.0 - mu) / phi;
##       y = n[i];
##       target += lgamma(N+1) + lgamma(a+b) + lgamma(y+a) +
##         lgamma(N-y+b) - lgamma(y+1) - lgamma(N-y+1) -
##         lgamma(a) - lgamma(b) - lgamma(N+a+b);
##     } else if (State[i] == 3) { // FX
##       logit_FN = alpha_i*(zt[i] - tau_i); // FN
##       mu = exp(logit_FN) / (1.0 + exp(logit_FN));
##       a = mu / phi;
##       b = (1.0 - mu) / phi;
##       y = n[i];
##       target += log(1.0 - q) + lgamma(N+1) + lgamma(a+b) + lgamma(y+a) +
##         lgamma(N-y+b) - lgamma(y+1) - lgamma(N-y+1) -
##         lgamma(a) - lgamma(b) - lgamma(N+a+b);
##
##       logit_FY = (alpha_i + alphaK)*(zt[i] - tau_i - tauK); // FY
##       mu = exp(logit_FY) / (1.0 + exp(logit_FY));
```

```
##       a = mu / phi;
##       b = (1.0 - mu) / phi;
##       y = n[i];
##       target += log(q) + lgamma(N+1) + lgamma(a+b) + lgamma(y+a) +
##         lgamma(N-y+b) - lgamma(y+1) - lgamma(N-y+1) -
##         lgamma(a) - lgamma(b) - lgamma(N+a+b);
##     } else if (State[i] == 4) { // MN
##       logit_MN = (alpha_i + alphaM)*(zt[i] - tau_i - tauM);
##       mu = exp(logit_MN) / (1.0 + exp(logit_MN));
##       a = mu / phi;
##       b = (1.0 - mu) / phi;
##       y = n[i];
##       target += lgamma(N+1) + lgamma(a+b) + lgamma(y+a) +
##         lgamma(N-y+b) - lgamma(y+1) - lgamma(N-y+1) -
##         lgamma(a) - lgamma(b) - lgamma(N+a+b);
##     } else if (State[i] == 5) { // XN
##       logit_FN = alpha_i*(zt[i] - tau_i); // FN
##       mu = exp(logit_FN) / (1.0 + exp(logit_FN));
##       a = mu / phi;
##       b = (1.0 - mu) / phi;
##       y = n[i];
##       target += log(p*(1-q)/(p*(1-q)+(1-p))) + lgamma(N+1) + lgamma(a+b) + lgamma(y+a) +
##         lgamma(N-y+b) - lgamma(y+1) - lgamma(N-y+1) -
##         lgamma(a) - lgamma(b) - lgamma(N+a+b);
##
##       logit_MN = (alpha_i + alphaM)*(zt[i] - tau_i - tauM); // MN
##       mu = exp(logit_MN) / (1.0 + exp(logit_MN));
##       a = mu / phi;
##       b = (1.0 - mu) / phi;
##       y = n[i];
##       target += log((1-p)/(p*(1-q)+(1-p))) + lgamma(N+1) + lgamma(a+b) + lgamma(y+a) +
##         lgamma(N-y+b) - lgamma(y+1) - lgamma(N-y+1) -
##         lgamma(a) - lgamma(b) - lgamma(N+a+b);
##     } else { // XX
##       logit_FN = alpha_i*(zt[i] - tau_i); // FN
##       mu = exp(logit_FN) / (1.0 + exp(logit_FN));
##       a = mu / phi;
##       b = (1.0 - mu) / phi;
##       y = n[i];
##       target += log(p*(1.0 - q)) + lgamma(N+1) + lgamma(a+b) + lgamma(y+a) +
##         lgamma(N-y+b) - lgamma(y+1) - lgamma(N-y+1) -
##         lgamma(a) - lgamma(b) - lgamma(N+a+b);
##
##       logit_FY = (alpha_i + alphaK)*(zt[i] - tau_i - tauK); // FY
##       mu = exp(logit_FY) / (1.0 + exp(logit_FY));
##       a = mu / phi;
##       b = (1.0 - mu) / phi;
##       y = n[i];
##       target += log(p*q) + lgamma(N+1) + lgamma(a+b) + lgamma(y+a) +
##         lgamma(N-y+b) - lgamma(y+1) - lgamma(N-y+1) -
##         lgamma(a) - lgamma(b) - lgamma(N+a+b);
##
##       logit_MN = (alpha_i + alphaM)*(zt[i] - tau_i - tauM); // MN
##       mu = exp(logit_MN) / (1.0 + exp(logit_MN));
```

```
##       a = mu / phi;
##       b = (1.0 - mu) / phi;
##       y = n[i];
##       target += log(1.0-p) + lgamma(N+1) + lgamma(a+b) + lgamma(y+a) +
##         lgamma(N-y+b) - lgamma(y+1) - lgamma(N-y+1) -
##         lgamma(a) - lgamma(b) - lgamma(N+a+b);
##     }
##   }
## }
```
