# Supplementary material for: Using community photography to investigate phenology: A case study of coat molt in the mountain goat (Oreamnos americanus) with missing data
Source: Ecol Evol. 2020 Nov 9;10(23):13488–99. doi: 10.1002/ece3.6954 (PMC7713987; doi:10.1002/ece3.6954)
Supplement: Supplementary file 3 — Supinfo3 [file ECE3-10-13488-s003.pdf]

Supplementary Materials 3: Yukon Wildlife Preserve Analysis

Shane A. Richards

2020-09-13

Contents

Summary **1**

Data **1**

Bayesian analysis **3**

Fit the model . . . 3

Chain convergence . . . 7

Parameter correlations . . . 7

Parameter estimates . . . 9

Individual variation . . . 10

Model Predictions **11**

Stan code **13**

Summary

This document supports the manuscript:

> Nowak et al. "Using community photography to investigate phenology: a case study of coat molt in the mountain goat (Oreamnos americanus) with missing data" submitted to Ecology and Evolution.

Here we present the statistical analysis of the Yukon Wildlife Preserve data set.

Data

```
SHED <- 25 # bins that define molting state

# create appropriate factors
df_CS$Sex  <- factor(df_CS$Sex)
df_CS$Sex  <- fct_relevel(df_CS$Sex,  "F", "M", "X")
df_CS$Kids <- factor(df_CS$Kids)
df_CS$Kids <- fct_relevel(df_CS$Kids, "N", "Y", "X")
# two letter code for animal state
df_CS$SK   <- factor(df_CS$SK)
df_CS$SK   <- fct_relevel(df_CS$SK, "FN", "FY", "FX", "MN", "XN", "XX")

df_CS <- arrange(df_CS, DateObs, SK, Lat)

df_YWP$SK <- fct_relevel(df_YWP$SK, "FN", "FY", "MN")
df_YWP$ID <- as.integer(factor(df_YWP$Individual_ID))
```

```
ggplot() +
    geom_line(data = df_YWP,
        aes(x = doy, y = percent_shed, color = SK, group = Individual_ID)) +
  geom_point(data = df_YWP,
    aes(x = doy, y = percent_shed, color = SK)) +
    geom_point(data = filter(df_CS, Lat > 60.87, Lat < 60.89, Long > -135.35, Long < -135.33),
        aes(x = doy, y = frac_shed, fill = SK), shape = 21) +
  labs(x = "Date" , y = "Fraction shed", color = "Animal\nstate") +
    scale_colour_manual(values=c("#980043", "#e7298a", "blue")) +
  theme_bw() +
  theme(
    panel.grid.minor = element_blank()
  )
```

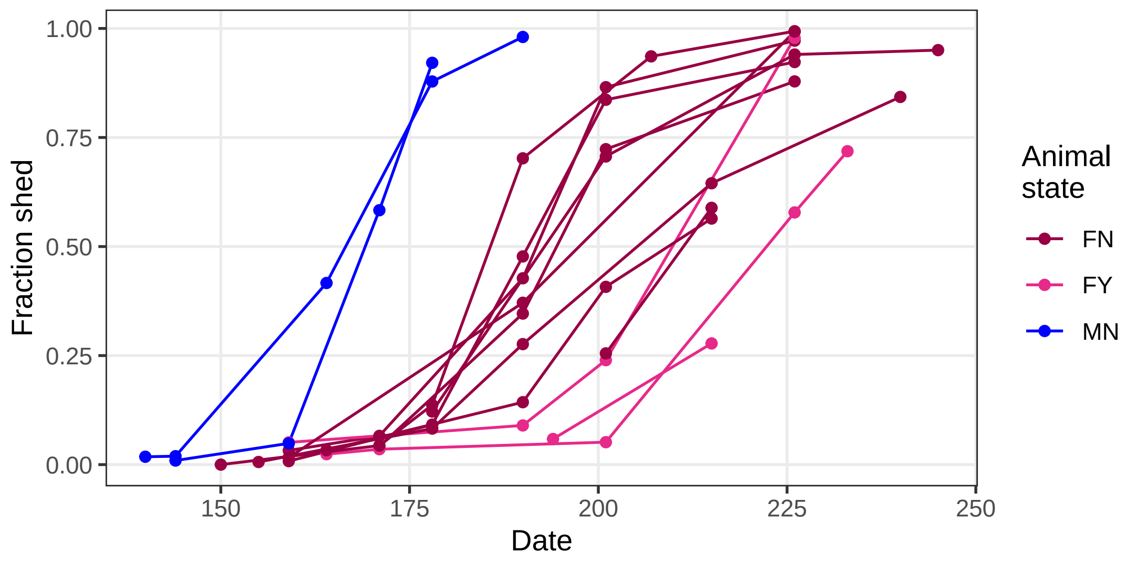

Figure 1: Shedding fractions observed for 14 animals during 2018.

Bayesian analysis

Fit the model

```
##
## SAMPLING FOR MODEL 'YWP' NOW (CHAIN 1).
## Chain 1: Rejecting initial value:
## Chain 1:   Log probability evaluates to log(0), i.e. negative infinity.
## Chain 1:   Stan can't start sampling from this initial value.
## Chain 1: Rejecting initial value:
## Chain 1:   Log probability evaluates to log(0), i.e. negative infinity.
## Chain 1:   Stan can't start sampling from this initial value.
## Chain 1: Rejecting initial value:
## Chain 1:   Log probability evaluates to log(0), i.e. negative infinity.
## Chain 1:   Stan can't start sampling from this initial value.
## Chain 1: Rejecting initial value:
## Chain 1:   Log probability evaluates to log(0), i.e. negative infinity.
## Chain 1:   Stan can't start sampling from this initial value.
## Chain 1: Rejecting initial value:
## Chain 1:   Log probability evaluates to log(0), i.e. negative infinity.
## Chain 1:   Stan can't start sampling from this initial value.
## Chain 1: Rejecting initial value:
## Chain 1:   Log probability evaluates to log(0), i.e. negative infinity.
## Chain 1:   Stan can't start sampling from this initial value.
## Chain 1: Rejecting initial value:
## Chain 1:   Log probability evaluates to log(0), i.e. negative infinity.
## Chain 1:   Stan can't start sampling from this initial value.
## Chain 1: Rejecting initial value:
## Chain 1:   Log probability evaluates to log(0), i.e. negative infinity.
## Chain 1:   Stan can't start sampling from this initial value.
## Chain 1: Rejecting initial value:
## Chain 1:   Log probability evaluates to log(0), i.e. negative infinity.
## Chain 1:   Stan can't start sampling from this initial value.
## Chain 1:
## Chain 1: Gradient evaluation took 0.000106 seconds
## Chain 1: 1000 transitions using 10 leapfrog steps per transition would take 1.06 seconds.
## Chain 1: Adjust your expectations accordingly!
## Chain 1:
## Chain 1:
## Chain 1: Iteration:    1 / 3000 [  0%]  (Warmup)
## Chain 1: Iteration:  300 / 3000 [ 10%]  (Warmup)
## Chain 1: Iteration:  600 / 3000 [ 20%]  (Warmup)
## Chain 1: Iteration:  900 / 3000 [ 30%]  (Warmup)
## Chain 1: Iteration: 1200 / 3000 [ 40%]  (Warmup)
## Chain 1: Iteration: 1500 / 3000 [ 50%]  (Warmup)
## Chain 1: Iteration: 1800 / 3000 [ 60%]  (Warmup)
## Chain 1: Iteration: 2001 / 3000 [ 66%]  (Sampling)
## Chain 1: Iteration: 2300 / 3000 [ 76%]  (Sampling)
## Chain 1: Iteration: 2600 / 3000 [ 86%]  (Sampling)
## Chain 1: Iteration: 2900 / 3000 [ 96%]  (Sampling)
## Chain 1: Iteration: 3000 / 3000 [100%]  (Sampling)
## Chain 1:
## Chain 1:  Elapsed Time: 4.06989 seconds (Warm-up)
## Chain 1:                1.65888 seconds (Sampling)
```

```
## Chain 1:                5.72877 seconds (Total)
## Chain 1:
##
## SAMPLING FOR MODEL 'YWP' NOW (CHAIN 2).
## Chain 2: Rejecting initial value:
## Chain 2:   Log probability evaluates to log(0), i.e. negative infinity.
## Chain 2:   Stan can't start sampling from this initial value.
## Chain 2: Rejecting initial value:
## Chain 2:   Log probability evaluates to log(0), i.e. negative infinity.
## Chain 2:   Stan can't start sampling from this initial value.
## Chain 2: Rejecting initial value:
## Chain 2:   Log probability evaluates to log(0), i.e. negative infinity.
## Chain 2:   Stan can't start sampling from this initial value.
## Chain 2: Rejecting initial value:
## Chain 2:   Log probability evaluates to log(0), i.e. negative infinity.
## Chain 2:   Stan can't start sampling from this initial value.
## Chain 2: Rejecting initial value:
## Chain 2:   Log probability evaluates to log(0), i.e. negative infinity.
## Chain 2:   Stan can't start sampling from this initial value.
## Chain 2: Rejecting initial value:
## Chain 2:   Log probability evaluates to log(0), i.e. negative infinity.
## Chain 2:   Stan can't start sampling from this initial value.
## Chain 2: Rejecting initial value:
## Chain 2:   Log probability evaluates to log(0), i.e. negative infinity.
## Chain 2:   Stan can't start sampling from this initial value.
## Chain 2: Rejecting initial value:
## Chain 2:   Log probability evaluates to log(0), i.e. negative infinity.
## Chain 2:   Stan can't start sampling from this initial value.
## Chain 2: Rejecting initial value:
## Chain 2:   Log probability evaluates to log(0), i.e. negative infinity.
## Chain 2:   Stan can't start sampling from this initial value.
## Chain 2: Rejecting initial value:
## Chain 2:   Log probability evaluates to log(0), i.e. negative infinity.
## Chain 2:   Stan can't start sampling from this initial value.
## Chain 2: Rejecting initial value:
## Chain 2:   Log probability evaluates to log(0), i.e. negative infinity.
## Chain 2:   Stan can't start sampling from this initial value.
## Chain 2: Rejecting initial value:
## Chain 2:   Log probability evaluates to log(0), i.e. negative infinity.
## Chain 2:   Stan can't start sampling from this initial value.
## Chain 2: Rejecting initial value:
## Chain 2:   Log probability evaluates to log(0), i.e. negative infinity.
## Chain 2:   Stan can't start sampling from this initial value.
## Chain 2: Rejecting initial value:
## Chain 2:   Log probability evaluates to log(0), i.e. negative infinity.
## Chain 2:   Stan can't start sampling from this initial value.
## Chain 2: Rejecting initial value:
## Chain 2:   Log probability evaluates to log(0), i.e. negative infinity.
## Chain 2:   Stan can't start sampling from this initial value.
## Chain 2: Rejecting initial value:
## Chain 2:   Log probability evaluates to log(0), i.e. negative infinity.
## Chain 2:   Stan can't start sampling from this initial value.
## Chain 2: Rejecting initial value:
## Chain 2:   Log probability evaluates to log(0), i.e. negative infinity.
```

```
## Chain 2:   Stan can't start sampling from this initial value.
## Chain 2: Rejecting initial value:
## Chain 2:   Log probability evaluates to log(0), i.e. negative infinity.
## Chain 2:   Stan can't start sampling from this initial value.
## Chain 2: Rejecting initial value:
## Chain 2:   Log probability evaluates to log(0), i.e. negative infinity.
## Chain 2:   Stan can't start sampling from this initial value.
## Chain 2: Rejecting initial value:
## Chain 2:   Log probability evaluates to log(0), i.e. negative infinity.
## Chain 2:   Stan can't start sampling from this initial value.
## Chain 2: Rejecting initial value:
## Chain 2:   Log probability evaluates to log(0), i.e. negative infinity.
## Chain 2:   Stan can't start sampling from this initial value.
## Chain 2: Rejecting initial value:
## Chain 2:   Log probability evaluates to log(0), i.e. negative infinity.
## Chain 2:   Stan can't start sampling from this initial value.
## Chain 2: Rejecting initial value:
## Chain 2:   Log probability evaluates to log(0), i.e. negative infinity.
## Chain 2:   Stan can't start sampling from this initial value.
## Chain 2: Rejecting initial value:
## Chain 2:   Log probability evaluates to log(0), i.e. negative infinity.
## Chain 2:   Stan can't start sampling from this initial value.
## Chain 2: Rejecting initial value:
## Chain 2:   Log probability evaluates to log(0), i.e. negative infinity.
## Chain 2:   Stan can't start sampling from this initial value.
## Chain 2: Rejecting initial value:
## Chain 2:   Log probability evaluates to log(0), i.e. negative infinity.
## Chain 2:   Stan can't start sampling from this initial value.
## Chain 2:
## Chain 2: Gradient evaluation took 7.2e-05 seconds
## Chain 2: 1000 transitions using 10 leapfrog steps per transition would take 0.72 seconds.
## Chain 2: Adjust your expectations accordingly!
## Chain 2:
## Chain 2:
## Chain 2: Iteration:    1 / 3000 [  0%]  (Warmup)
## Chain 2: Iteration:  300 / 3000 [ 10%]  (Warmup)
## Chain 2: Iteration:  600 / 3000 [ 20%]  (Warmup)
## Chain 2: Iteration:  900 / 3000 [ 30%]  (Warmup)
## Chain 2: Iteration: 1200 / 3000 [ 40%]  (Warmup)
## Chain 2: Iteration: 1500 / 3000 [ 50%]  (Warmup)
## Chain 2: Iteration: 1800 / 3000 [ 60%]  (Warmup)
## Chain 2: Iteration: 2001 / 3000 [ 66%]  (Sampling)
## Chain 2: Iteration: 2300 / 3000 [ 76%]  (Sampling)
## Chain 2: Iteration: 2600 / 3000 [ 86%]  (Sampling)
## Chain 2: Iteration: 2900 / 3000 [ 96%]  (Sampling)
## Chain 2: Iteration: 3000 / 3000 [100%]  (Sampling)
## Chain 2:
## Chain 2:  Elapsed Time: 3.77131 seconds (Warm-up)
## Chain 2:                1.46506 seconds (Sampling)
## Chain 2:                5.23637 seconds (Total)
## Chain 2:
##
## SAMPLING FOR MODEL 'YWP' NOW (CHAIN 3).
## Chain 3: Rejecting initial value:
```

```
## Chain 3:   Log probability evaluates to log(0), i.e. negative infinity.
## Chain 3:   Stan can't start sampling from this initial value.
## Chain 3: Rejecting initial value:
## Chain 3:   Log probability evaluates to log(0), i.e. negative infinity.
## Chain 3:   Stan can't start sampling from this initial value.
## Chain 3:
## Chain 3: Gradient evaluation took 5.3e-05 seconds
## Chain 3: 1000 transitions using 10 leapfrog steps per transition would take 0.53 seconds.
## Chain 3: Adjust your expectations accordingly!
## Chain 3:
## Chain 3:
## Chain 3: Iteration:    1 / 3000 [  0%]  (Warmup)
## Chain 3: Iteration:  300 / 3000 [ 10%]  (Warmup)
## Chain 3: Iteration:  600 / 3000 [ 20%]  (Warmup)
## Chain 3: Iteration:  900 / 3000 [ 30%]  (Warmup)
## Chain 3: Iteration: 1200 / 3000 [ 40%]  (Warmup)
## Chain 3: Iteration: 1500 / 3000 [ 50%]  (Warmup)
## Chain 3: Iteration: 1800 / 3000 [ 60%]  (Warmup)
## Chain 3: Iteration: 2001 / 3000 [ 66%]  (Sampling)
## Chain 3: Iteration: 2300 / 3000 [ 76%]  (Sampling)
## Chain 3: Iteration: 2600 / 3000 [ 86%]  (Sampling)
## Chain 3: Iteration: 2900 / 3000 [ 96%]  (Sampling)
## Chain 3: Iteration: 3000 / 3000 [100%]  (Sampling)
## Chain 3:
## Chain 3:  Elapsed Time: 3.76002 seconds (Warm-up)
## Chain 3:                1.36744 seconds (Sampling)
## Chain 3:                5.12746 seconds (Total)
## Chain 3:
```

Chain convergence

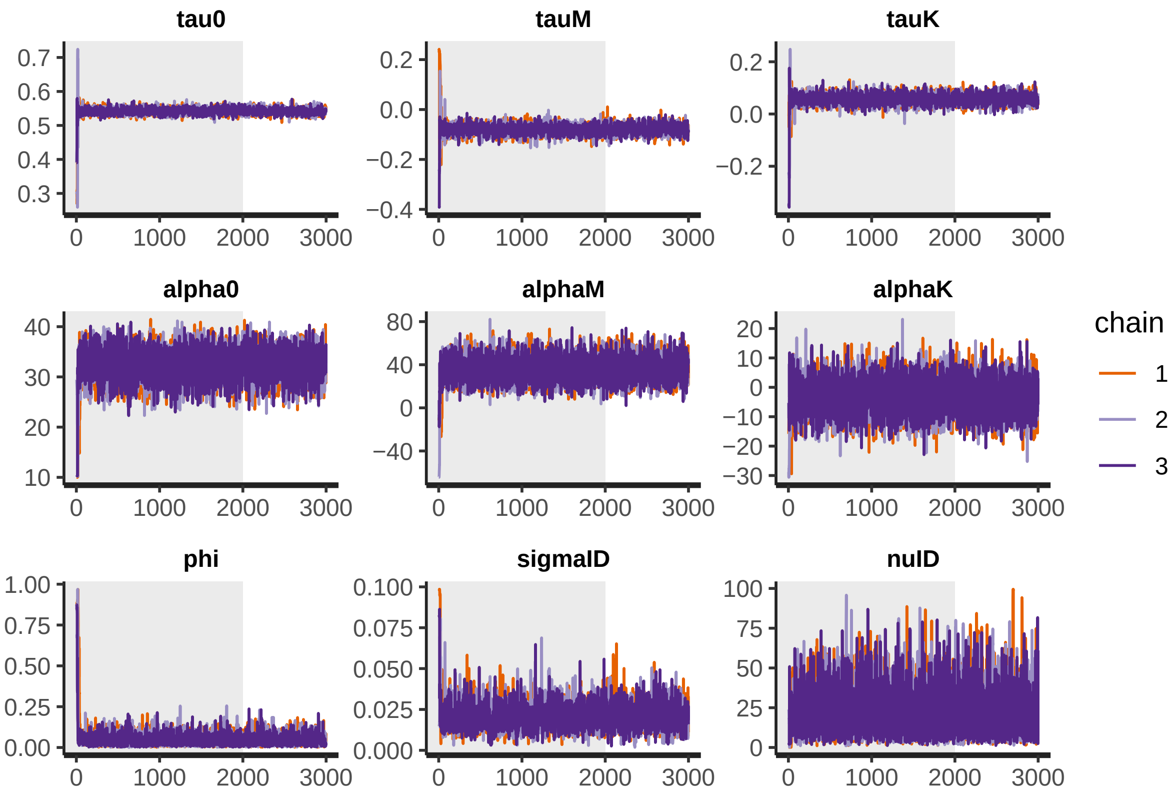

Figure 2: MCMC chain dynamics.

The chains appear to have converged.

Parameter correlations

```
mcmc_pairs(fit,
    pars = c("tau0", "tauM", "tauK", "alpha0"),
    off_diag_fun = "hex"
)
```

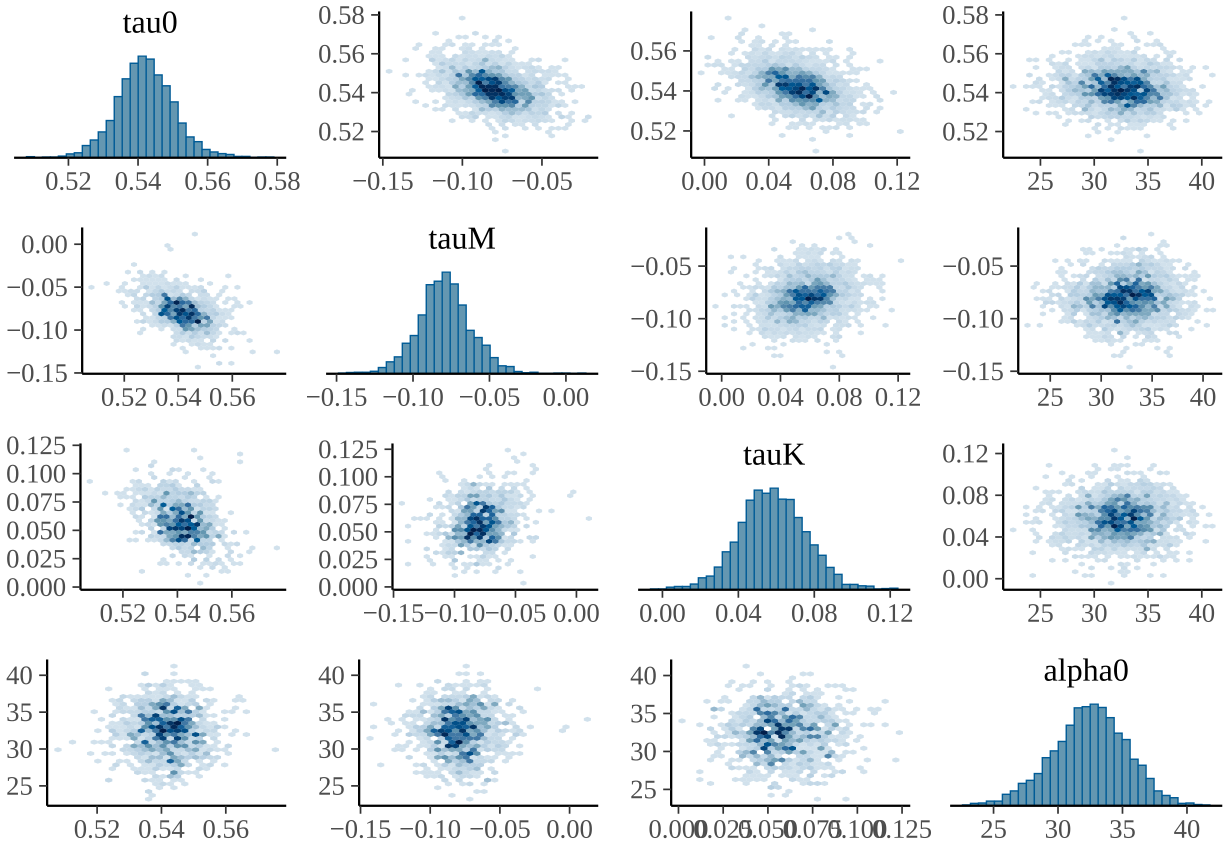


```r
mcmc_pairs(fit,
    pars = c("tau0", "alpha0", "alphaM", "alphaK"),
    off_diag_fun = "hex"
)
```

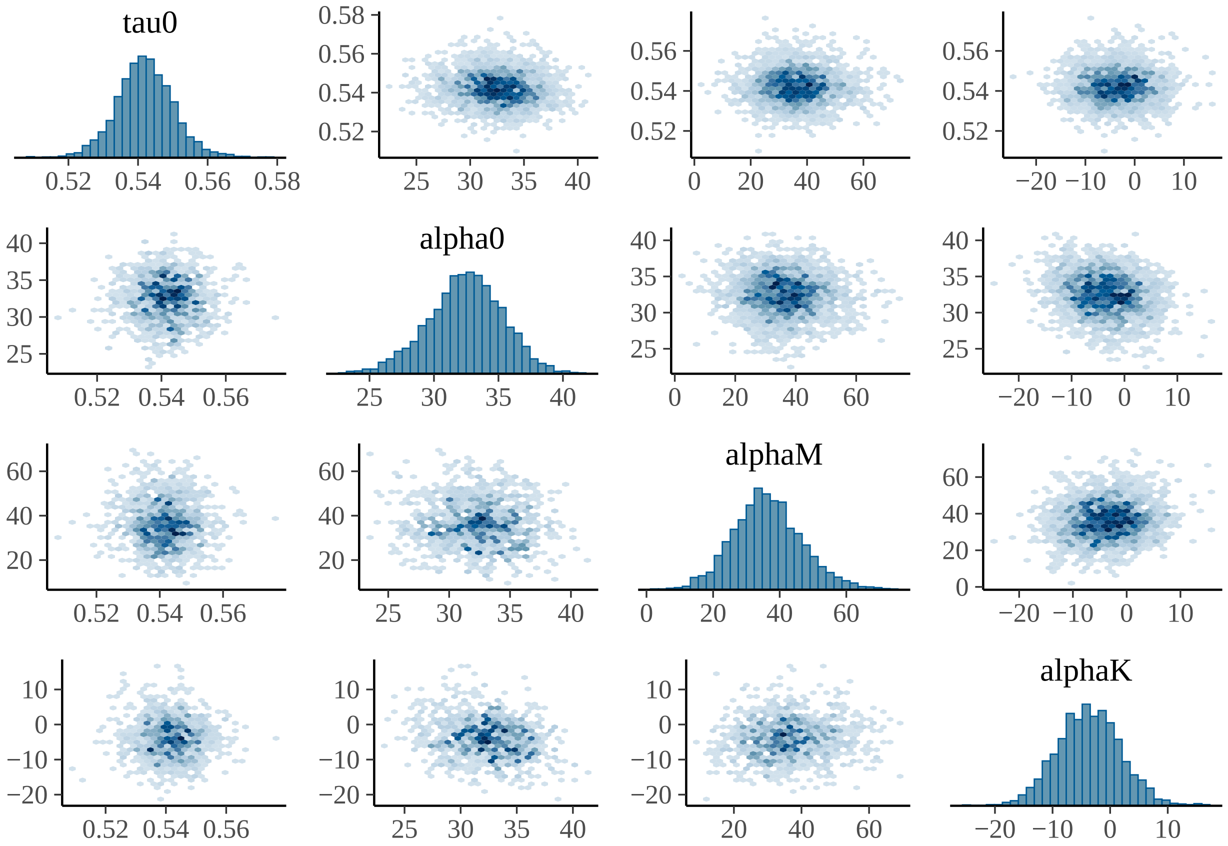

Figure 3: Correlations among parameters associated with change in the day of 50% shed (tau parameters) and the rate of shedding (alpha parameters).

Parameters are not highly correlated.

Parameter estimates

```
## Inference for Stan model: YWP.
## 3 chains, each with iter=3000; warmup=2000; thin=1;
## post-warmup draws per chain=1000, total post-warmup draws=3000.
##
##            mean se_mean     sd    5.5%    50%  94.5% n_eff  Rhat
## tau0      0.542   0.000  0.008   0.529  0.542  0.555   956 1.006
## tauM     -0.079   0.000  0.017  -0.106 -0.080 -0.051  1385 1.003
## tauK      0.058   0.000  0.017   0.031  0.058  0.086  1555 1.001
## alpha0   32.453   0.081  2.856  27.595 32.528 36.852  1241 1.001
## alphaM   36.411   0.203 10.245  21.050 35.858 53.424  2544 0.999
## alphaK   -3.672   0.105  5.438 -12.225 -3.734  5.144  2682 1.000
## phi       0.046   0.001  0.031   0.009  0.039  0.101   866 1.000
## sigmaID  0.020   0.000  0.007   0.009  0.019  0.032   727 1.000
## nuID     21.814   0.345 14.843   5.013 18.356 48.797  1850 1.001
##
## Samples were drawn using NUTS(diag_e) at Sun Sep 13 07:22:26 2020.
## For each parameter, n_eff is a crude measure of effective sample size,
## and Rhat is the potential scale reduction factor on split chains (at
## convergence, Rhat=1).
```

```r
mcmc_intervals(fit, pars = c("tauM", "tauK"), prob_outer = 0.89)
```

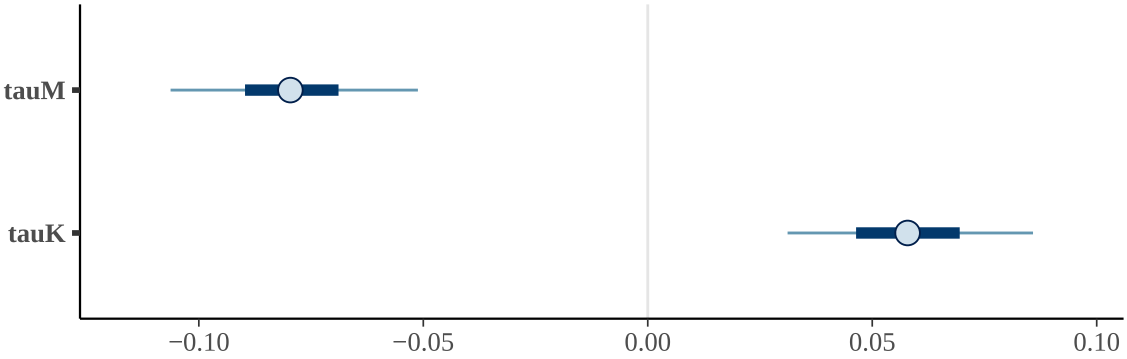


```r
mcmc_intervals(fit, pars = c("alphaM", "alphaK"), prob_outer = 0.89)
```

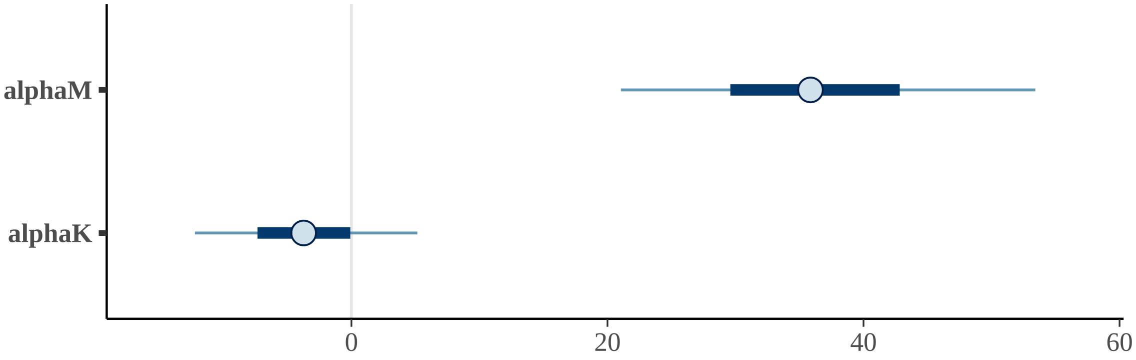

Figure 4: 89% credible intervals for state-dependent changes in shedding date (tau parameters) and shedding rate (alpha parameters).

Individual variation

```r
model_params <- "ID_tau_RE"
id <- levels(factor(df_fit$Individual_ID))

l_params <- rstan::extract(fit, pars = model_params)
m_RE <- l_params$ID_tau_RE
colnames(m_RE) <- as.character(id)
df_RE <- data.frame(m_RE)
names(df_RE) <- as.character(id)

df_RE$Rep <- 1:nrow(m_RE)
df_RE <- gather(df_RE, key = ID, value = RE, 1:length(id))
df_summary <- df_RE %>%
  group_by(ID) %>%
  summarise( # 89% credible bounds
    low89  = 365*quantile(RE, probs = 0.055),
    median = 365*quantile(RE, probs = 0.500),
    upp89  = 365*quantile(RE, probs = 0.945)
  )
```

```
## `summarise()` ungrouping output (override with `.groups` argument)
```

```
ggplot(df_summary, aes(x = ID, y = median))  +
  geom_hline(yintercept = 0, linetype = "dashed") +
  geom_point() +
  geom_errorbar(aes(ymin = low89, ymax = upp89), width = 0.2) +
  labs(y = "Change in\nmolting date (days)") +
  theme_bw() +
  theme(axis.text.x = element_text(angle = 30, hjust = 1, vjust=1))
```

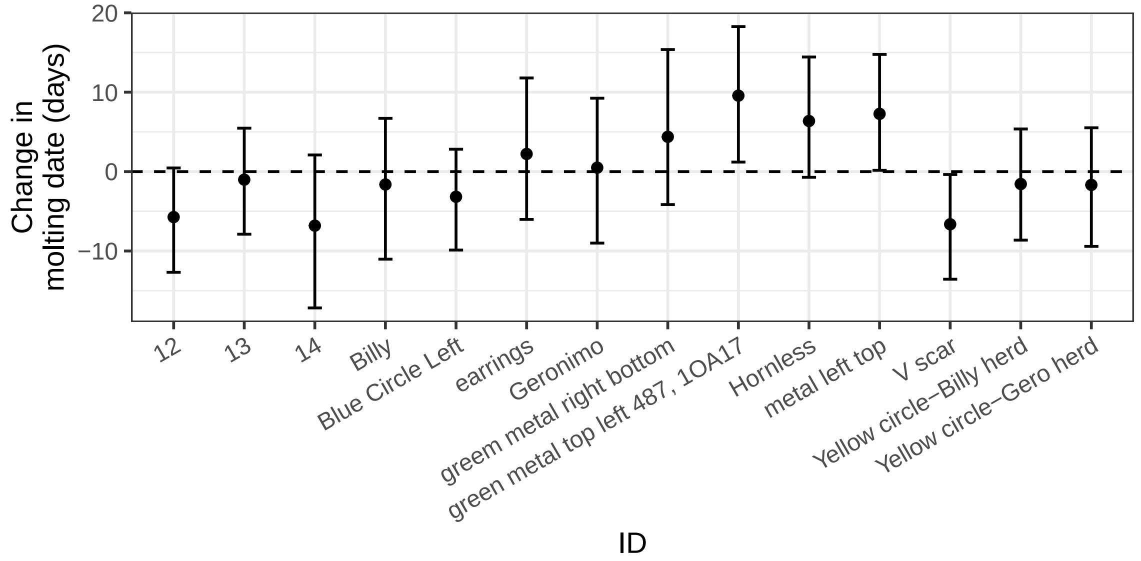

The deviations seem consistent with a t-distribution.

Model Predictions

```
l_params <- rstan::extract(fit, pars = c("tau0", "tauM", "tauK", "alpha0", "alphaM", "alphaK"))
df_params <- data.frame(l_params)
v_doy <- seq(from = min(df_YWP$doy), to = max(df_YWP$doy), by = 1)
min_doy <- min(df_YWP$doy)

df_FN <- data.frame(
    doy = v_doy,
    state = "FN",
    low89 = 0.0,
    medn  = 0.0,
    upp89 = 0.0
)

for (doy in v_doy) {
    frac_shed <- df_params$alpha0*(doy/365 - df_params$tau0)
    frac_shed <- exp(frac_shed) / (1.0  + exp(frac_shed))
    df_FN[doy + 1 - min_doy, 3:5] <- quantile(frac_shed,  probs = c(0.055, 0.5, 0.945))
}
```

```r
df_FY <- data.frame(
    doy = v_doy,
    state = "FY",
    low89 = 0.0,
    medn  = 0.0,
    upp89 = 0.0
)

for (doy in v_doy) {
    frac_shed <- (df_params$alpha0 + df_params$alphaK)*(doy/365 - df_params$tau0 - df_params$tauK)
    frac_shed <- exp(frac_shed) / (1.0  + exp(frac_shed))
    df_FY[doy + 1 - min_doy, 3:5] <- quantile(frac_shed,  probs = c(0.055, 0.5, 0.945))
}

df_MN <- data.frame(
    doy = v_doy,
    state = "MN",
    low89 = 0.0,
    medn  = 0.0,
    upp89 = 0.0
)

for (doy in v_doy) {
    frac_shed <- (df_params$alpha0 + df_params$alphaM)*(doy/365 - df_params$tau0 - df_params$tauM)
    frac_shed <- exp(frac_shed) / (1.0  + exp(frac_shed))
    df_MN[doy + 1 - min_doy, 3:5] <- quantile(frac_shed,  probs = c(0.055, 0.5, 0.945))
}

df_CI <- rbind(df_FN, df_FY, df_MN)
```

```r
df_YWP$SK <- factor(df_YWP$SK)
levels(df_YWP$SK)[levels(df_YWP$SK)=="FN"] <- "FN (female, no kid)"
levels(df_YWP$SK)[levels(df_YWP$SK)=="FY"] <- "FY (female with kid)"
levels(df_YWP$SK)[levels(df_YWP$SK)=="MN"] <- "MN (male, no kid)"

df_CI$state <- factor(df_CI$state)
levels(df_CI$state)[levels(df_CI$state)=="FN"] <- "FN (female, no kid)"
levels(df_CI$state)[levels(df_CI$state)=="FY"] <- "FY (female with kid)"
levels(df_CI$state)[levels(df_CI$state)=="MN"] <- "MN (male, no kid)"

ggplot(df_CI) +
    geom_ribbon(aes(x = doy, ymin =  low89, ymax = upp89, fill = state),
        alpha = 0.5) +
  geom_line(aes(x = doy, y = medn, color = state)) +
    geom_line(data = df_YWP,
        aes(x = doy, y = percent_shed, color = SK, group = Individual_ID)) +
  geom_point(data = df_YWP,
    aes(x = doy, y = percent_shed, color = SK)) +
    scale_colour_manual(values=c("#980043", "#e7298a", "blue")) +
    scale_fill_manual(values=c("#980043", "#e7298a", "blue")) +
    theme_bw() +
    xlim(140,260) +
    #scale_x_continuous(breaks=seq(from = 120, to = 270, by = 20)) +
```

```
  labs(
    x = "Day of year",
    y = "Fraction shed",
    color = "Animal state") +
    guides(fill = FALSE) +
    theme(
    legend.position=c(0.99,0.05),
    legend.justification=c(1,0),
    legend.text = element_text(size=8),
    panel.grid.minor = element_blank()
  )
```

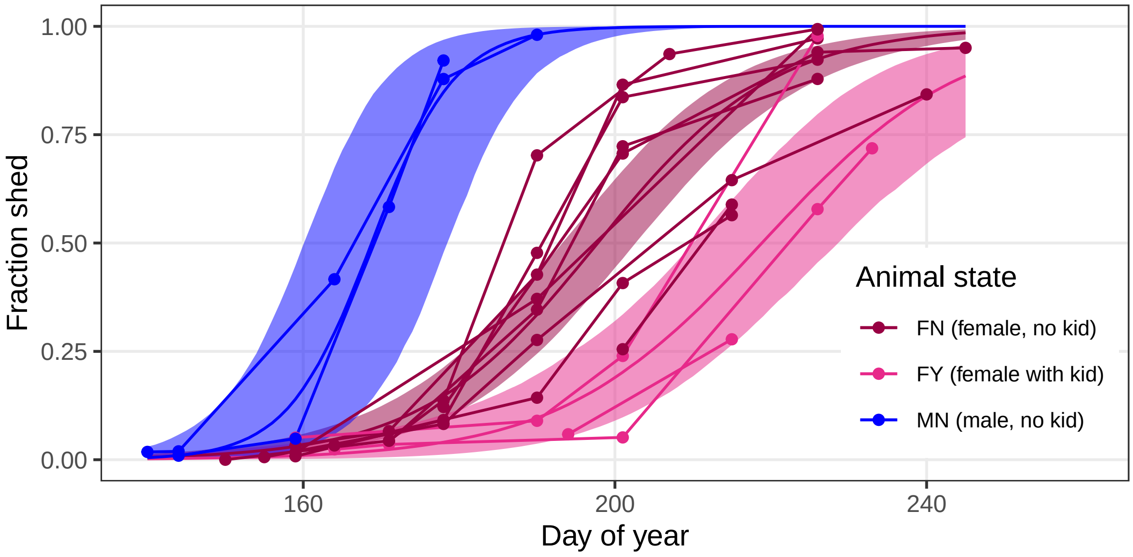

Figure 5: Median and 89% credible intervals associated with the predicted shedding fractions for the three animal states.

Stan code

Stan code used to describe the shedding model.

```
writeLines(readLines("YWP.stan"))
```

```
## // YWP.stan
## data {
##   int<lower=1>                 I;     // Number of animals
##   int<lower=1>                 N;     // Number of shedding blocks
##   int<lower=1>                 J;     // Number of distinct animals
##   int<lower=1,upper=3>     State[I];  // Animal state: FN, FY, MN
##   real<lower=-5.0,upper=5.0> zt[I];  // z-day of year [0,1]
##   int<lower=0,upper=J>       id[I];  // individual index [1,J]
##   int<lower=0,upper=N>        n[I];  // number of shed blocks [0,N]
## }
##
## parameters {
```

```
##   real <lower=0.2,upper=0.8>   tau0;    // shedding date
##   real <lower=10,upper=50>     alpha0;  // shedding rate
##   real <lower=-0.5,upper=0.5>  tauM;    // male effect on shedding day
##   real <lower=-0.5,upper=0.5>  tauK;    // kid effect on shedding day
##   real <lower=-100,upper=100>  alphaM;  // change in male shedding rate
##   real <lower=-100,upper=100>  alphaK;  // change in kid shedding rate
##   real <lower=0.001,upper=0.1> sigmaID; // ID-ID random variation (shedding day)
##   real <lower=0.1,upper=100.0> nuID;    // ID-ID random variation (shedding day)
##   real <lower=0.001,upper=1.0> phi;     // beta-binomial variation term
##
##   real ID_tau_RE[J];   // estimated ID variation (random effect on alpha)
## }
##
## model {
##   real logit_FN;
##   real logit_FY;
##   real logit_MN;
##   real mu;
##   real a;
##   real b;
##   int  y;
##   real tau_i;
##   real alpha_i;
##
##   tau0    ~ beta(2,2);
##   alpha0  ~ normal(25,5);
##   tauM    ~ normal(0,0.1);
##   tauK    ~ normal(0,0.1);
##   alphaK  ~ normal(0,20);
##   alphaM  ~ normal(0,20);
##   sigmaID ~ exponential(10.0);
##   nuID    ~ gamma(2, 0.1);
##   phi     ~ exponential(1.0);
##
##   # ID_tau_RE ~ normal(0, sigmaID); // ID random effect
##   ID_tau_RE ~ student_t(nuID, 0.0, sigmaID);
##
##   for (i in 1:I) {
##     tau_i = tau0 + ID_tau_RE[id[i]];
##     if (State[i] == 1) { // FN
##       logit_FN = alpha0*(zt[i] - tau_i);
##       mu = exp(logit_FN) / (1.0 + exp(logit_FN));
##       a = mu / phi;
##       b = (1.0 - mu) / phi;
##       y = n[i];
##       target += lgamma(N+1) + lgamma(a+b) + lgamma(y+a) +
##         lgamma(N-y+b) - lgamma(y+1) - lgamma(N-y+1) -
##         lgamma(a) - lgamma(b) - lgamma(N+a+b);
##     } else if (State[i] == 2) { // FY
##       logit_FY = (alpha0 + alphaK)*(zt[i] - tau_i - tauK);
##       mu = exp(logit_FY) / (1.0 + exp(logit_FY));
##       a = mu / phi;
##       b = (1.0 - mu) / phi;
##       y = n[i];
```

```
##       target += lgamma(N+1) + lgamma(a+b) + lgamma(y+a) +
##         lgamma(N-y+b) - lgamma(y+1) - lgamma(N-y+1) -
##         lgamma(a) - lgamma(b) - lgamma(N+a+b);
##     } else if (State[i] == 3) { // MN
##       logit_MN = (alpha0 + alphaM)*(zt[i] - tau_i - tauM);
##       mu = exp(logit_MN) / (1.0 + exp(logit_MN));
##       a = mu / phi;
##       b = (1.0 - mu) / phi;
##       y = n[i];
##       target += lgamma(N+1) + lgamma(a+b) + lgamma(y+a) +
##         lgamma(N-y+b) - lgamma(y+1) - lgamma(N-y+1) -
##         lgamma(a) - lgamma(b) - lgamma(N+a+b);
##     }
##   }
## }
```
