# Supplementary material for: Using community photography to investigate phenology: A case study of coat molt in the mountain goat (Oreamnos americanus) with missing data
Source: Ecol Evol. 2020 Nov 9;10(23):13488–99. doi: 10.1002/ece3.6954 (PMC7713987; doi:10.1002/ece3.6954)
Supplement: Supplementary file 4 — Supinfo4 [file ECE3-10-13488-s004.pdf]

Supplementary Materials 4: Community-Sourced Data Analysis (Ignore Incomplete Data)

Shane A. Richards

2020-09-13

Contents

Summary **1**

Data **2**

Chain convergence 7

Parameter correlations 8

Parameter estimates 9

Parameter Credibility 10

Random effects 11

Predictions **13**

Long-term trend 13

Within-season 15

Stan code **18**

Summary

This document supports the manuscript:

> Nowak et al. "Using community photography to investigate phenology: a case study of coat molt in the mountain goat (Oreamnos americanus) with missing data" submitted to Ecology and Evolution.

Here we present the statistical analysis of the community-sourced data set, like presented in Supplementary Materials 2', however this analysis only incorporates photos where animal state is never ambiguous.

This document is near identical to Supplementary Materials 2, except we first filter out the ambiguous states. The stan code is slightly modified as we now do not need to estimate the parameters p and q (i.e. the fraction of photos that are of of females and the fraction of those where the kid is present).

Data

```
rm(list = ls()) # clear memory

# load all packages needed for the analysis
library(tidyverse)
library(readxl)
library(lubridate)
library(rstan)
library(cowplot)
library(scales)
library(ggmap)
library(bayesplot)

# read in the data
df_fit <- read_csv("../Data Wrangling/CitizenScienceWrangled.csv")

SHED <- 25 # bins that define molting state

# add variables for fitting
df_fit <- df_fit %>%
  mutate(shed = as.integer(round(SHED*frac_shed, 0)))

# create appropriate factors
df_fit$Sex  <- factor(df_fit$Sex)
df_fit$Sex  <- fct_relevel(df_fit$Sex,  "F", "M", "X")
df_fit$Kids <- factor(df_fit$Kids)
df_fit$Kids <- fct_relevel(df_fit$Kids, "N", "Y", "X")
df_fit$SK <- factor(df_fit$SK)
df_fit$SK <- fct_relevel(df_fit$SK, "FN", "FY", "FX", "MN", "XN", "XX")

df_fit <- arrange(df_fit, DateObs, SK, Lat)
```

```
# prepare data for fitting with stan (i.e. z-transformations of predictors)
Lat_mu <- mean(df_fit$Lat)
Lat_sd <- sd(df_fit$Lat)

Elv_mu <- mean(df_fit$ElevGIS)
Elv_sd <- sd(df_fit$ElevGIS)

doy_mu <- mean(df_fit$doy)
doy_sd <- sd(df_fit$doy)

yr_mu <- mean(df_fit$year)
yr_sd <- sd(df_fit$year)

df_fit$yr_F  <- factor(df_fit$year) # create a factor version of year
df_fit$fYear <- as.integer(factor(df_fit$year))

# z-transform predictors and set 0 < DOY < 1
df_fit$z_yr  <- (df_fit$year - yr_mu) / yr_sd
df_fit$z_doy  <- df_fit$doy / 365
```

```
df_fit$z_lat <- (df_fit$Lat - Lat_mu) / Lat_sd
df_fit$z_ele <- (df_fit$ElevGIS - Elv_mu) / Elv_sd
```

Filter out photos where animal state is ambiguous.

```
df_fit <- df_fit %>%
    filter(State %in% c(1,2,4)) # only consider photos where state is known

model_params <- c(
  "tau0",   "tauM",   "tauK",   "tauY",   "tauE",   "tauL",
  "alpha0", "alphaM", "alphaK", "alphaY", "alphaE", "alphaL",
  "phi", "sigmaTau", "sigmaAlpha", "nuTau", "nuAlpha")

stan_dat <- list(
  I     = nrow(df_fit), # number of observations
  N     = SHED,         # molting categories
  Y     = max(df_fit$fYear), # number of years
  State = df_fit$State, # animal state (1,2, 4)
  zt    = df_fit$z_doy, # transformed day of year
  ze    = df_fit$z_ele, # z-transformed elevation
  zl    = df_fit$z_lat, # z-transformed latitude
  zy    = df_fit$z_yr,  # z-transformed year
  iy    = df_fit$fYear, # year index
  n     = df_fit$shed   # observed shed (0-N)
)
```

```
# fit the model!
fit <- stan(file = 'CSfitIncomplete.stan', data = stan_dat,
  iter = 2000, warmup = 1000, chains = 3, seed = 1971) # , refresh = 0)
```

```
## Warning in readLines(file, warn = TRUE): incomplete final line found on '/Users/
## shaner2/Documents/UTAS/Research/Projects/Mountain goat/Analysis/EvoEcolRevision/
## CitSci Incomplete/CSfitIncomplete.stan'
```

```
##
## SAMPLING FOR MODEL 'CSfitIncomplete' NOW (CHAIN 1).
## Chain 1: Rejecting initial value:
## Chain 1:   Log probability evaluates to log(0), i.e. negative infinity.
## Chain 1:   Stan can't start sampling from this initial value.
## Chain 1: Rejecting initial value:
## Chain 1:   Log probability evaluates to log(0), i.e. negative infinity.
## Chain 1:   Stan can't start sampling from this initial value.
## Chain 1: Rejecting initial value:
## Chain 1:   Log probability evaluates to log(0), i.e. negative infinity.
## Chain 1:   Stan can't start sampling from this initial value.
## Chain 1: Rejecting initial value:
## Chain 1:   Log probability evaluates to log(0), i.e. negative infinity.
## Chain 1:   Stan can't start sampling from this initial value.
## Chain 1: Rejecting initial value:
## Chain 1:   Log probability evaluates to log(0), i.e. negative infinity.
## Chain 1:   Stan can't start sampling from this initial value.
## Chain 1: Rejecting initial value:
```

```
## Chain 1:   Log probability evaluates to log(0), i.e. negative infinity.
## Chain 1:   Stan can't start sampling from this initial value.
## Chain 1: Rejecting initial value:
## Chain 1:   Log probability evaluates to log(0), i.e. negative infinity.
## Chain 1:   Stan can't start sampling from this initial value.
## Chain 1: Rejecting initial value:
## Chain 1:   Log probability evaluates to log(0), i.e. negative infinity.
## Chain 1:   Stan can't start sampling from this initial value.
## Chain 1: Rejecting initial value:
## Chain 1:   Log probability evaluates to log(0), i.e. negative infinity.
## Chain 1:   Stan can't start sampling from this initial value.
## Chain 1: Rejecting initial value:
## Chain 1:   Log probability evaluates to log(0), i.e. negative infinity.
## Chain 1:   Stan can't start sampling from this initial value.
## Chain 1: Rejecting initial value:
## Chain 1:   Log probability evaluates to log(0), i.e. negative infinity.
## Chain 1:   Stan can't start sampling from this initial value.
## Chain 1: Rejecting initial value:
## Chain 1:   Log probability evaluates to log(0), i.e. negative infinity.
## Chain 1:   Stan can't start sampling from this initial value.
## Chain 1: Rejecting initial value:
## Chain 1:   Log probability evaluates to log(0), i.e. negative infinity.
## Chain 1:   Stan can't start sampling from this initial value.
## Chain 1: Rejecting initial value:
## Chain 1:   Log probability evaluates to log(0), i.e. negative infinity.
## Chain 1:   Stan can't start sampling from this initial value.
## Chain 1: Rejecting initial value:
## Chain 1:   Log probability evaluates to log(0), i.e. negative infinity.
## Chain 1:   Stan can't start sampling from this initial value.
## Chain 1:
## Chain 1: Gradient evaluation took 0.000526 seconds
## Chain 1: 1000 transitions using 10 leapfrog steps per transition would take 5.26 seconds.
## Chain 1: Adjust your expectations accordingly!
## Chain 1:
## Chain 1:
## Chain 1: Iteration:    1 / 2000 [  0%]  (Warmup)
## Chain 1: Iteration:  200 / 2000 [ 10%]  (Warmup)
## Chain 1: Iteration:  400 / 2000 [ 20%]  (Warmup)
## Chain 1: Iteration:  600 / 2000 [ 30%]  (Warmup)
## Chain 1: Iteration:  800 / 2000 [ 40%]  (Warmup)
## Chain 1: Iteration: 1000 / 2000 [ 50%]  (Warmup)
## Chain 1: Iteration: 1001 / 2000 [ 50%]  (Sampling)
## Chain 1: Iteration: 1200 / 2000 [ 60%]  (Sampling)
## Chain 1: Iteration: 1400 / 2000 [ 70%]  (Sampling)
## Chain 1: Iteration: 1600 / 2000 [ 80%]  (Sampling)
## Chain 1: Iteration: 1800 / 2000 [ 90%]  (Sampling)
## Chain 1: Iteration: 2000 / 2000 [100%]  (Sampling)
## Chain 1:
## Chain 1:  Elapsed Time: 40.4937 seconds (Warm-up)
## Chain 1:                10.2156 seconds (Sampling)
## Chain 1:                50.7093 seconds (Total)
## Chain 1:
##
## SAMPLING FOR MODEL 'CSfitIncomplete' NOW (CHAIN 2).
```

```
## Chain 2: Rejecting initial value:
## Chain 2:   Log probability evaluates to log(0), i.e. negative infinity.
## Chain 2:   Stan can't start sampling from this initial value.
## Chain 2: Rejecting initial value:
## Chain 2:   Log probability evaluates to log(0), i.e. negative infinity.
## Chain 2:   Stan can't start sampling from this initial value.
## Chain 2: Rejecting initial value:
## Chain 2:   Log probability evaluates to log(0), i.e. negative infinity.
## Chain 2:   Stan can't start sampling from this initial value.
## Chain 2: Rejecting initial value:
## Chain 2:   Log probability evaluates to log(0), i.e. negative infinity.
## Chain 2:   Stan can't start sampling from this initial value.
## Chain 2:
## Chain 2: Gradient evaluation took 0.000311 seconds
## Chain 2: 1000 transitions using 10 leapfrog steps per transition would take 3.11 seconds.
## Chain 2: Adjust your expectations accordingly!
## Chain 2:
## Chain 2:
## Chain 2: Iteration:    1 / 2000 [  0%]  (Warmup)
## Chain 2: Iteration:  200 / 2000 [ 10%]  (Warmup)
## Chain 2: Iteration:  400 / 2000 [ 20%]  (Warmup)
## Chain 2: Iteration:  600 / 2000 [ 30%]  (Warmup)
## Chain 2: Iteration:  800 / 2000 [ 40%]  (Warmup)
## Chain 2: Iteration: 1000 / 2000 [ 50%]  (Warmup)
## Chain 2: Iteration: 1001 / 2000 [ 50%]  (Sampling)
## Chain 2: Iteration: 1200 / 2000 [ 60%]  (Sampling)
## Chain 2: Iteration: 1400 / 2000 [ 70%]  (Sampling)
## Chain 2: Iteration: 1600 / 2000 [ 80%]  (Sampling)
## Chain 2: Iteration: 1800 / 2000 [ 90%]  (Sampling)
## Chain 2: Iteration: 2000 / 2000 [100%]  (Sampling)
## Chain 2:
## Chain 2:  Elapsed Time: 36.242 seconds (Warm-up)
## Chain 2:                8.96155 seconds (Sampling)
## Chain 2:                45.2036 seconds (Total)
## Chain 2:
##
## SAMPLING FOR MODEL 'CSfitIncomplete' NOW (CHAIN 3).
## Chain 3: Rejecting initial value:
## Chain 3:   Log probability evaluates to log(0), i.e. negative infinity.
## Chain 3:   Stan can't start sampling from this initial value.
## Chain 3: Rejecting initial value:
## Chain 3:   Log probability evaluates to log(0), i.e. negative infinity.
## Chain 3:   Stan can't start sampling from this initial value.
## Chain 3: Rejecting initial value:
## Chain 3:   Log probability evaluates to log(0), i.e. negative infinity.
## Chain 3:   Stan can't start sampling from this initial value.
## Chain 3: Rejecting initial value:
## Chain 3:   Log probability evaluates to log(0), i.e. negative infinity.
## Chain 3:   Stan can't start sampling from this initial value.
## Chain 3: Rejecting initial value:
## Chain 3:   Log probability evaluates to log(0), i.e. negative infinity.
## Chain 3:   Stan can't start sampling from this initial value.
## Chain 3: Rejecting initial value:
## Chain 3:   Log probability evaluates to log(0), i.e. negative infinity.
```

```
## Chain 3:   Stan can't start sampling from this initial value.
## Chain 3: Rejecting initial value:
## Chain 3:   Log probability evaluates to log(0), i.e. negative infinity.
## Chain 3:   Stan can't start sampling from this initial value.
## Chain 3: Rejecting initial value:
## Chain 3:   Log probability evaluates to log(0), i.e. negative infinity.
## Chain 3:   Stan can't start sampling from this initial value.
## Chain 3: Rejecting initial value:
## Chain 3:   Log probability evaluates to log(0), i.e. negative infinity.
## Chain 3:   Stan can't start sampling from this initial value.
## Chain 3: Rejecting initial value:
## Chain 3:   Log probability evaluates to log(0), i.e. negative infinity.
## Chain 3:   Stan can't start sampling from this initial value.
## Chain 3: Rejecting initial value:
## Chain 3:   Log probability evaluates to log(0), i.e. negative infinity.
## Chain 3:   Stan can't start sampling from this initial value.
## Chain 3: Rejecting initial value:
## Chain 3:   Log probability evaluates to log(0), i.e. negative infinity.
## Chain 3:   Stan can't start sampling from this initial value.
## Chain 3: Rejecting initial value:
## Chain 3:   Log probability evaluates to log(0), i.e. negative infinity.
## Chain 3:   Stan can't start sampling from this initial value.
## Chain 3: Rejecting initial value:
## Chain 3:   Log probability evaluates to log(0), i.e. negative infinity.
## Chain 3:   Stan can't start sampling from this initial value.
## Chain 3: Rejecting initial value:
## Chain 3:   Log probability evaluates to log(0), i.e. negative infinity.
## Chain 3:   Stan can't start sampling from this initial value.
## Chain 3: Rejecting initial value:
## Chain 3:   Log probability evaluates to log(0), i.e. negative infinity.
## Chain 3:   Stan can't start sampling from this initial value.
## Chain 3:
## Chain 3: Gradient evaluation took 0.000856 seconds
## Chain 3: 1000 transitions using 10 leapfrog steps per transition would take 8.56 seconds.
## Chain 3: Adjust your expectations accordingly!
## Chain 3:
## Chain 3:
## Chain 3: Iteration:    1 / 2000 [  0%]  (Warmup)
## Chain 3: Iteration:  200 / 2000 [ 10%]  (Warmup)
## Chain 3: Iteration:  400 / 2000 [ 20%]  (Warmup)
## Chain 3: Iteration:  600 / 2000 [ 30%]  (Warmup)
## Chain 3: Iteration:  800 / 2000 [ 40%]  (Warmup)
## Chain 3: Iteration: 1000 / 2000 [ 50%]  (Warmup)
## Chain 3: Iteration: 1001 / 2000 [ 50%]  (Sampling)
## Chain 3: Iteration: 1200 / 2000 [ 60%]  (Sampling)
## Chain 3: Iteration: 1400 / 2000 [ 70%]  (Sampling)
## Chain 3: Iteration: 1600 / 2000 [ 80%]  (Sampling)
## Chain 3: Iteration: 1800 / 2000 [ 90%]  (Sampling)
## Chain 3: Iteration: 2000 / 2000 [100%]  (Sampling)
## Chain 3:
## Chain 3:  Elapsed Time: 39.4378 seconds (Warm-up)
## Chain 3:                10.1017 seconds (Sampling)
## Chain 3:                49.5396 seconds (Total)
## Chain 3:
```

```
## Warning: The largest R-hat is 1.1, indicating chains have not mixed.
## Running the chains for more iterations may help. See
## http://mc-stan.org/misc/warnings.html#r-hat

## Warning: Bulk Effective Samples Size (ESS) is too low, indicating posterior means and medians may be
## Running the chains for more iterations may help. See
## http://mc-stan.org/misc/warnings.html#bulk-ess

## Warning: Tail Effective Samples Size (ESS) is too low, indicating posterior variances and tail quant
## Running the chains for more iterations may help. See
## http://mc-stan.org/misc/warnings.html#tail-ess
```

Chain convergence

```r
model_params <- c(
  "tau0",   "tauM",   "tauK",   "tauY",   "tauE",   "tauL",
  "alpha0", "alphaM", "alphaK", "alphaY", "alphaE", "alphaL",
  "phi", "sigmaTau", "sigmaAlpha", "nuTau", "nuAlpha")

# check for chain convergence (can also set chains = 3 above)
traceplot(fit, pars = model_params, inc_warmup = TRUE, ncol = 4)
```

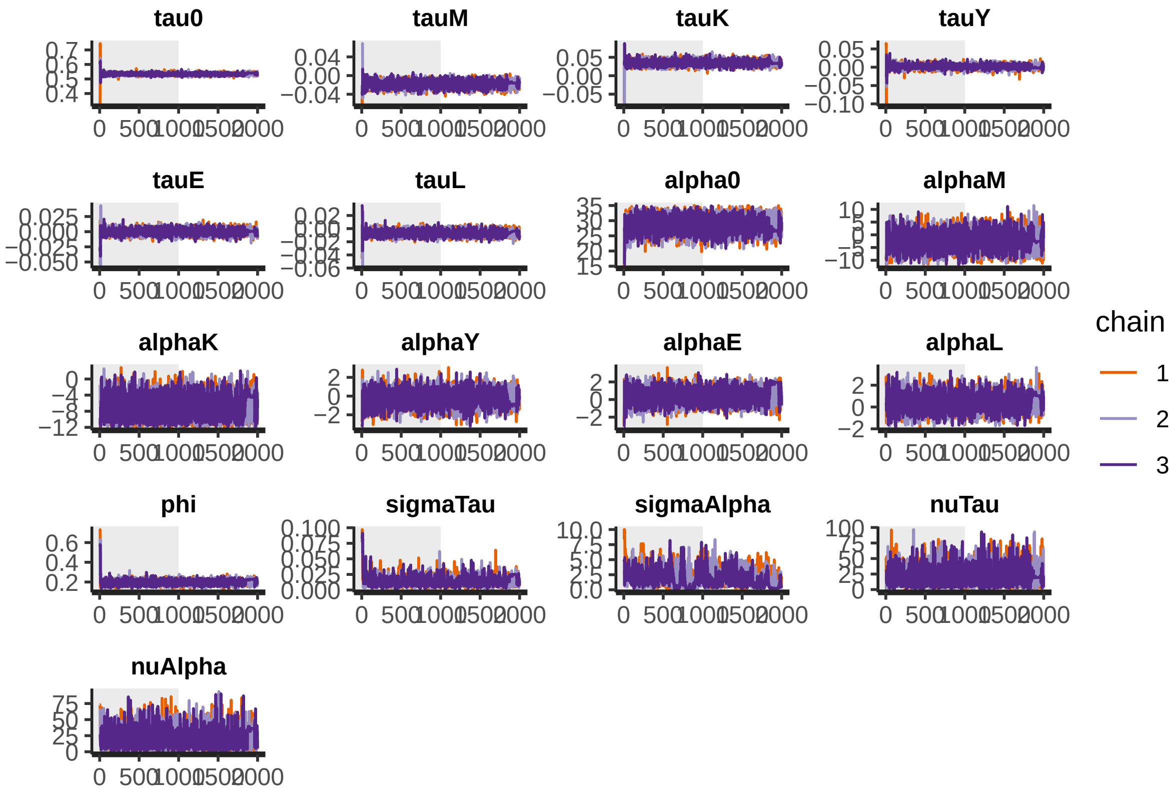

Figure 1: MCMC chain dynamics.

The chains have settled down after the 1000 burn-in.

Parameter correlations

```
mcmc_pairs(fit,
    pars = c("tau0", "tauM", "tauK", "tauY", "tauE", "tauL"),
  off_diag_fun = "hex"
)
```

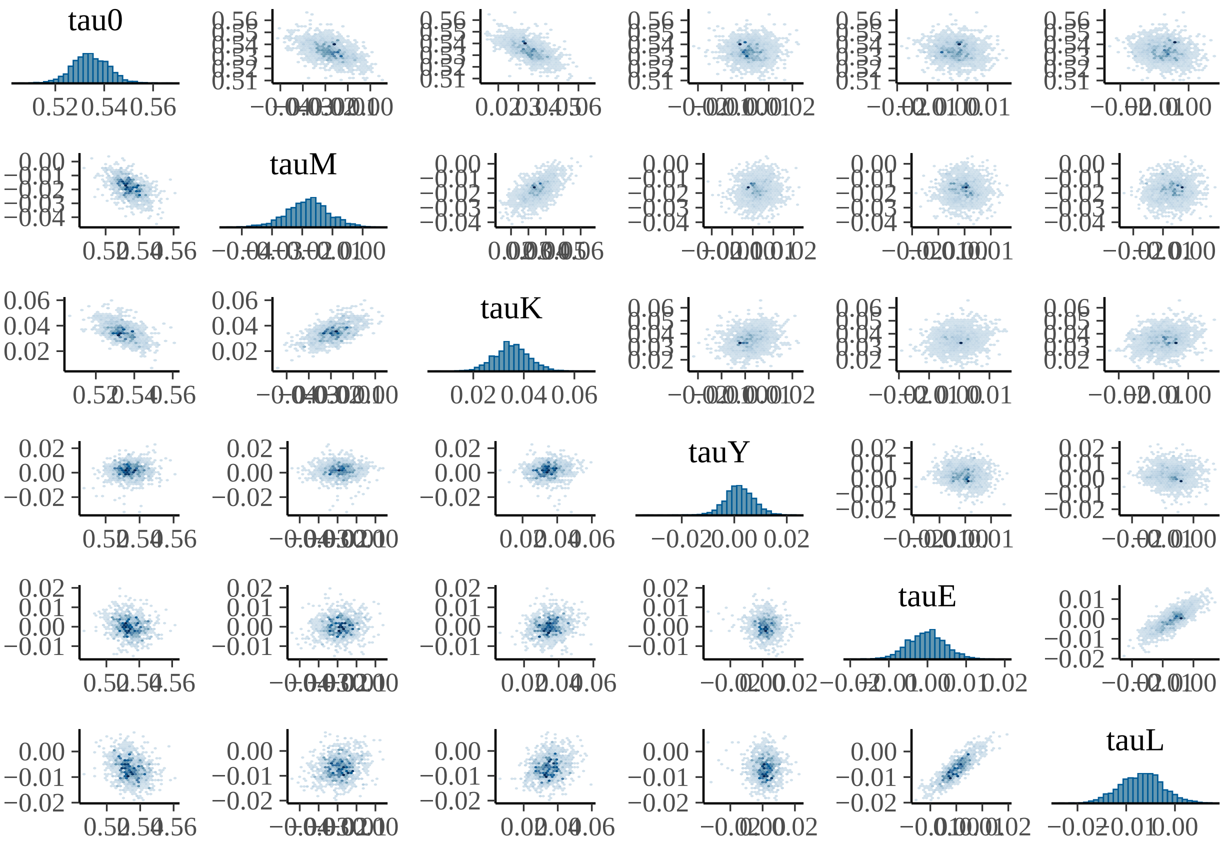


```
mcmc_pairs(fit,
    pars = c("alpha0", "alphaM", "alphaK", "alphaY", "alphaE", "alphaL"),
  off_diag_fun = "hex"
)
```

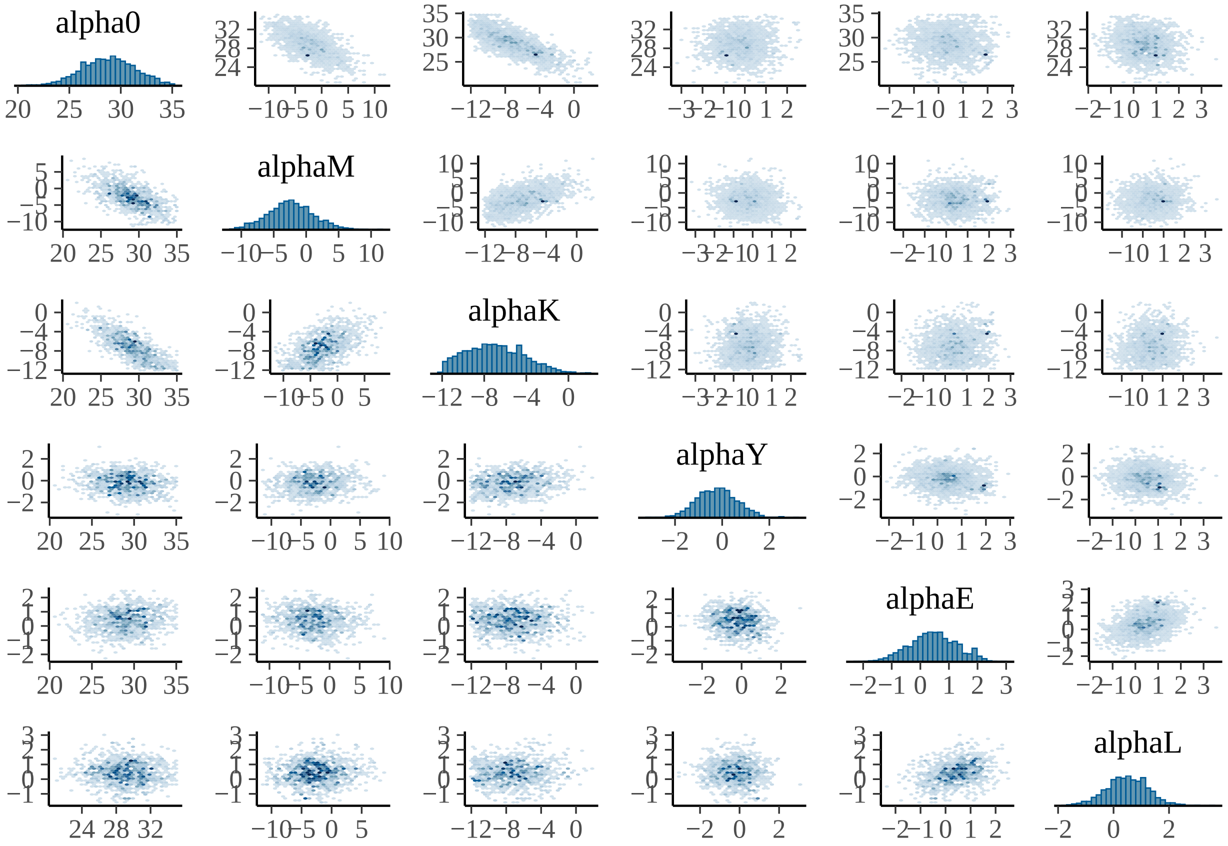

Figure 2: Correlations among parameters associated with change in the day of 50% shed (tau parameters) and the rate of shedding (alpha parameters).

Parameter estimates

Table 1: Parameter estimates and 89% credible intervals. Note that the scale of many parameters are different to those reported in the manuscript (e.g. tau here is fraction of year but days in the manuscript).

```r
model_params <- c(
  "tau0",   "tauM",   "tauK",   "tauY",   "tauE",   "tauL",
  "alpha0", "alphaM", "alphaK", "alphaY", "alphaE", "alphaL",
  "phi", "sigmaTau", "sigmaAlpha", "nuTau", "nuAlpha")

# display the posterior distribution statistics
print(fit, pars = model_params, probs = c(0.055,0.5,0.945), digits=3)
```

```
## Inference for Stan model: CSfitIncomplete.
## 3 chains, each with iter=2000; warmup=1000; thin=1;
## post-warmup draws per chain=1000, total post-warmup draws=3000.
##
##               mean se_mean    sd   5.5%    50%  94.5% n_eff  Rhat
## tau0         0.534   0.000 0.007  0.523  0.534  0.546  1172 1.001
## tauM        -0.018   0.000 0.007 -0.029 -0.018 -0.007  1518 1.007
## tauK         0.035   0.000 0.007  0.024  0.035  0.046  1601 1.001
## tauY         0.002   0.000 0.006 -0.007  0.002  0.011   805 1.009
## tauE         0.000   0.000 0.005 -0.008  0.000  0.008  1281 1.002
```

```
## tauL       -0.007   0.000  0.004  -0.014 -0.007  0.000   1094 1.001
## alpha0     28.817   0.096  2.555  24.704 28.845 32.962   707 1.007
## alphaM     -2.357   0.101  3.491  -8.042 -2.554  3.415   1188 1.004
## alphaK     -6.930   0.115  2.766 -11.094 -7.130 -2.299   581 1.008
## alphaY     -0.220   0.025  0.841  -1.528 -0.219  1.150   1171 1.004
## alphaE      0.460   0.053  0.821  -0.872  0.449  1.882   235 1.011
## alphaL      0.485   0.022  0.745  -0.726  0.496  1.635   1137 1.006
## phi         0.195   0.001  0.023   0.161  0.194  0.232   325 1.017
## sigmaTau    0.014   0.000  0.007   0.005  0.012  0.025   264 1.012
## sigmaAlpha  1.777   0.160  1.280   0.290  1.490  4.117    64 1.075
## nuTau      20.578   0.318 14.230   4.104 17.751 46.907  1998 1.003
## nuAlpha    18.958   0.523 13.906   2.541 16.138 43.810   708 1.002
##
## Samples were drawn using NUTS(diag_e) at Sun Sep 13 07:57:59 2020.
## For each parameter, n_eff is a crude measure of effective sample size,
## and Rhat is the potential scale reduction factor on split chains (at
## convergence, Rhat=1).
```

Males were estimated to be molting earlier than females and females with a kid were molting later ($\tau_\mathrm{M} < 0$, $\tau_\mathrm{K} > 0$). The model estimates molting to be occurring later at higher elevations ($\tau_\mathrm{E} > 0$).

There was no evidence that molting was related to elevation or latitude. However, there was some evidence that the rate of shedding was slower when females were associated with a kid.

Parameter Credibility

```
mcmc_intervals(fit, pars = c("tauM", "tauK", "tauY", "tauE", "tauL"),
    prob_outer = 0.89)
```

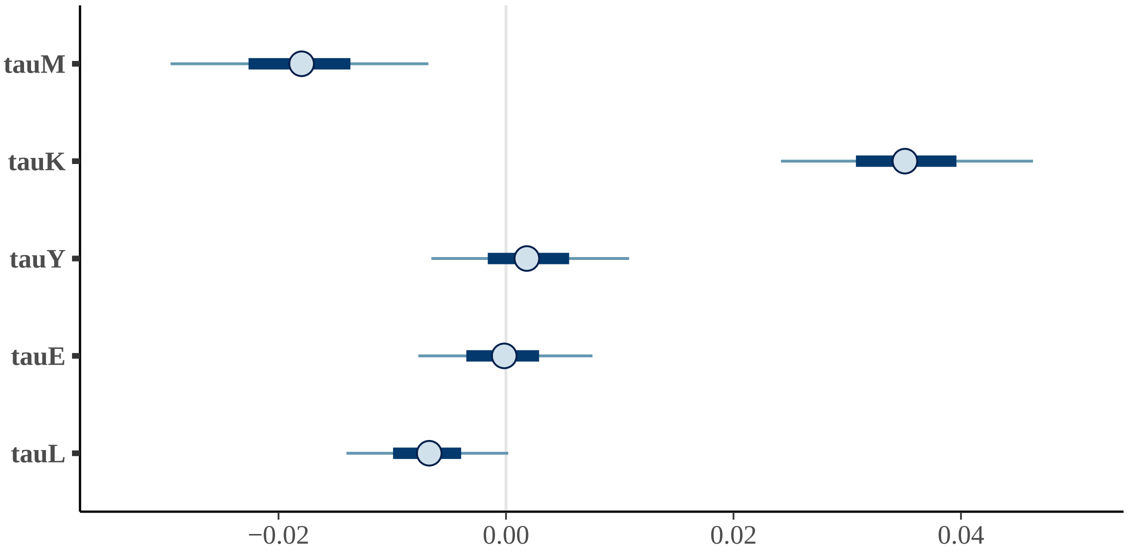


```
mcmc_intervals(fit, pars = c("alphaM", "alphaK", "alphaY", "alphaE", "alphaL"),
    prob_outer = 0.89)
```

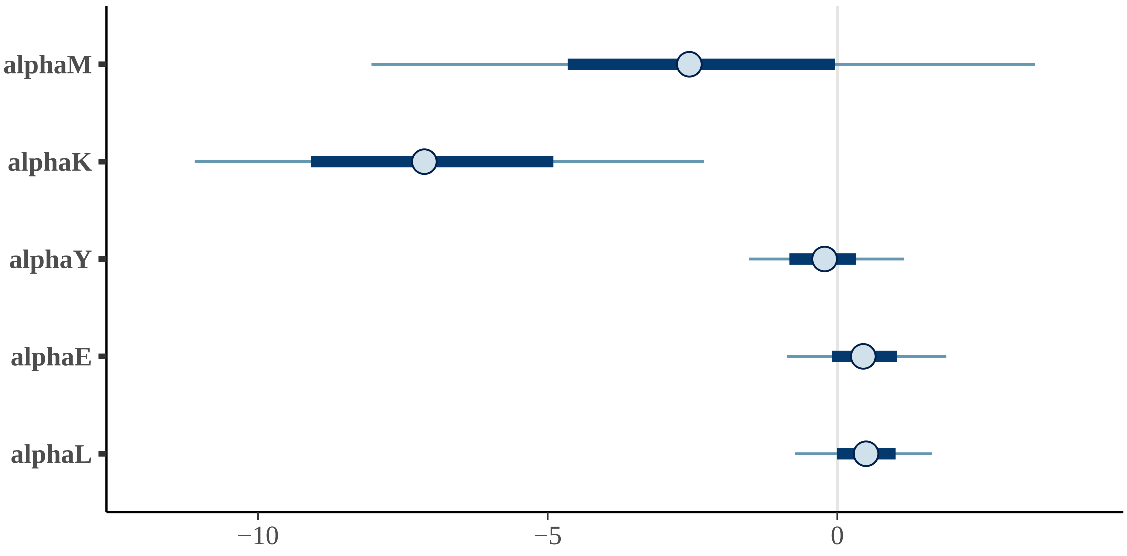

Figure 3: 89% posterior credible intervals for key model parameters describing effects due to animal state and environmental predictors.

Random effects

```
model_params <- "year_tau_RE"
years <- levels(factor(df_fit$year))

l_params <- rstan::extract(fit, pars = model_params)
m_RE <- l_params$year_tau_RE
colnames(m_RE) <- as.character(years)
df_RE <- data.frame(m_RE)
names(df_RE) <- as.character(years)

df_RE$Rep <- 1:nrow(m_RE)
df_RE <- gather(df_RE, key = Year, value = RE, 1:length(years))
df_summary_A <- df_RE %>%
  group_by(Year) %>%
  summarise(
    Days = 365*mean(RE),
    low95 = 365*quantile(RE, probs = 0.055), # 89% credible bounds
    upp95 = 365*quantile(RE, probs = 0.945)
  )
```

```
## `summarise()` ungrouping output (override with `.groups` argument)
```

```
p_3A <- ggplot(df_summary_A, aes(x = Year, y = Days))  +
  geom_hline(yintercept = 0, linetype = "dashed") +
  geom_point() +
  geom_errorbar(aes(ymin = low95, ymax = upp95), width = 0.2) +
  labs(y = "Change in\nmolting date (days)") +
  theme_bw() +
  theme(axis.text.x = element_text(angle = 30, hjust = 1, vjust=1))
```

```r
model_params <- c("year_alpha_RE")
years <- levels(factor(df_fit$year))

l_params <- rstan::extract(fit, pars = model_params)
m_RE <- l_params$year_alpha_RE
colnames(m_RE) <- as.character(years)
df_RE <- data.frame(m_RE)
names(df_RE) <- as.character(years)
df_RE$Rep <- 1:nrow(m_RE)
df_RE <- gather(df_RE, key = Year, value = RE, 1:length(years))
df_summary_A <- df_RE %>%
  mutate(RE2 = 100*RE/(4*(365))) %>%
  group_by(Year) %>%
  summarise(
    Days = mean(RE2),
    low95 = quantile(RE2, probs = 0.055), # 89% credible bounds
    upp95 = quantile(RE2, probs = 0.945)
  )
```

```
## `summarise()` ungrouping output (override with `.groups` argument)
```

```r
p_3B <- ggplot(df_summary_A, aes(x = Year, y = Days))  +
  geom_hline(yintercept = 0, linetype = "dashed") +
  geom_point() +
  geom_errorbar(aes(ymin = low95, ymax = upp95), width = 0.2) +
  labs(y = "Change in\nmolting rate (% per day)") +
  theme_bw() +
  theme(axis.text.x = element_text(angle = 30, hjust = 1, vjust=1))

plot_grid(p_3A, p_3B, ncol = 1, labels = c("A", "B"))
```

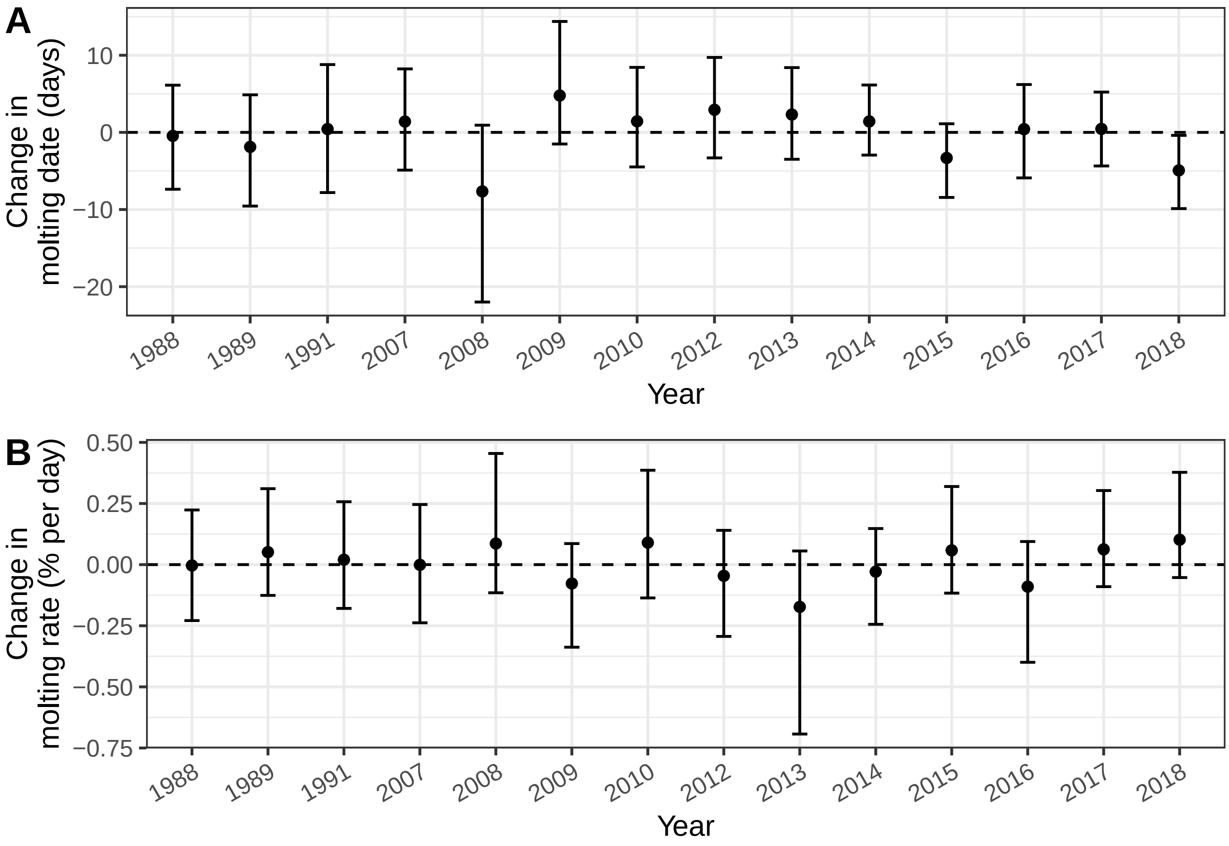

Figure 4: (A) Estimated annual deviates in the day of 50 molting relative to a female with no kid. Note that many of these estimates come from years with very few data, especially prior to 2008. (B) Estimated annual deviates in the rate of molting.

These deviates appear consistent with a t-distribution.

Predictions

Long-term trend

```
lat_predict = 0.0 # (60.871533 - Lat_mu) / Lat_sd # Yukon Wildlife Reserve
elv_predict = 0.0 # (753 - Elv_mu) / Elv_sd

model_params <- c("year_tau_RE", "tau0", "tauY", "tauL", "tauE")
years <- levels(factor(df_fit$year))

l_params <- rstan::extract(fit, pars = model_params)
m_RE <- l_params$year_tau_RE
colnames(m_RE) <- as.character(years)

df_tau_RE <- data.frame(m_RE)
names(df_tau_RE) <- as.character(years)

df_tau <- data.frame(
  Rep  = 1:nrow(m_RE),
```

```
  tau0 = as.vector(l_params$tau0),
  tauY = as.vector(l_params$tauY),
  tauL = as.vector(l_params$tauL),
  tauE = as.vector(l_params$tauE)
)

df_tau <- cbind(df_tau, df_tau_RE)

df_yr <- data.frame(
    year = as.integer(1988:2018)
  ) %>%
  mutate(
    z_yr   = (year - yr_mu) / yr_sd,
    low89  =  0.0,
    median = 0.0,
    upp89  = 0.0
  )

for (i in 1:nrow(df_yr)) {
  tau <- df_tau$tau0 + df_tau$tauY*df_yr$z_yr[i] +
    df_tau$tauL*lat_predict + df_tau$tauE*elv_predict
  df_yr[i, 3:5] <- quantile(tau, probs = c(0.055, 0.5, 0.945))
}

df_yr$low89date  <- as.Date(365*df_yr$low89,  origin = "2018-01-01")
df_yr$mediandate <- as.Date(365*df_yr$median, origin = "2018-01-01")
df_yr$upp89date  <- as.Date(365*df_yr$upp89,  origin = "2018-01-01")

df_offset <- data.frame(
    year = as.integer(years)
  ) %>%
  mutate(
    z_yr   = (year - yr_mu) / yr_sd,
    low89  =  0.0,
    median = 0.0,
    upp89  = 0.0
  )

for (i in 1:nrow(df_offset)) {
  tau <- df_tau$tau0 + df_tau$tauY*df_offset$z_yr[i] +
    df_tau$tauL*lat_predict + df_tau$tauE*elv_predict + df_tau[,i+5]
  df_offset[i, 3:5] <- quantile(tau, probs = c(0.055, 0.5, 0.945))
}

df_offset$low89date  <- as.Date(365*df_offset$low89,  origin = "2018-01-01")
df_offset$mediandate <- as.Date(365*df_offset$median, origin = "2018-01-01")
df_offset$upp89date  <- as.Date(365*df_offset$upp89,  origin = "2018-01-01")
```

```
df_offset <- filter(df_offset, year >= 1980)

ggplot() +
  geom_ribbon(data = df_yr,
    aes(x = year, ymin = low89date, ymax = upp89date), fill = "grey75") +
```

```
  geom_errorbar(data = df_offset,
    aes(x = year, ymin = low89date, ymax = upp89date),
    color = "black", width = 0) +
  geom_line(data = df_yr,
    aes(x = year, y = mediandate), color = "grey50", linetype = "dashed") +
  geom_point(data = df_offset,
    aes(x = year, y = mediandate), color = "black") +
  labs(x = "Year", y = "Date when 50% shed") +
  theme_bw()
```

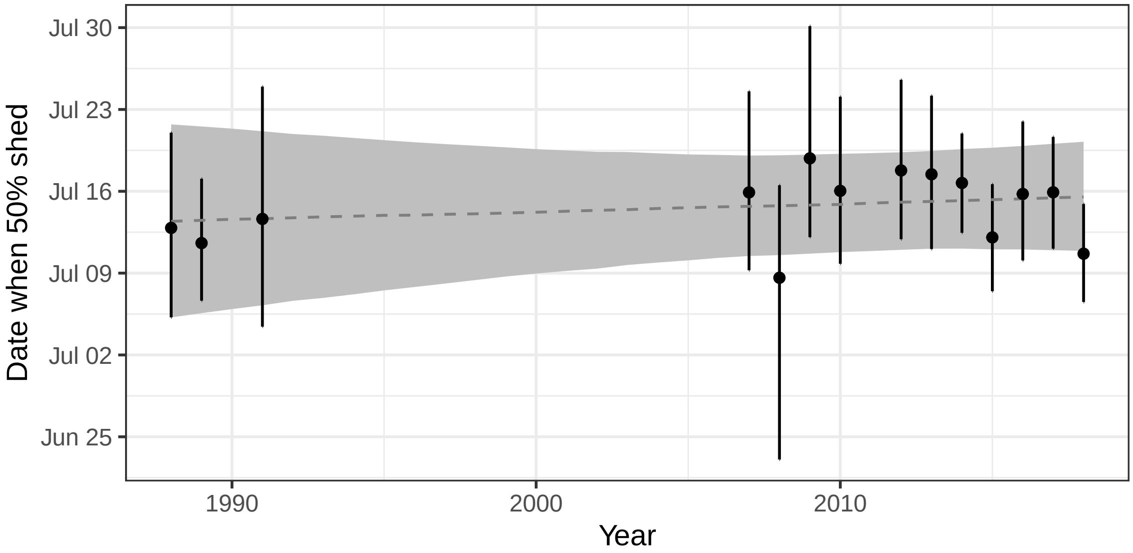

Figure 5: Predicted dates for females without kid (state FN) having shed 50% of their coat when at a site defined by z-transformed predictors being zero (i.e. latitude 49.14 and elevation 2025 m). Dashed line is the long-term trend and shaded region is the 89% CI. Estimated yearly fluctuations about the trend are also presented along with their 89% CI.

Within-season

```
# specify environmental conditions for the predictions
z_lat   <- 0.0 # (60.87 - Lat_mu) /Lat_sd
z_year  <- (2018 - yr_mu) / yr_sd
z_ele  <- 0.0 # (753 - Elv_mu) / Elv_sd

model_params <- c(
  "tau0",   "tauM",   "tauK",   "tauY",   "tauE",   "tauL",
  "alpha0", "alphaM", "alphaK", "alphaY", "alphaE", "alphaL")
l_params <- rstan::extract(fit, pars = model_params)

REPS    <- length(l_params$tau0) # samples to choose from
SAMPLES <- 100                   # random posterior samples
indxs   <- sample(1:REPS, SAMPLES, replace = FALSE)

# define temporal range of predictions
t_min   <- 100
t_max   <- 300
```

```r
v_t     <- seq(from = t_min, to = t_max, by = 1)/365
v_date  <- 365*v_t # as.Date(365*v_t, origin = "2018-01-01")
n_t     <- length(v_t)

df_low <- tibble(doy = v_date, FN = 0.0, FY = 0.0, MN = 0.0)
df_med <- tibble(doy = v_date, FN = 0.0, FY = 0.0, MN = 0.0)
df_upp <- tibble(doy = v_date, FN = 0.0, FY = 0.0, MN = 0.0)

for (i in 1:n_t) {
    t <- v_t[i]
  # extract posterior model parameters
  tau0   <- l_params$tau0[indxs]
  tauY   <- l_params$tauY[indxs]
  tauE   <- l_params$tauE[indxs]
  tauL   <- l_params$tauL[indxs]
  tauK   <- l_params$tauK[indxs]
  tauM   <- l_params$tauM[indxs]
  alpha0 <- l_params$alpha0[indxs]
  alphaY <- l_params$alphaY[indxs]
  alphaE <- l_params$alphaE[indxs]
  alphaL <- l_params$alphaL[indxs]
  alphaK <- l_params$alphaK[indxs]
  alphaM <- l_params$alphaM[indxs]

  # baseline date of shedding
  tau_i <- tau0 + tauE*z_ele + tauL*z_lat + tauY*z_year
  # baseline rate of shedding
  alpha_i <- alpha0 + alphaE*z_ele + alphaL*z_lat + alphaY*z_year

  logit_FN <- alpha_i*(t - tau_i)
  logit_FY <- (alpha_i + alphaK)*(t - tau_i - tauK)
  logit_MN <- (alpha_i + alphaM)*(t - tau_i - tauM)

  logit_FN <- exp(logit_FN) / (1.0 + exp(logit_FN))
  logit_FY <- exp(logit_FY) / (1.0 + exp(logit_FY))
  logit_MN <- exp(logit_MN) / (1.0 + exp(logit_MN))

  v_CI <- quantile(logit_FN, probs = c(0.055, 0.5, 0.945))
  df_low$FN[i] <- v_CI[1]
  df_med$FN[i] <- v_CI[2]
  df_upp$FN[i] <- v_CI[3]

  v_CI <- quantile(logit_FY, probs = c(0.055, 0.5, 0.945))
  df_low$FY[i] <- v_CI[1]
  df_med$FY[i] <- v_CI[2]
  df_upp$FY[i] <- v_CI[3]

  v_CI <- quantile(logit_MN, probs = c(0.055, 0.5, 0.945))
  df_low$MN[i] <- v_CI[1]
  df_med$MN[i] <- v_CI[2]
  df_upp$MN[i] <- v_CI[3]
}
```

```r
df_low <- gather(df_low, key = SK, value = low89, 2:4)
df_med <- gather(df_med, key = SK, value = med89, 2:4)
df_upp <- gather(df_upp, key = SK, value = upp89, 2:4)

df_CI <- df_low
df_CI$upp89 <- df_upp$upp89
```

```r
df_CI$SK <- factor(df_CI$SK)
levels(df_CI$SK)[levels(df_CI$SK)=="FN"] <- "FN (female, no kid)"
df_fit$SK <- factor(df_fit$SK)
levels(df_fit$SK)[levels(df_fit$SK)=="FN"] <- "FN (female, no kid)"
df_med$SK <- factor(df_med$SK)
levels(df_med$SK)[levels(df_med$SK)=="FN"] <- "FN (female, no kid)"

df_CI$SK <- factor(df_CI$SK)
levels(df_CI$SK)[levels(df_CI$SK)=="FY"] <- "FY (female with kid)"
df_fit$SK <- factor(df_fit$SK)
levels(df_fit$SK)[levels(df_fit$SK)=="FY"] <- "FY (female with kid)"
df_med$SK <- factor(df_med$SK)
levels(df_med$SK)[levels(df_med$SK)=="FY"] <- "FY (female with kid)"

df_CI$SK <- factor(df_CI$SK)
levels(df_CI$SK)[levels(df_CI$SK)=="MN"] <- "MN (male)"
df_fit$SK <- factor(df_fit$SK)
levels(df_fit$SK)[levels(df_fit$SK)=="MN"] <- "MN (male)"
df_med$SK <- factor(df_med$SK)
levels(df_med$SK)[levels(df_med$SK)=="MN"] <- "MN (male)"

ggplot() +
    geom_ribbon(data = df_CI,
        aes(x = doy, ymin = low89, ymax = upp89, fill = SK), alpha = 0.5) +
    geom_line(data = df_med, aes(x = doy, y = med89, color = SK)) +
    geom_point(data = filter(df_fit, SK %in% c("FN (female, no kid)",
        "FY (female with kid)", "MN (male)")),
        aes(x = doy, y = frac_shed, color = SK)) +
    scale_colour_manual(values=c("#980043", "#e7298a", "blue")) +
    scale_fill_manual(values=c("#980043", "#e7298a", "blue")) +
  labs(
    x = "Day of year",
    y = "Fraction shed",
    color = "Animal\nstate") +
    guides(fill = FALSE, color = FALSE) +
    facet_wrap( ~ SK) +
  theme_bw()
```

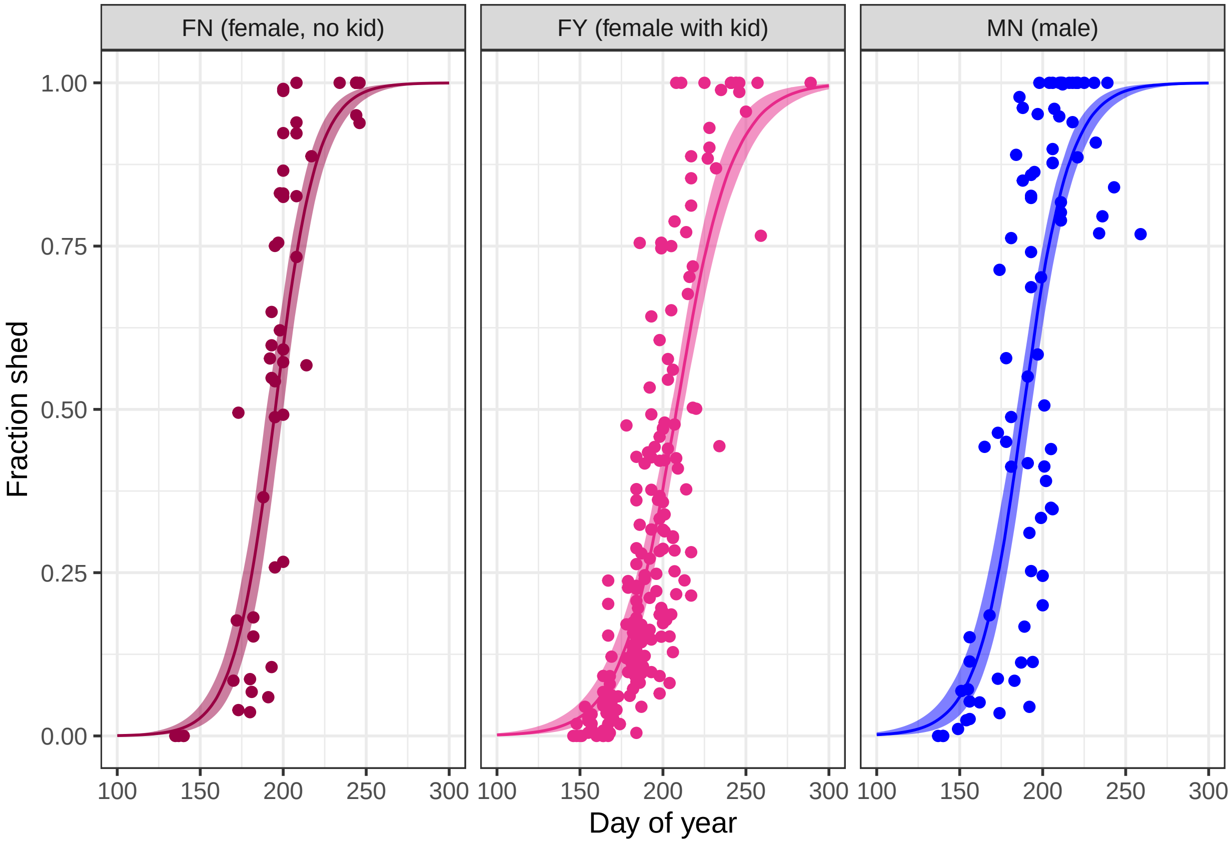

Figure 6: Observed and predicted shedding patterns. Panels correspond to the three animal states where sex and kid status are known. Shedding fractions for all photos where animal state was known are presented (points). The predictions are for 2018 at location defined by the z-transformed predictors being zero, which corresponds to latitude 49.14 and elevation 2025 m. The predictions also correspond to all random effect terms being set to zero. Solid lines depict the median shedding fraction and shaded regions are the associated 89% credible intervals.

Stan code

Stan code used to describe the shedding model.

```
writeLines(readLines("CSfitIncomplete.stan"))
```

```
## Warning in readLines("CSfitIncomplete.stan"): incomplete final line found on
## 'CSfitIncomplete.stan'
```

```
## // CaseRate.stan
## data {
##   int<lower=1>                  I;      // Number of animals
##   int<lower=1>                  N;      // Number of shedding blocks
##   int<lower=1>                  Y;      // Number of distinct years of data
##   int<lower=1,upper=6>    State[I];  // Animal state: FN, FY, FX, MN, XN, XX
##   real<lower=-5.0,upper=5.0> zt[I];  // z-day of year [0,1]
##   real<lower=-5.0,upper=5.0> ze[I];  // z-elevation
##   real<lower=-5.0,upper=5.0> zl[I];  // z-latitude
```

```
##   real<lower=-6.0,upper=5.0> zy[I];  // z-year [0.1,1.8]
##   int<lower=0,upper=Y>       iy[I];  // year index [1,Y]
##   int<lower=0,upper=N>        n[I];  // number of shed blocks [0,N]
## }
##
## parameters {
##   real <lower=0.2,upper=0.8>    tau0;       // base-line shed date
##   real <lower=15,upper=35>      alpha0;     // base-line shed rate
##   real <lower=-0.1,upper=0.1>   tauE;       // elevation effect on shed date
##   real <lower=-0.1,upper=0.1>   tauL;       // latitude effect on shed date
##   real <lower=-0.1,upper=0.1>   tauY;       // year effect on shed date
##   real <lower=-0.1,upper=0.1>   tauM;       // male effect on shed date
##   real <lower=-0.1,upper=0.1>   tauK;       // kid effect on shed date
##   real <lower=-5.0,upper=5.0>   alphaE;     // elevation effect on shed rate
##   real <lower=-5.0,upper=5.0>   alphaL;     // latitude effect on shed rate
##   real <lower=-5.0,upper=5.0>   alphaY;     // year effect on shed rate
##   real <lower=-12.0,upper=12.0>   alphaM;     // male effect on shed rate
##   real <lower=-12.0,upper=12.0>   alphaK;     // kid effect on shed rate
##   real <lower=0.001,upper=0.1>  sigmaTau;   // year-year random (shed date)
##   real <lower=0.001,upper=10.0> sigmaAlpha; // year-year random (shed rate)
##   real <lower=0.1,upper=100.0>  nuTau;      // df for t-dist (shed date)
##   real <lower=0.1,upper=100.0>  nuAlpha;    // df for t-dist (shed rate)
##   real <lower=0.001,upper=1.0>  phi;        // beta-binomial variation term
##
##   vector[Y] year_tau_RE;   // inter-annual random effect for shed date
##   vector[Y] year_alpha_RE; // inter-annual random effect for shed rate
## }
##
## model {
##   real logit_FN; // logit: females without kid
##   real logit_FY; // logit: females with kid
##   real logit_MN; // logit: males
##   real mu;       // mean shed fraction for observation
##   real a;        // beta-binomial parameter
##   real b;        // beta-binomial parameter
##   int  y;        // observed shed number
##   real tau_i;    // inter-annual random effect term: shed date
##   real alpha_i;  // inter-annual random effect term: shed rate
##
##   // priors (in additon to uniform restrictions set above)
##
##   tau0    ~ beta(2,2);
##   tauE    ~ normal(0,0.1);
##   tauL    ~ normal(0,0.1);
##   tauY    ~ normal(0,0.1);
##   tauM    ~ normal(0,0.1);
##   tauK    ~ normal(0,0.1);
##
##   alpha0  ~ normal(25,5);
##   alphaE  ~ normal(0,1.0);
##   alphaL  ~ normal(0,1.0);
##   alphaY  ~ normal(0,1.0);
##   alphaM  ~ normal(0,20.0);
##   alphaK  ~ normal(0,20.0);
```

```
##
##   sigmaTau   ~ exponential(10.0);
##   sigmaAlpha ~ exponential(1.0);
##   nuTau      ~ gamma(2, 0.1);
##   nuAlpha    ~ gamma(2, 0.1);
##
##   phi        ~ exponential(1.0);
##
##   // parameters = vector[J] u; u ~ normal(0, sigma_u);
##   // year_tau_RE   ~ normal(0, sigmaTau);   // year-year random effect
##   year_tau_RE   ~ student_t(nuTau, 0.0, sigmaTau);
##   // year_alpha_RE ~ normal(0, sigmaAlpha); // year-year random effect
##   year_alpha_RE   ~ student_t(nuAlpha, 0.0, sigmaAlpha);
##
##   for (i in 1:I) {
##      // set base-line tau and alpha
##     tau_i = tau0 + tauE*ze[i] + tauL*zl[i] + tauY*zy[i] +
##       year_tau_RE[iy[i]]; // day of year when 50% shed (female, no kid)
##     alpha_i = alpha0 + alphaE*ze[i] + alphaL*zl[i] + alphaY*zy[i] +
##       year_alpha_RE[iy[i]];  // max rate of shedding (female, no kid)
##     // modify tau and alpha based on animal state
##     if (State[i] == 1) { // FN
##       logit_FN = alpha_i*(zt[i] - tau_i);
##       mu = exp(logit_FN) / (1.0 + exp(logit_FN));
##       a = mu / phi;
##       b = (1.0 - mu) / phi;
##       y = n[i];
##       target += lgamma(N+1) + lgamma(a+b) + lgamma(y+a) +
##         lgamma(N-y+b) - lgamma(y+1) - lgamma(N-y+1) -
##         lgamma(a) - lgamma(b) - lgamma(N+a+b);
##     } else if (State[i] == 2) { // FY
##       logit_FY = (alpha_i + alphaK)*(zt[i] - tau_i - tauK);
##       mu = exp(logit_FY) / (1.0 + exp(logit_FY));
##       a = mu / phi;
##       b = (1.0 - mu) / phi;
##       y = n[i];
##       target += lgamma(N+1) + lgamma(a+b) + lgamma(y+a) +
##         lgamma(N-y+b) - lgamma(y+1) - lgamma(N-y+1) -
##         lgamma(a) - lgamma(b) - lgamma(N+a+b);
##     } else { // MN
##       logit_MN = (alpha_i + alphaM)*(zt[i] - tau_i - tauM);
##       mu = exp(logit_MN) / (1.0 + exp(logit_MN));
##       a = mu / phi;
##       b = (1.0 - mu) / phi;
##       y = n[i];
##       target += lgamma(N+1) + lgamma(a+b) + lgamma(y+a) +
##         lgamma(N-y+b) - lgamma(y+1) - lgamma(N-y+1) -
##         lgamma(a) - lgamma(b) - lgamma(N+a+b);
##     }
##   }
## }
```
